# Experimental and theoretical rationalization for the base pairing abilities of inosine, guanosine, adenosine, and their corresponding 8‐oxo‐7,8‐dihydropurine, and 8‐bromopurine analogues within A‐form duplexes of RNA

**DOI:** 10.1002/bip.23410

**Published:** 2020-11-20

**Authors:** Austin Skinner, Chou‐Hsun Yang, Kazuki Hincks, Haobin Wang, Marino J. E. Resendiz

**Affiliations:** ^1^ Department of Chemistry University of Colorado Denver Denver Colorado USA

**Keywords:** inosine within duplexes of RNA, 8‐oxoG, 8‐oxoI, 8‐oxoA, RNA oxidation, thermal stability of modified duplexes of RNA

## Abstract

Inosine is an important RNA modification, furthermore RNA oxidation has gained interest due, in part, to its potential role in the development/progression of disease as well as on its impact on RNA structure and function. In this report we established the base pairing abilities of purine nucleobases G, I, A, as well as their corresponding, 8‐oxo‐7,8‐dihydropurine (common products of oxidation at the C8‐position of purines), and 8‐bromopurine (as probes to explore conformational changes), derivatives, namely 8‐oxoG, 8‐oxoI, 8‐oxoA, 8‐BrG, and 8‐BrI. Dodecamers of RNA were obtained using standard phosphoramidite chemistry via solid‐phase synthesis, and used as models to establish the impact that each of these nucleobases have on the thermal stability of duplexes, when base pairing to canonical and noncanonical nucleobases. Thermal stabilities were obtained from thermal denaturation transition (*T*_m_) measurements, via circular dichroism (CD). The results were then rationalized using models of base pairs between two monomers, via density functional theory (DFT), that allowed us to better understand potential contributions from H‐bonding patterns arising from distinct conformations. Overall, some of the important results indicate that: (a) an anti‐I:syn‐A base pair provides thermal stability, due to the absence of the exocyclic amine; (b) 8‐oxoG base pairs like U, and does not induce destabilization within the duplex when compared to the pyrimidine ring; (c) a U:G wobble‐pair is only stabilized by G; and (d) 8‐oxoA displays an inherited base pairing promiscuity in this sequence context. Gaining a better understanding of how this oxidatively generated lesions potentially base pair with other nucleobases will be useful to predict various biological outcomes, as well as in the design of biomaterials and/or nucleotide derivatives with biological potential.

## Graphical abstract

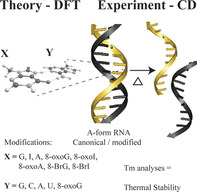

## 1 INTRODUCTION

Oxidative stress can lead to the formation of lesions at the nucleobases of DNA and RNA, with the latter gaining interest due to its potential role in the development/progression of disease.^[^ ^1^, ^2^, ^3^, ^4^, ^5^, ^6^ ^]^ Among the four canonical nucleobases, purine rings have the lowest redox potential (G < A < U ≈ C)^[^ ^7^, ^8^ ^]^ thus making them substrates that more readily undergo oxidation in the presence of reactive oxygen species (ROS), which may be generated from exogenous and/or endogenous sources.^[^ ^9^ ^]^ Furthermore, oxidative damage has been reported to impact RNAs of different sizes and with distinct functions, for example, miRNA,^[^ ^10^, ^11^ ^]^ rRNA,^[^ ^12^ ^]^ and mRNA.^[^ ^13^ ^]^ One of the products that can be generated in this process arises from the reaction between the ROS and the C8‐position of the purine rings, which leads to the corresponding 8‐oxo‐7,8‐dihydropurine derivatives (among other lesions).^[^ ^14^, ^15^ ^]^ Importantly, this functionalization induces changes locally, for example, conformational isomers,^[^ ^16^ ^]^ physical properties,^[^ ^17^ ^]^ or H‐bonding patterns^[^ ^18^ ^]^; as well as globally, for example, altered secondary structures and properties,^[^ ^19^ ^]^ distinct RNA‐protein interactions,^[^ ^20^ ^]^ or RNA‐small molecule complexes.^[^ ^21^ ^]^ Therefore it is important to understand how a distinct H‐bonding pattern, arising from the corresponding lesion, may lead to destabilization or conformational changes that potentially impact RNA structure and/or function. To this end we explored the base pairing capabilities of purines oxidized at the C8‐position and compared them to their corresponding canonical analogues, as control experiments, using duplexes of RNA as model structural motifs.

**FIGURE 1:**
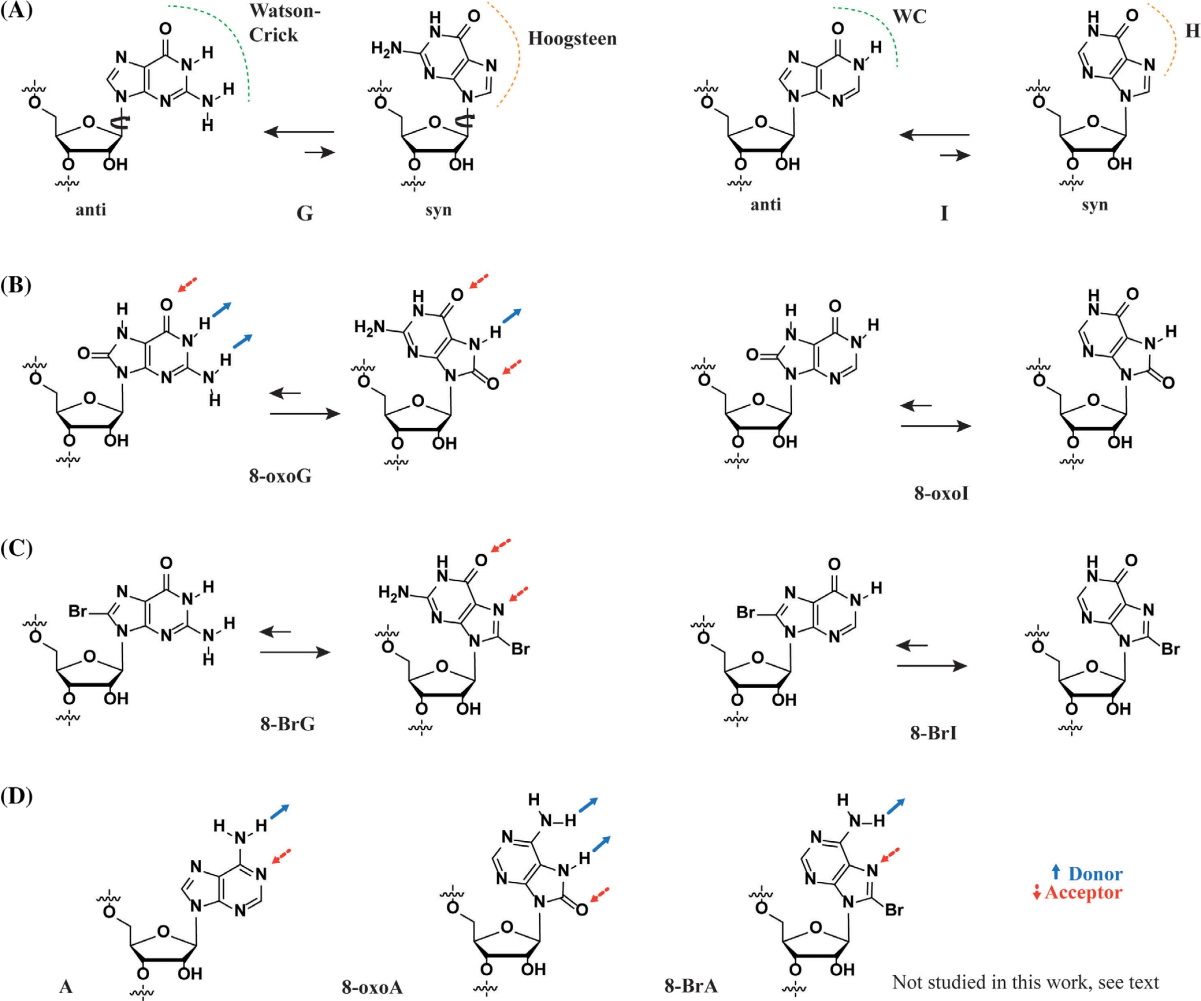
Structures, conformational changes, and H‐bonding patterns of, A, guanosine/inosine—G/I; B, 8‐oxo‐7,8‐dihydroguanosine/8‐oxo‐7,8‐dihydroinosine—8‐oxoG/8‐oxoI; C, 8‐bromoguanosine/8‐bromoinosine—8‐BrG/8‐BrI; and D, adenosine/8‐oxo‐7,8‐dihydroadenosine/8‐bromoadenosine. H, Hoogsteen; WC, Watson‐Crick

While our initial focus was on exploring the outcome of oligonucleotides containing 8‐oxoG, we were also interested in probing the impact of the C2‐exocyclic amine in guanine, or 8‐oxoguanine, which led us to explore duplexes containing inosine or 8‐oxoI. To this point inosine is an important modification that is observed in many biologically relevant processes,^[^ ^22^ ^]^ and that has been reported to code as G, A, or U in a context dependent manner,^[^ ^23^ ^]^ highlighting the importance of establishing its base pairing abilities. On the other hand 8‐oxoI is not expected to be biologically relevant, given that the oxidation potential of inosine is higher than that of A.^[^ ^24^ ^]^ However, this chemical modification can be used to learn about potential H‐bonding patterns and/or conformational changes around the glycosidic bond, as well as on the role of the C2‐exocyclic amine.^[^ ^25^, ^26^ ^]^ It is known that unmodified nucleosides exist in an equilibrium that favors the anti‐conformation and result in the H‐bonding patterns shown in Figure 1 (Watson‐Crick face). As depicted in Figure 1A, the lack of an exocyclic amine (for I) reduces the number of H‐bonds between the purine derivative and its potential Watson‐Crick base pair cytidine (C), which can be expected to result in decreased thermal denaturation transitions.^[^ ^27^ ^]^ On the other hand, functionalization of the C8‐position is known to switch the equilibrium in favor of the syn‐isomer and leads to a distinct H‐bonding pattern (Figure 1B).^[^ ^28^ ^]^ This conformational change is a result of steric hindrance between the C8‐group/atom and the C5′‐H atoms. With this in mind, we decided to use the corresponding 8‐bromo functionalized nucleosides to explore H‐bonding interactions, where the preferred syn‐conformation also exhibits a different H‐bonding pattern (Figure 1C).^[^ ^29^ ^]^ It is worth noting that both the C8‐oxo and C8‐Br substituted nucleosides are also capable of forming WC base pairs^[^ ^30^ ^]^ at the expense of disfavored interactions between this group and the C5′‐position, potentially resulting in overall thermal destabilization of a duplex containing the modified nucleotides. Lastly, the same behavior can be expected on the corresponding adenosine nucleosides, where A will differ from that expected on 8‐oxoA or 8‐BrA (Figure 1D). Oligonucleotides containing the 8‐BrA derivative could not be obtained in our hands (vide infra).

Overall, establishing the patterns and preferences for base pairing of the modified nucleosides explored herein is of potential biological relevance, and can also be of use in the design of other nucleoside‐based structural motifs or biomaterials. In fact, our laboratory is interested in probing the various base pairing abilities of these and other chemically modified nucleosides to generate aptamers of RNA with distinct selectivities.

## 2 EXPERIMENTAL PROCEDURES

### 2.1 General

The synthesis for the phosphoramidite of 8‐oxoG,^[^ ^19^ ^]^ 8‐oxoA,^[^ ^31^ ^]^ and 8‐oxoI/8‐BrI^[^ ^32^ ^]^ were previously reported by us and the same methodology was used to prepare all oligonucleotides, via solid‐phase synthesis. The synthesis of oligonucleotides of RNA containing 8‐BrA was not possible in our hands due to its transformation to the corresponding 8‐methylamine derivative (upon AMA‐deprotection of the synthesized oligonucleotide, Figure S‐4). It is possible that oligonucleotides containing this modified nucleoside can be attained by varying the deprotection conditions, however we have not been yet successful. UV‐vis spectroscopy of all small molecules was carried out on a Perkin Elmer λ‐650 UV/vis spectrometer using quartz cuvettes (1 cm pathlength). All experiments described herein were carried out in triplicate.

### 2.2 RNA synthesis

All oligonucleotides were obtained via solid‐phase synthesis using a 394 ABI DNA/RNA synthesizer. CPG supports and 2′‐O‐TBDMS phosphoramidites of U, A, C, and G were purchased from Glen Research. 0.25 M 5‐Ethylthio‐1H‐tetrazole in acetonitrile was used as the coupling reagent; 3% trichloroacetic acid in dichloromethane was used for deblocking; a 2,6‐lutidine/acetic anhydride solution was used for capping; and an iodine (0.02 M) in/THF/pyridine/water solution was used in the oxidation step (all purchased from Glen Research). Coupling times were adjusted to 10 minutes per nucleotide. Oligonucleotides (ONs) were deacetylated/debenzoylated/deformylated and cleaved from the CPG support in the presence of 600 μL of a 1:1 aq. methylamine (40%)/aq. ammonia (40%) solution with applied heat (60 °C, 1.5 hours). Desilylation was achieved using a mixture of N‐methylpyrrolidinone/triethylamine/HF (3:2:1, 350 μL) and heat (60 °C, 1.5 hours) followed by purification via electrophoresis (20% denaturing PAGE). C18‐Sep‐Pak cartridges (Waters) were used to desalt the purified oligonucleotides using 5 mM NH_4_OAc as the ion exchange buffer. The obtained oligonucleotides were concentrated under reduced pressure, dissolved in H_2_O, and used as obtained for subsequent experiments. Unmodified ONs (with one exception, see Figure S‐3) were purchased from IDT‐DNA or ChemGenes and, following quantification via UV‐vis, used without further purification. Table 1 displays the sequence of all the oligonucleotides used in this work.

**TABLE 1:** Sequences used in this work, where the nucleotide highlighted within the parenthesis() indicates the position that was systematically varied

#	Sequence (RNA)	#	Complementary strand (cRNA)	RNA:cRNA duplexes
1	5′‐AAG AG(G) GAU GAC	2	5′‐GUC AUC (G)CU CUU	1:2‐1:7
8	5′‐AAG AG(8‐oxoG) GAU GAC	3	5′‐GUC AUC (U)CU CUU	8:2‐8:7
9	5′‐AAG AG(8‐BrG) GAU GAC	4	5′‐GUC AUC (A)CU CUU	9:2‐9:7
10	5′‐AAG AG(I) GAU GAC	5	5′‐GUC AUC (C)CU CUU	10:2‐10:7
11	5′‐AAG AG(8‐oxoI) GAU GAC	6	5′‐GUC AUC (I)CU CUU	11:2‐11:7
12	5′‐AAG AG(8‐BrI) GAU GAC	7	5′‐GUC AUC (8‐oxoG)CU CUU	12:2‐12:7
13	5′‐AAG AG(A) GAU GAC			13:2‐13:7
14	5′‐AAG AG(8‐oxoA) GAU GAC			14:2‐14:7

### 2.3 RNA characterization (MALDI‐TOF)

All oligonucleotides were characterized via mass spectrometry (MALDI‐TOF MS) using equilibrated C18 Zip Tip pipette tips as follows: (a) wash tip with 50% acetonitrile (10 μL × 2); (b) equilibrate tip with 0.1% TFA (10 μL × 2); (c) load tip with sample (typically 100‐150 picomol); (d) wash tip with 0.1% TFA (10 μL × 2); (e) wash tip with water (10 μL × 2); (f) elute sample into matrix (10 μL of 25 mM‐2,4,6‐trihydroxyacetophenone monohydrate, 10 mM ammonium citrate, 300 mM ammonium fluoride in 50% acetonitrile); (g) spot directly onto MALDI plate. All analyses were carried out on an ABI 4800 Plus MALDI‐TOF/TOF mass spectrometer in positive mode (see acknowledgements and supporting information) and the spectra is available in Figures S1‐S4.

### 2.4 UV‐vis spectroscopy

All oligonucleotides were quantified via UV‐vis using a 1 mm path‐length with 1 μL volumes (Thermo Scientific Nano Drop Nd‐1000 UV‐vis spectrometer). Origin 9.1 was used to plot the spectra of monomers and oligonucleotides for comparison.

### 2.5 Circular dichroism (CD) spectroscopy and thermal denaturation transitions (*T*_m_)

CD spectra were recorded at various temperatures (PTC‐348W1 peltier thermostat) using Quartz cuvettes with a 1 cm path length. Spectra were averaged over three scans (325‐200 nm, 0.5 nm intervals, 1 nm bandwidth, 1 second response time) and background corrected with the appropriate buffer or solvent. Importantly, no secondary structure was detected for any of the oligonucleotides used herein, unless hybridized with its complement. Solutions containing the RNA strands had the following composition: 1.5 μM RNA, 1.5 μM cRNA, 5 mM MgCl_2_, 10 mM NaCl, 1 mM sodium phosphate‐pH 7.2. Thermal denaturation transitions (*T*_m_) were carried out by hybridization of the oligonucleotides of interest by heating to 90 °C followed by slow cooling to room temperature. *T*_m_ values were recorded at 270 nm with a ramp of 1 °C/min and step size of 0.2 with temperature ranges from 4 °C to 95 °C. A thin layer of mineral oil was added on top of each solution to keep concentrations constant at higher temperatures. Origin 9.1 was used to determine all Tm values and to normalize CD spectra of ss‐RNA and ds‐oligonucleotides for all RNA:cRNA duplexes. Samples representative of each RNA:cRNA duplex are shown in Figures S9‐S50. *T*_m_ values obtained in triplicate are shown in Tables S1‐S10.

## 3 RESULTS

**FIGURE 2:**
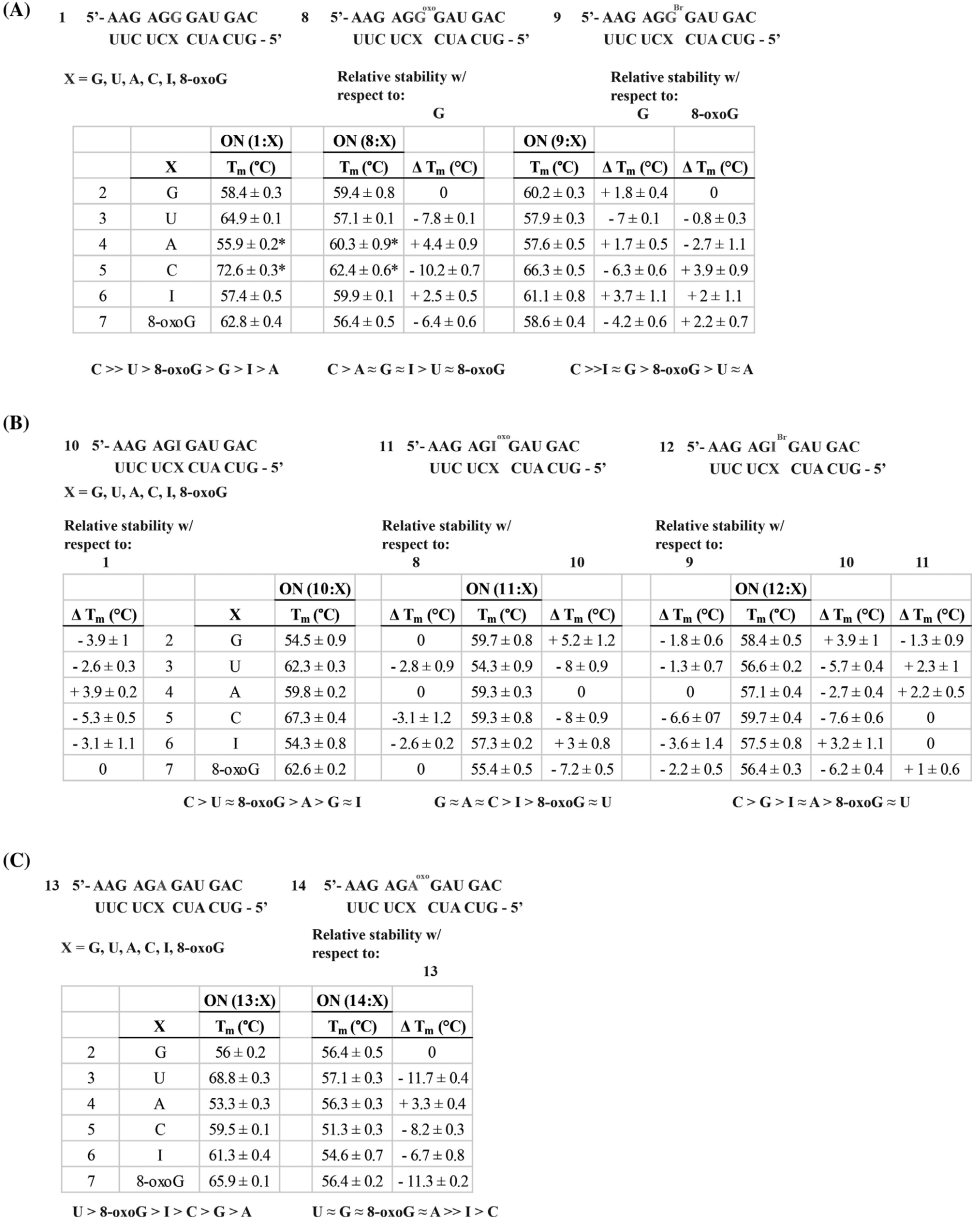
Thermal stabilities corresponding to RNAs containing (at the site of interest), A, guanosine, 8‐oxoguanosine, 8‐bromoguanosine; B, inosine, 8‐oxoinosine, 8‐bromoinosine; of, C, adenosine, 8‐oxoadenosine; base pairing with their corresponding cRNAs with the following variation at the corresponding position: G, U, A, C, I, or 8‐oxoG. Solutions were prepared in buffered solutions (100 mM NaCl, 10 mM sodium phosphate—pH 7.2, 5 mM MgCl_2_) containing each dodecamer in a 1:1 ratio at concentrations of app. 1.5 μM. All experiments were carried out in triplicate. Values denoted with an asterisk (*) were measured, and matched reported values.^[^ ^19^ ^]^ A (>>) sign was given to values with differences greater than 5 °C; and (≈) to differences < 1 °C

The sequence of the dodecamers is shown in Table 1, where position‐6 was systematically varied [RNA = 5′‐AAG AGZ GAU GAC‐3′, where Z = I, 8‐oxoI, 8‐BrI, G, 8‐oxoG, 8‐BrG, A, or 8‐oxoA] such that each oligonucleotide was independently set for hybridization with its corresponding complement [cRNA = 5′‐GUC AUC YCU CUU, where Y = G, U, A, C, I, 8‐oxoG] to yield all possible combinations that allowed us to arrive to the conclusions described herein. The main sequence (Figure 2) was chosen based on that of a previous report from our group that displays thermal denaturation transitions in the 70 °C range,^[^ ^19^ ^]^ which is a value that allowed us to record increments or drops in the corresponding thermal denaturation transition (*T*_m_) values accurately. In addition, thermodynamic parameters of RNA duplexes containing I:C base pairs were recently reported and showed that flanking Gs provided increased stability.^[^ ^33^ ^]^ The obtained values provided information that can be correlated with thermal stabilities and stabilization/destabilization arising from the presence of the lesions and modifications described herein. Circular dichroism (CD) was used to obtain all thermal denaturation transitions by recording the decrease in ellipticity at 270 nm as a function of applied heat. All duplexes displayed features that confirmed formation of the expected A‐form duplexes, that is, a band with negative ellipticity at ca. 210 nm and 240 nm along with another transition displaying positive ellipticity at 270 nm (Figures S9‐S51).

We initiated our studies by establishing the trends of ONs containing G, 8‐oxoG, or 8‐BrG using the same sequence context (**1**:**2**‐**1**:**7**, **8**:**2**‐**8**:**7** and **9**:**2**‐**9**:**7**) (Figure 2A). The thermal stability trends observed for oligonucleotides containing G or 8‐oxoG, were as follows: C > > U > 8‐oxoG > G > I > A, and C > A ≈ G ≈ I > U ≈ 8‐oxoG respectively. The trend for the base pairing abilities of the canonical series (**1**:**2‐1**:**7**) is in good agreement with established data, and the trend for 8‐oxoG (**8**:**2**‐**8**:**7**) also agrees with a previous report (C > A > G >> U)^[^ ^34^ ^]^ considering some of the differences in the report are within 1 °C and no error propagation is included. We then decided to compare these values to RNA duplexes containing 8‐BrG, where comparison in trends between this chemical modification, the oxidized lesion, and the canonical purine, provided information about likely H‐bonding patterns. The obtained trend (C > I ≈ G > 8‐oxoG > U ≈ A) varies from those measured for G or 8‐oxoG, while a 8‐BrG:C base pair is the most stable of the family, the formation of a 8‐BrG:G or 8‐BrG:I base pair displayed a relative stability within this family.

Next, we explored the thermal stabilities with the model oligonucleotide containing I (**10**) opposite G/U/A/C/I/8‐oxoG (**2**/**3**/**4**/**5**/**6**/**7**, respectively). Interestingly, upon close inspection of literature we discovered that thermal denaturation transitions in RNA are only available for a handful of scenarios, specifically those involving an I:U,^[^ ^35^ ^]^ or an I:C^[^ ^33^ ^]^ base pair, where I:U base pairs have been shown to distort the RNA duplex. As shown in Figure 2B, the patterns observed for base pairing interactions with I displayed a trend that favored base pairing with pyrimidines or 8‐oxoG (C > U ≈ 8‐oxoG > A > G ≈ I) over the corresponding purine‐based nucleobases. This trend differs from that reported on deoxyinosine within duplexes of DNA, where base pairing ability of dI has been reported to be C > A > T ≈ G > I in two different sequence contexts.^[^ ^36^, ^37^ ^]^ Most notably is the change involving a preference for U in RNA and A in DNA, as the second most stable base pair. Possibly explained due to overall structural changes within the duplex (A‐form vs B‐form),^[^ ^38^ ^]^ although more examples are necessary to assign this as a general trend. Another important trend can be observed upon comparison with values within the G‐family (**1**:**2**‐**1**:**7**), where the absence of an exocyclic amine displays depressed values in every case except with A, which showed an increase in thermal stability of app. +4 °C. This result is in agreement with favorable interactions that occur in biochemical processes such as reverse transcription,^[^ ^32^ ^]^ where RNA templates containing inosine are able to incorporate dA on their corresponding DNA primer and allow cDNA synthesis, albeit at lower efficiency rates and where the G‐containing RNA analogue does not display this behavior. This result may be interpreted as a lack of H‐bonding interactions from the excocyclic amine in inosine, or forced conformational changes to fit a favorable H‐bonding pattern (vide infra).

In order to gain more information about the exocyclic amine in the presence of oxidative lesions, we compared the results with the corresponding RNA duplexes containing 8‐oxoI (**11**:**2**‐**11**:**7**, Figure 2B, middle) to observe a change in the corresponding trends of 8‐oxoI base pairing (C ≈ A ≈ G > I > 8‐oxoG ≈ U). While the preference for stable base pairing interactions between 8‐oxoI and A or C can be expected, due to a similar H‐bonding pattern with 8‐oxoG, the thermal stabilization when compared to G or I came as a surprising result; where the discrepancy between these two nucleosides indicates the impact of the C2‐exocyclic amine on the duplex overall. All of the proposed base pairs can be justified with 8‐oxoI existing as a syn‐isomer (vide infra). In addition, 8‐oxoG and U can be seen as two nucleobases with similar H‐bonding patterns, a fact that has been observed before in their mode of binding,^[^ ^39^ ^]^ thus justifying the trends with A or C. Comparison with duplexes containing 8‐oxoG, displayed destabilization with the pyrimidine‐containing complements, as well as with that containing 8‐oxoG. To complete the inosine series and potentially learn about H‐bonding patterns, we prepared an RNA dodecamer containing 8‐BrI and measured the thermal stabilities for the corresponding duplexes (trend = C > G ≈ I > A > U ≈ 8‐oxoG). Interestingly, comparison with I displayed stabilization only when a G or I were present, which is the same result as that obtained for 8‐oxoI, and suggests that this interaction is favored when either 8‐oxoI or 8‐BrI are in the syn‐conformation. In designing experiments, it is important to point out that the buffer systems for RTn experiments with biotechnology applications, for example, sequencing, are carried out at pH values of ca. 8.3 (suggested by manufacturer providing reverse transcriptases). While this pH is not expected to affect the base pairing properties of canonical nucleosides (including inosine), slight spectroscopic changes on the monomer of 8‐oxoI were observed (Figures S5‐S8). To ensure that these conditions would not affect the protonation state of 8‐oxoI, we obtained the T_m_ values for the 8‐oxoI family (**8**:**2**‐**8**:**6**) at pH of 8.5 to observe values that were within error of those obtained at physiological conditions (Figure S3).

We then decided to compare results between A and 8‐oxo‐7,8‐dihydroadenosine (8‐oxoA) within the same sequence context, duplexes **13**:**2**‐**13**:**7** and **14**:**2**‐**14**:**7** (Figure 2C). Consistent with established trends, adenosine formed base pairs with the following preference (U > 8‐oxoG > I > C > G > A), while the trend for 8‐oxoA was: U ≈ G ≈ 8‐oxoG ≈ A > I > C. Notably, 8‐oxoA base paired with relative stability to all nucleobases (except C or I) with comparable thermal stabilities. These trends vary with those reported previously for RNA:DNA duplexes [A: T >> G >> C ≈ A; 8‐oxoA: T > G >> C ≈ A^[^ ^40^ ^]^], an aspect that requires further inspection in other sequence contexts given that the trends between RNA:RNA and RNA:DNA, which form A‐form duplexes, can be expected to be similar.

### 3.1 Theoretical models—H‐bonding contributions

The contribution from the hydrogen bonding was investigated by applying electronic structure calculations, which were performed using the quantum chemical program package Gaussian G16^[^ ^41^ ^]^ and Q‐Chem 5.^[^ ^42^ ^]^ The H‐bonding energy was evaluated as the free energy difference between the dimer and the sum of two monomers, all fully optimized in structures. Geometry optimizations were carried out employing the hybrid functional B3LYP with Grimme's empirical dispersion correction DFT‐D3(BJ),^[^ ^43^, ^44^ ^]^ and the 6‐31+G* basis set. To account for the free energy correction, standard normal mode analysis and frequency calculations were performed at the same level of theory. The solvation free energies were obtained using the polarizable continuum model (PCM) with water as the solvent. In addition, to calculate accurate single point electronic energies, second order Møller‐Plesset (MP2) perturbation theory^[^ ^45^ ^]^ and a larger basis set 6‐311++G** were used. In the SI we include some other results employing various DFT functionals and basis sets. Using MP2 theory as a gauge for a limited number of compounds, we decided the level of theory here is best compromise between accuracy and computational cost. Despite active research on the subject, treating hydrogen bonds accurately with DFT remains a challenging task.^[^ ^46^ ^]^

Using this methodology, we considered the following in order to establish a plausible/preferred base pair, where lower energies indicate more stable base pairs: (a) structural information (planarity; C1′─C1′ distance; and number of H‐bonds) as the major means of estimating stability; (b) the calculated free energies of formation for base pairs, which serve as a partial (sometimes major) reference contribution to the overall base pair stability; and (c) the effect of backbone and π‐stacking is neglected in the model. Planarity was determined by measuring dihedral angles among the atoms participating in H‐bonding interactions (0° or 180°), where most of the base pairs failing this category displayed distortions that were visibly out‐of‐planarity. C1′─C1′ distances were measured and all reasonable base pairs fell in the 10‐11 Å range, in agreement with a base pair ability to fit within a regular helix.^[^ ^47^ ^]^ Base pairs that displayed distances outside of this range were considered as less probable. H‐bonding interactions were measured and qualified as those closer than 2 Å between the donor and the acceptor, where reasonable base pairs contained two or more of such interactions. It is known that interactions between Watson‐Crick pairs and other biopolymers require two H‐bonds to achieve fidelity, and that recognition from the minor groove side is not affected by base pair reversals.^[^ ^48^ ^]^ It is important to note that the energetic contributions and differences among base pairs do not take into consideration structural disruptions, on the duplex, arising from conformational changes or other structural factors imposed by a modified base pair or base pair mismatch. The measured energy differences are the result of H‐bonding interactions and electronic factors arising from different substituents on the purine rings.

**FIGURE 3:**
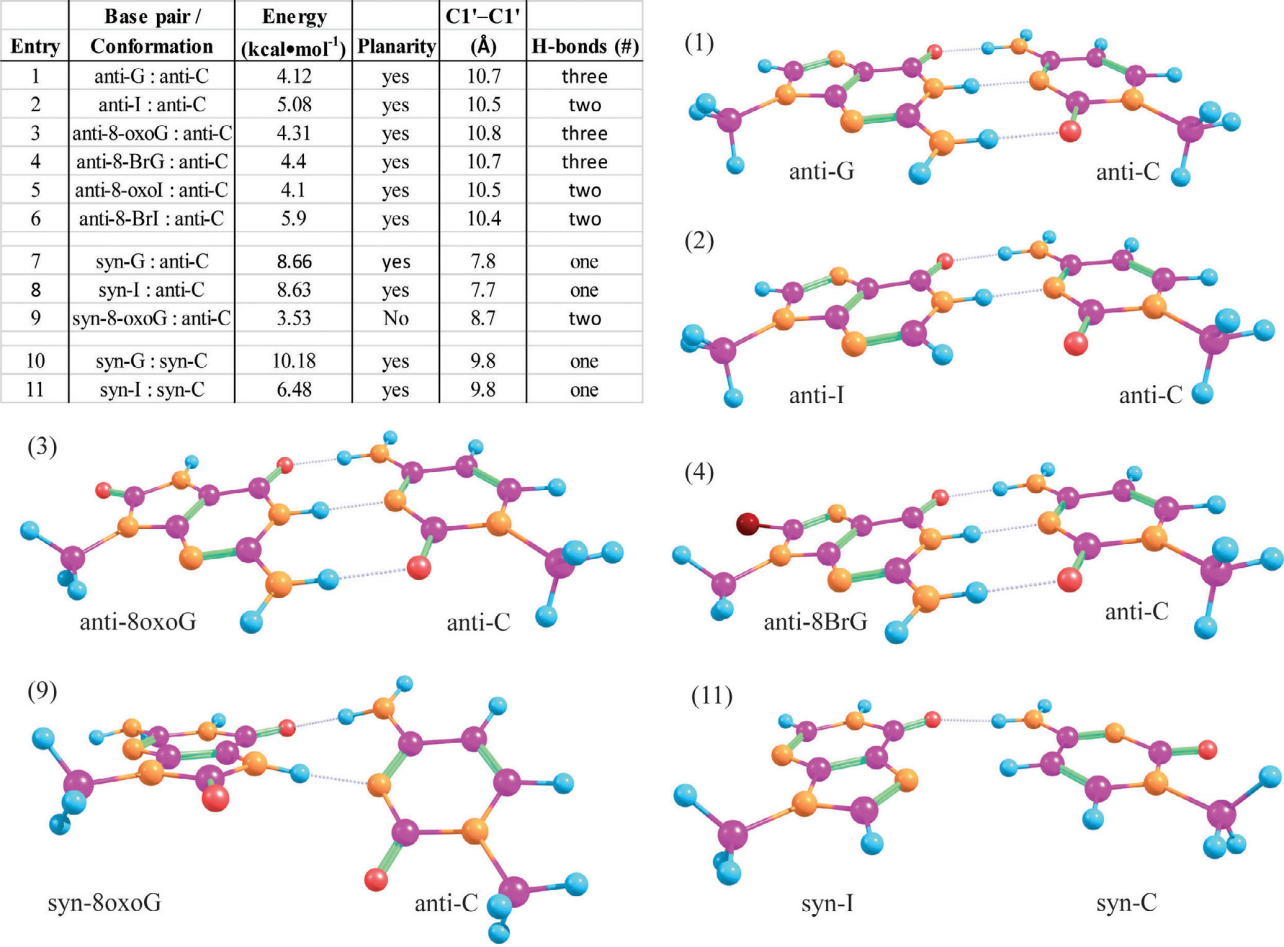
Theoretical models indicating the energy differences arising from the sum of two monomers on purine:C base pairs. The structures for select models are represented by the entry number (1‐4). Color code: C = magenta, O = red, H = blue, N = orange, Br = dark red

We initiated our analyses by building a G:C WC base pair, and explored anti‐/syn‐conformations; which were then compared to their corresponding purine derivative analogues (Figure 3). Gratifyingly, the modeling that was carried out validated our approach as follows: (a) comparison between an anti‐G: anti‐C and an anti‐I: anti:C (entries 1, 2) led to destabilization of the base pair in the latter, due presumably to the missing H‐bond arising from the lack of an exocyclic amine in inosine (drop in stability of ‐ 0.96 kcal mol^−1^); (b) all anti: anti base pairs displayed planarity and C1′‐C1′ distances in the expected ranges (entries 1‐6); (c) altering the conformational arrangement around the glycosidic bond (anti‐/syn‐) led to increased energies or disruption of planarity or C1′‐C1′ distances out of the optimal range (entries 7‐11). This is also in direct agreement with a drop of 5.3 °C on the *T*_m_ analyses, with the same trend being observed upon comparison of 8‐oxoG:C and 8‐oxoI:C or 8‐BrG:C and 8‐BrI:C base pairs. Interestingly, the modeling indicated that the modification at the C8‐position of the purine rings induced destabilization of the base pair. Since the contributions in this model arise strictly from electronic aspects, that is, induced dipole moments, or formation of electronic isomers, then, it is reasonable to expect that discrepancies between experimental results (thermal denaturation transitions) and the modeling, arise from structural changes imposed by these groups on the overall duplex. Furthermore, we explored syn‐conformations on both purine and pyrimidine rings, to observe that all options failed at least one of the three categories suggesting a stable base pair. Notably, the syn‐8oxoG: anti‐C base pair (entry 9) displayed a low energy that would correspond to a stable interaction, however, the base pair is notably out‐of‐planarity and the C1′‐C1′ distance is closer than the optimal range. Similarly a syn‐I: syn‐C base pair (entry 11) displayed planarity but with a higher energy than its anti:anti analogue (entry 2), resulting in higher energy, C1′‐C1′ distance out of range, and only one H‐bond interaction. The only values that came in an unexpected trend were those coming upon comparison of 8‐oxoG or 8‐oxoI base pairing with C (entries 3, 5), where the additional H‐bond on 8‐oxoG should result in a lower energy. In this regard, it is possible that this may be a possible error, on this base pair, with the chosen method.

**FIGURE 4:**
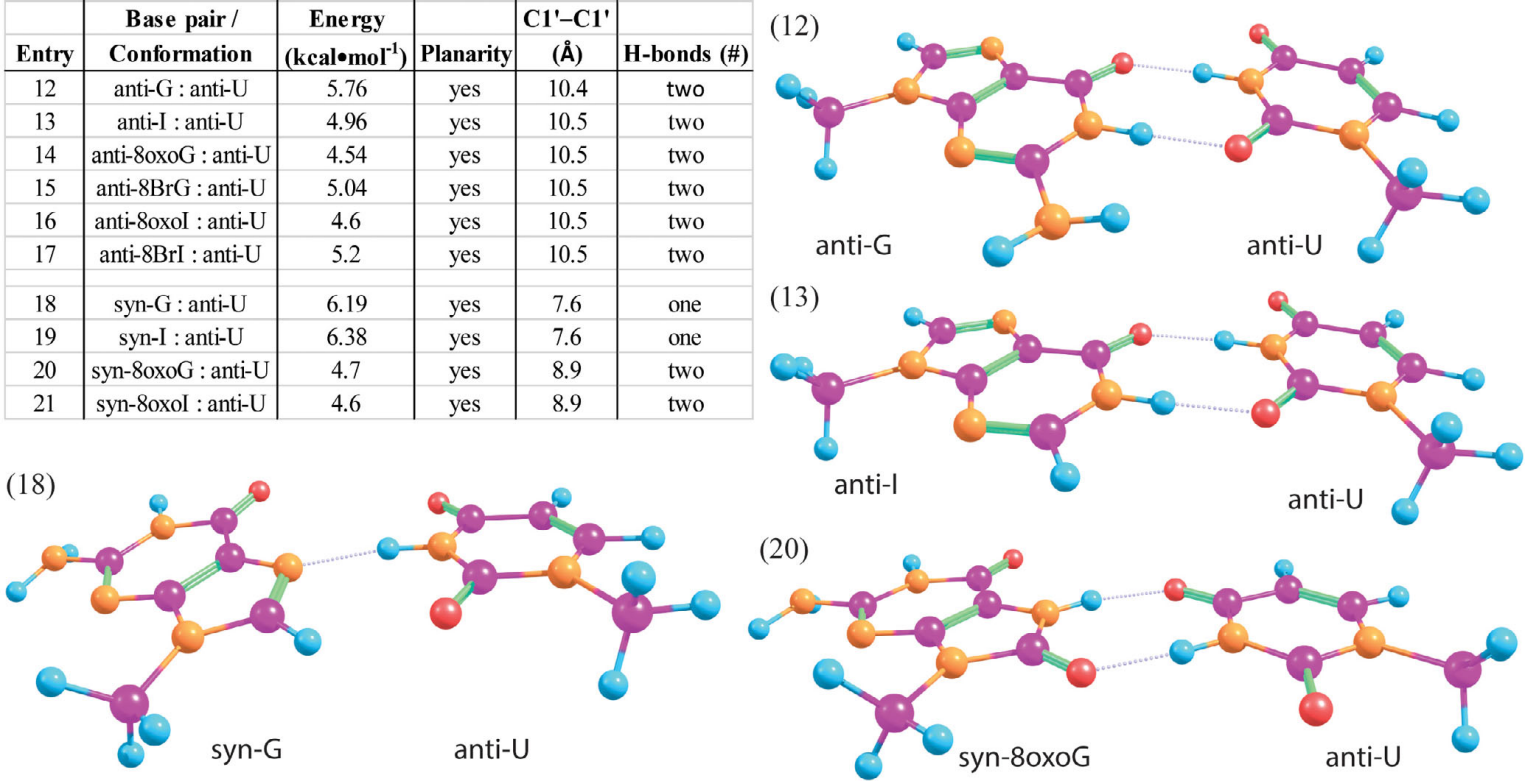
Theoretical models indicating the energy differences of purine:U base pairs. Color code: C = magenta, O = red, H = blue, N = orange, Br = dark red

Once the method was validated, we explored differences involving the purine derivatives with the other pyrimidine nucleobase, that is within the G:U family (Figure 4). In agreement with experimental values, the anti‐G: anti‐U base pair displayed higher energy from that of the analogous I: U base pair (entries 12, 13). Since inosine is expected to exist in the same conformation as guanosine, it is likely that the difference that is reflected in the *T*_m_ values is a result of electronic‐, or steric effects imposed by the C2 exocyclic amine. Unexpectedly, the energies corresponding to the anti: anti base pair were lower for the 8‐oxo derivatives (entries 14, 16), while the *T*_m_ values indicated the opposite trend (G:U ≈ 65 °C/8‐oxoG:U ≈ 57 °C and I:U ≈ 62 °C/8‐oxoI:U ≈ 54). This suggests that, while it is likely that the 8‐oxopurine derivatives base pair in an anti‐conformation, the C8 substituent may induce a large disruption within the RNA duplex. In addition, any of the combinations where a syn‐purine was base pairing with anti‐U failed at least one of the categories that we placed for a favorable base pairing interaction. The syn‐conformer of the pyrimidine rings were not explored given the lack of H‐bonds exposed by the C‐H face.

**FIGURE 5:**
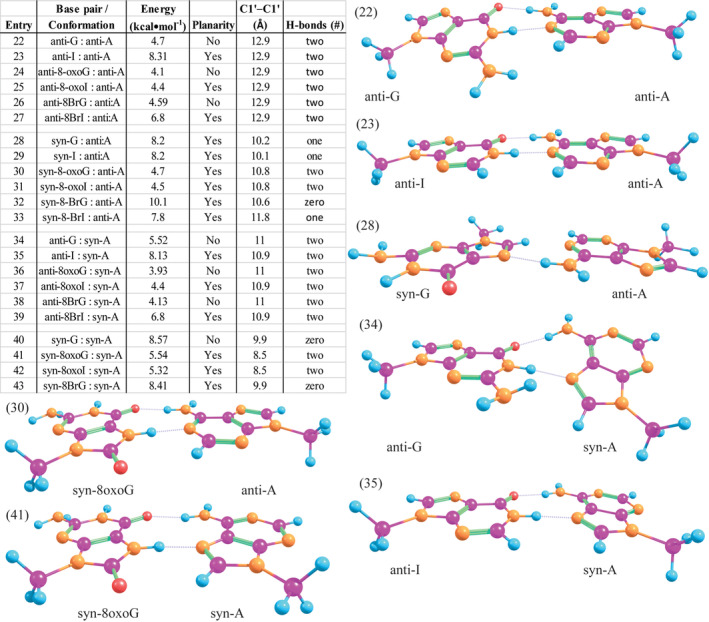
Theoretical models indicating the energy differences arising from purine:A base pairs. Color code: C = magenta, O = red, H = blue, N = orange, Br = dark red

We then turned the attention to the purine: purine base pairs (Figure 5). Based on previous reports we expected for G:A and I:A to display stabilization for the latter, given that reverse transcription has shown for I:A base pairs to form seemingly stable base pairs. Interestingly, comparison between G:A and I:A base pairs led to the conclusion that the exocyclic amine has an adverse impact on the formation of the base pair, where the anti‐G: anti‐A base pair does not display planarity, which contrasts the anti‐I: anti‐A base pair (entries 22, 23). This is in agreement with experimental results where the corresponding I:A containing duplex displays a higher thermal stability (G:A ≈ 56 °C/I:A ≈ 60 °C). However, the C1′‐C1′ distance for these cases is out of the optimal range. This led us to explore other conformations, where comparison between anti‐G: syn‐A and anti‐I:syn‐A (entries 34, 35) displayed the same impact of the exocyclic amine and led to planarity in the latter case. The same behavior was observed on the 8‐oxo and 8‐bromo derivatives (entries 36‐39). In addition, the C1′‐C1′ distances in these conformational base pairs were in the optimal range. As shown in Figure 5, none of the other base pairs met all categories for a plausible base pair.

**FIGURE 6:**
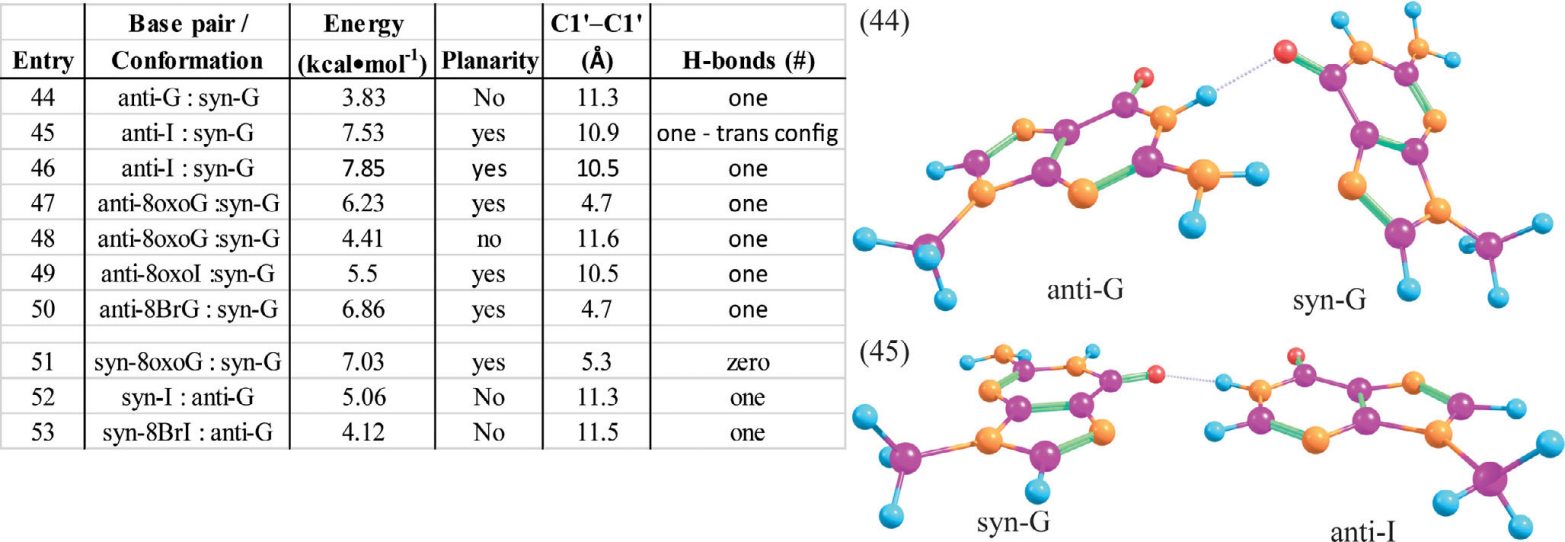
Theoretical models indicating the energy differences arising from purine:G base pairs. Color code: C = magenta, O = red, H = blue, N = orange, Br = dark red

Lastly, we explored the G:G base pairing interactions (Figure 6). As with other examples, the presence of the exocyclic amine placed a negative contribution toward the formation of a stable base pair. Generally, these combinations represented the least stable base pairs with the 8‐oxopurine derivatives as the ones inducing the higher thermal stabilities, experimentally. None of the combinations that were deemed as feasible models yielded structures fitting into the three set categories, and only scenarios where one stable H‐bond was present were calculated.

## 4 DISCUSSION

### 4.1 Thermal denaturation transition trends and plausible H‐bonding

The obtained *T*_m_ trends (Figure 2) for RNA duplexes containing G, I, or A, yielded the highest thermal stability with their expected WC base pairs C or U, thus a detailed explanation was not necessarily warranted. However, there were some other trends, with the oxidatively derived lesions or I, for which further analysis was carried out. To this end, we combined the experimental data with modeling, via DFT. We took into consideration the Cis‐orientation as the preferred geometry.^[^ ^49^ ^]^ In addition, we took established C1′‐C1′ internucleotidyl distances from previous reports, with distances between 10 and 11 Å as likely base pair geometries/conformers.^[^ ^50^ ^]^

**FIGURE 7:**
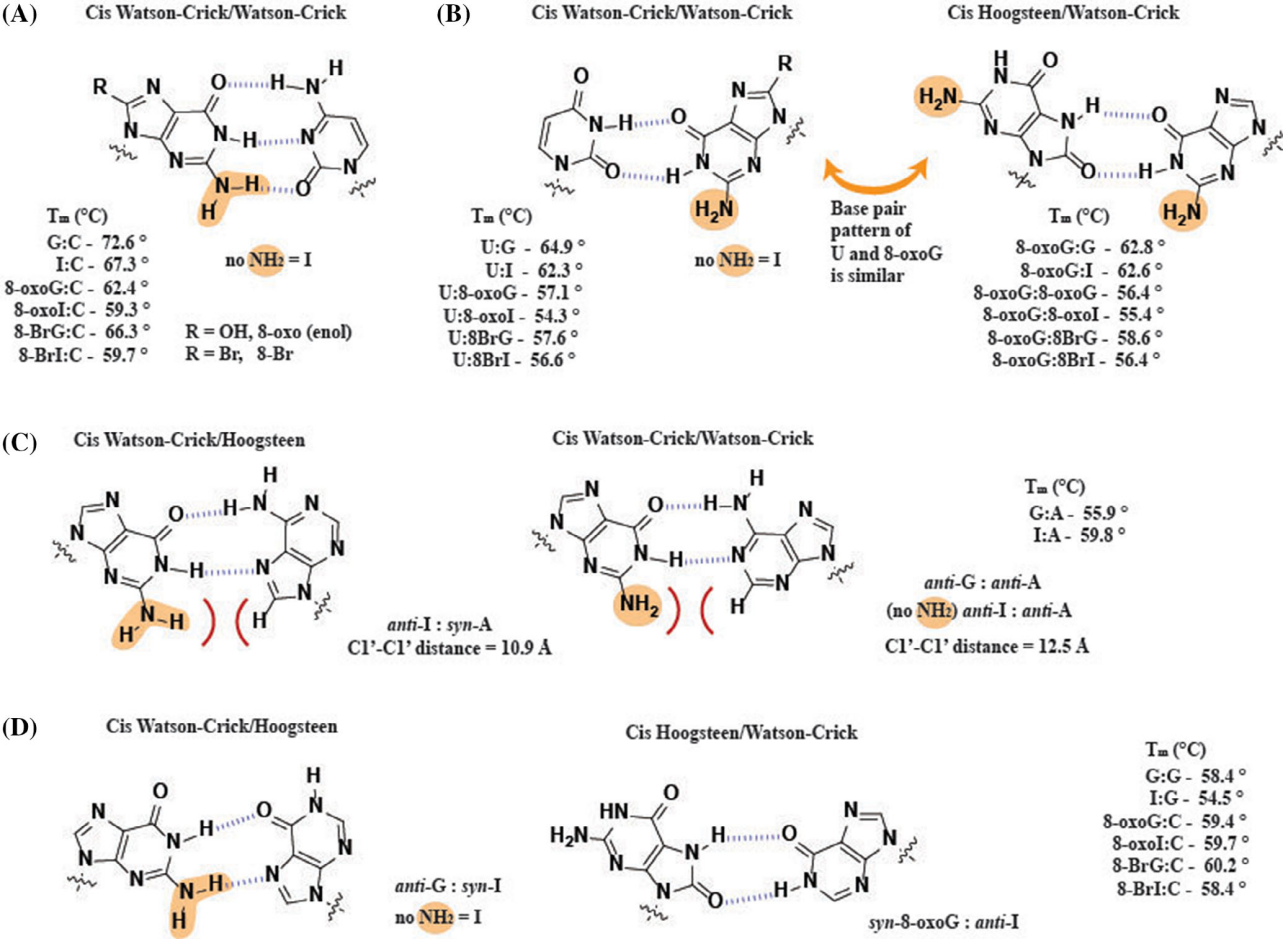
Proposed base pairs along with the thermal denaturation transitions obtained in this work

*WC base pairing (G:C)*. As expected, comparing the thermal stability of RNA duplexes containing purine rings lacking the C2‐exocyclic amine to their analogues containing this functional group, led to decreased *T*_m_ values in each case (Figure 7A). Furthermore, the impact arising from an additional H‐bonding interaction was in the 1 kcal⋅mol^−1^ range and is within previously reported experimental values of app. 2 kcal⋅mol^−1^.^[^ ^33^ ^]^ The difference between the base pairs, where the 8‐bromopurine derivatives is higher, suggests that the bromine has a larger adverse impact on the duplex structures, followed by the corresponding C8‐Oxidized analogues. Furthermore, the results obtained from modeling suggest that the functionalization at the C8‐position does not have a large impact on H‐bonding, and that exposure of this group on the major groove of the duplex may be responsible for the thermal destabilization observed across each family, that is, comparing duplexes containing G, 8‐BrG, or 8‐oxoG.

*Wobble pairing (G:U)*. The base pairs formed between the purines and U displayed the highest thermal stabilization for the G:U base pair (Figure 7B). However the modeling displayed a different trend, where the highest energy was observed on the G:U base pair, suggesting that factors involving the substitution on the purine ring (differing from G) are having an impact on the overall duplex structure, for example, intranucleotidyl interactions between the C8‐group and the C5′‐position, or adverse interactions arising from the presence of the C2‐exocyclic amine. Other reports have shown that an I:U base pair is able to adopt various geometries in a sequence dependent manner,^[^ ^51^ ^]^ which grants probing different sequences to establish this trend as general. Another observation that is noteworthy regards to the duplexes containing a G:8‐oxoG base pair, which displayed similar values to those measured on the G:U analogues and highlights the ability of an 8‐oxoG lesion to mimic the base pairing of U (Figure 7B).

*Sheared pairing (G:A)*. The only base pair that exhibited increase thermal stability upon destitution of the exocyclic amine (G‐to‐I exchange) was in the case where base pairing occurred with A (Figure 7C). Besides the importance of G:A base pairs in various biological contexts^[^ ^52^ ^]^ our laboratory recently reported on a case where reverse transcription allowed for the incorporation of dA opposite I, but not opposite G,^[^ ^32^ ^]^ this case was of particular interest to us. The modeling provided useful data in this regard, where the amine group destabilized a G:A base pair in conformations that were more plausible (Figure 5, entries 22 and 34) and inhibited the formation of a planar base pair. On the other hand, formation of an I:A base pair restored the geometry to a planar motif in both cases (entries 23 and 35) with the anti‐I:syn‐A conformation as the most likely interaction. This assignment is also in agreement with the fact that this arrangement has been observed within crystals of DNA duplexes^[^ ^53^ ^]^; as well as other reports.^[^ ^54^ ^]^ Gratifyingly, all experimental and modeling data supported previous observations (also referenced throughout the text) where formation of an 8‐oxoG:A base pair is favored when the 8‐oxoG nucleotide is in the syn‐conformation. Furthermore the impact of the exocyclic amine is not relevant, as the *T*_m_ and calculated energies (entries 30‐31) of an 8‐oxoI:A base pair were equivalent.

*G:G base pairing*. The trend between a G:G and a G:I base pair favored thermal stability in the former (Δ*T*_m_ app. 4 °C); and interestingly, both 8‐oxo and 8‐bromo derivatives base pairing with G displayed similar thermal stabilities (Figure 7D). This suggests that a combination of an anti‐ and a syn‐conformation give rise to this interaction. This arrangement has been previously observed in crystalline duplexes of RNA^[^ ^55^ ^]^ and established in disease models.^[^ ^56^ ^]^ Since the I:G base pair is the only one not within the range, this suggests that the exocyclic amine plays a role in this base pairing family, where G may enable an easier anti‐syn conformational change. As illustrated in Figure 7B, H‐bonding in an 8‐oxoG:I base pair can be rationalized by having the syn‐conformation of 8‐oxoG, however the G:I base pairing requires flipping of I toward its, least stable, syn‐conformational isomer renders a base pair that is thermodynamically less stable. Anti‐G:syn‐8oxoG have been reported to have some increased stability in duplexes of DNA, even more stability than an A:8oxoG base pair.^[^ ^57^ ^]^ We then carried out calculations on models containing the expected geometries, however we were surprised to find that an antiG:synG base pair did not lead to base pairs with a planar geometry (entries 44, 52). Interestingly the 8‐oxopurine:synG derivatives were found to be in the expected H‐bonding interactions. These results suggest that there are factors arising from the presence of the C8‐carbonyl that are contributing to the formation of a planar structure. Overall, the G:G base pair provides some thermal stability, compared to other base pairs, while not being able to form planarized structures, thus providing a degree of destabilization on the duplex.

*A & 8‐oxoA base pairing*. The trend of base pairing with A was in agreement with previous data, and 8‐oxoG displayed a similar behavior as U (as in the other cases shown herein). Interestingly, 8‐oxoA seems to have a promiscuous base pairing ability with the exception of its base pairing ability to C. This observation may be useful from a design perspective of RNA or drugs/small‐molecules mimicking this motif.

## 5 CONCLUSION

Overall, it is important to note that the model does not take into consideration stacking interactions and other conformational changes, thus limiting the amount of information that can be drawn from this data, which is in agreement with other models.^[^ ^58^ ^]^ However, it does provide an important picture in some cases and also yielded good evidence for the proposed base pairs. We established base pairing trends for all the possible combinations of the modifications, lesions, and canonical nucleobases described herein. The obtained data represents the first direct comparison on the thermal stabilization of duplexes containing the purine rings mentioned herein and could prove useful in biological and other applications.

## CONFLICT OF INTEREST

The authors declare no competing interests.

## Supporting information


**Appendix** **S1:** Supplementary Information

## Data Availability

All pertinent data, experimental and theoretical, is included within the provided supporting materials.
