# Supplementary material for: Experimental and theoretical rationalization for the base pairing abilities of inosine, guanosine, adenosine, and their corresponding 8‐oxo‐7,8‐dihydropurine, and 8‐bromopurine analogues within A‐form duplexes of RNA
Source: Biopolymers. 2020 Nov 20;111(12):e23410. doi: 10.1002/bip.23410 (PMC7780609; doi:10.1002/bip.23410)
Supplement: Supplementary file 1 — Appendix S1: Supplementary Information [file BIP-111-e23410-s001.zip › BIP_23410_SI-final.pdf]

Experimental and Theoretical Rationalization for the Base Pairing Abilities of Inosine, Guanosine, Adenosine, and their Corresponding 8-Oxo-7,8-dihydropurine, and 8-Bromopurine Analogues within A-form Duplexes of RNA.

Austin Skinner,† Chou-Hsun Yang,† Kazuki Hincks, Haobin Wang,* and Marino J. E. Resendiz*

Department of Chemistry, University of Colorado Denver, Science Building 1151 Arapahoe St, Denver, CO 80204, USA

* To whom correspondence should be addressed. Tel: 303-315-7658 ; Email: marino.resendiz@ucdenver.edu

† These authors contributed in the same amount to this work.

Supporting Information Index:

Page **Contents:**

S3-S5..................Figures S-1-3: MALDI TOF of oligonucleotides **6 - 14**.

S6.........................Figure S-4: MALDI TOF showing reactivity of oligonucleotide containing 8-BrA to the corresponding 8-methylamine adenine derivative

S7...…..................Figures S-5-6: Normalized UV-vis spectra of I, 8-oxogI, and 8-BrI at pH 7.1 and 8.2.

S8..…..................Figure S-7-8: Normalized UV-vis spectra of G, 8-oxoG, 8-BrG, A, 8-oxoA, and 8-BrA at pH 7.1.

S9-S10.................Figure S-9-14: CD and T_m measurement for duplex **1**:**2** - **1**:**7**

S11-S12...............Figure S-15-20: CD and T_m measurement for duplex **8**:**2** - **8**:**7**.

S13-S14...............Figure S-21-26: CD and T_m measurement for duplex **10**:**2** - **10**:**7**.

S15-S16...............Figure S-27-32: CD and T_m measurement for duplex **11**:**2** - **11**:**7**.

S17-S18...............Figure S-33-38: CD and T_m measurement for duplex **13**:**2** - **13**:**7**.

S19-S20...............Figure S-39-44: CD and T_m measurement for duplex **14**:**2** - **14**:**7**.

S21-S22...............Figure S-45-50: CD and T_m measurement for duplex **12**:**2** - **12**:**7**.

S23……………...Figure S-51: T_m measurement for duplexes **9**:**2** - **9**:**7**

S24.......................Tables S-1-3: T_m measurements carried out in triplicate for duplexes containing I, 8-oxoI, or G.

S25.......................Tables S-4-7: T_m measurements carried out in triplicate for duplexes containing 8-oxoG, A, 8-oxoA, 8-BrI

S26.......................Tables S-8: T_m measurements carried out in triplicate for duplexes containing 8-BrG

S26-27..................Tables S-9-10: Electronic structure theory calculation of free energies

S27-S79…………XYZ-files of base pairs of relevance to this work are available as follows:

S27………antiG:antiC, entry1
S28………antiI:antiC, entry2
S29………anti8oxoG:antiC, entry3
S30………anti8BrG:antiC, entry4
S31………anti8oxoI:antiC, entry5
S32………anti8BrI:antiC, entry6
S33………synG:antiC, entry7
S34………synI:antiC, entry8
S35………syn8oxoG:antiC, entry9
S36………synG:synC, entry10
S37………synI:synC, entry11
S38………antiG:antiU, entry12
S39………antiI:antiU, entry13
S40………anti8oxoG:antiU, entry14
S41………anti8BrG:antiU, entry15
S42………anti8oxoI:antiU, entry16
S43………anti8BrI:antiU, entry17
S44………synG:antiU, entry18
S45………synI:antiU, entry19
S46………syn8oxoG:antiU, entry20
S47………syn8oxoI:antiU, entry21
S48………antiG:antiA, entry22
S49………antiI:antiA, entry23
S50………anti8oxoG:antiA, entry24
S51………anti8oxoI:antiA, entry25
S52………anti8BrG:antiA, entry26
S53………anti8BrI:antiA, entry27
S54………synG:antiA, entry28
S55………synI:antiA, entry29
S56………syn8oxoG:antiA, entry30
S57………syn8oxoI:antiA, entry31
S58………syn8BrG:antiA, entry32
S59………syn8-BrI:antiA, entry33
S60………antiG:synA, entry34
S61………antiI:synA, entry35
S62………anti8oxoG:synA, entry36
S63………anti8oxoI:synA, entry37
S64………anti8BrG:synA, entry38
S65………anti8BrI:synA, entry39
S66………synG:synA, entry40
S67………syn8oxoG:synA, entry41
S68………syn8oxoI:synA, entry42
S69………syn8BrG:synA, entry43
S70………antiG:synG, entry44
S71………antiI:synG, entry45
S72………antiI:synG, entry46
S73………anti8oxoG:synG, entry47
S74………anti8oxoG:synG #2, entry48
S75………anti8oxoI:synG, entry49
S76………anti8BrG:synG, entry50
S77………syn8oxoG:synG, entry51
S78………synI:antiG, entry52
S79………syn8BrI:antiG, entry53
S79………Reference

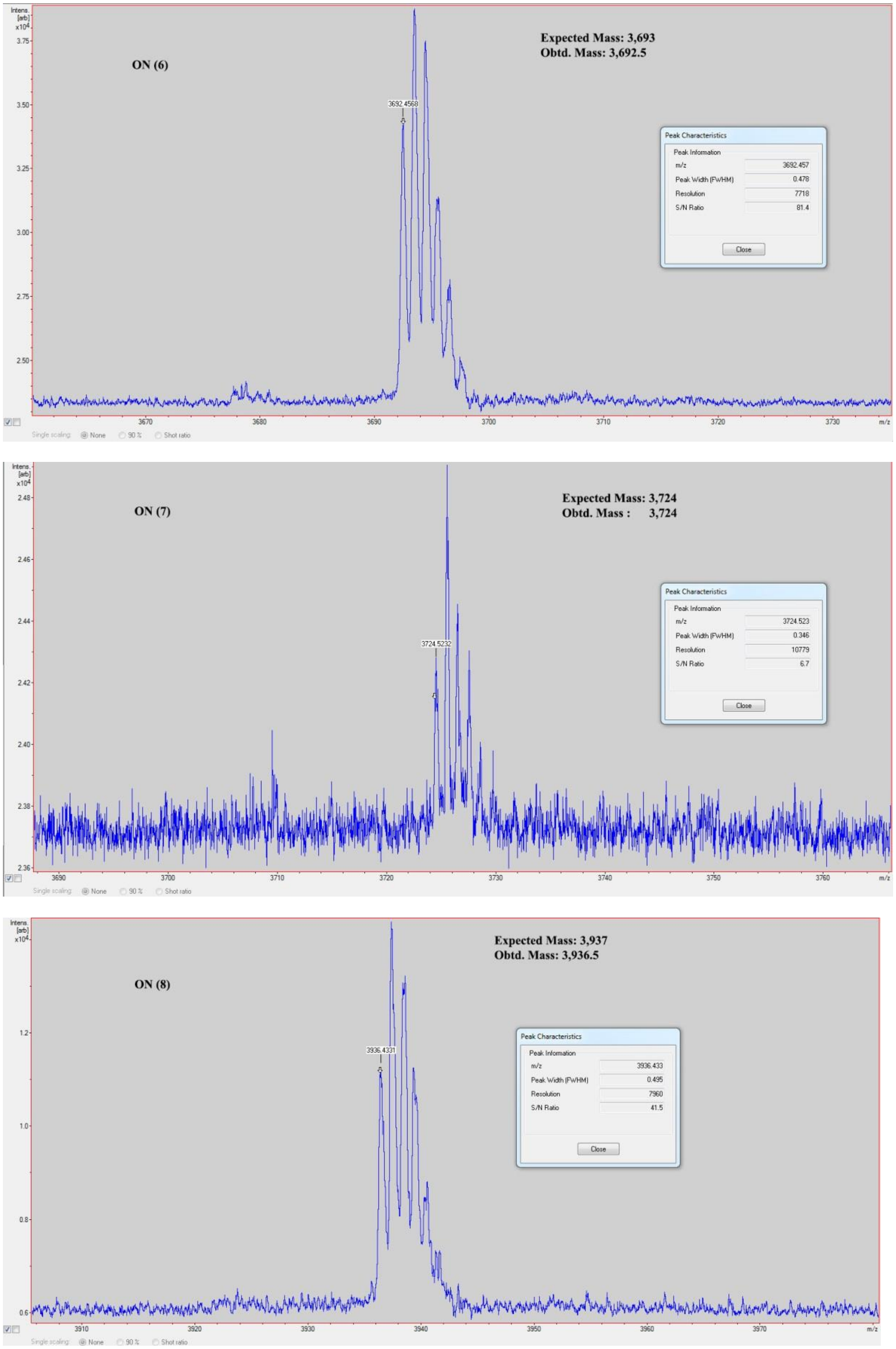

Figure SI-1. MALDI TOF of oligonucleotides **6 - 8**.

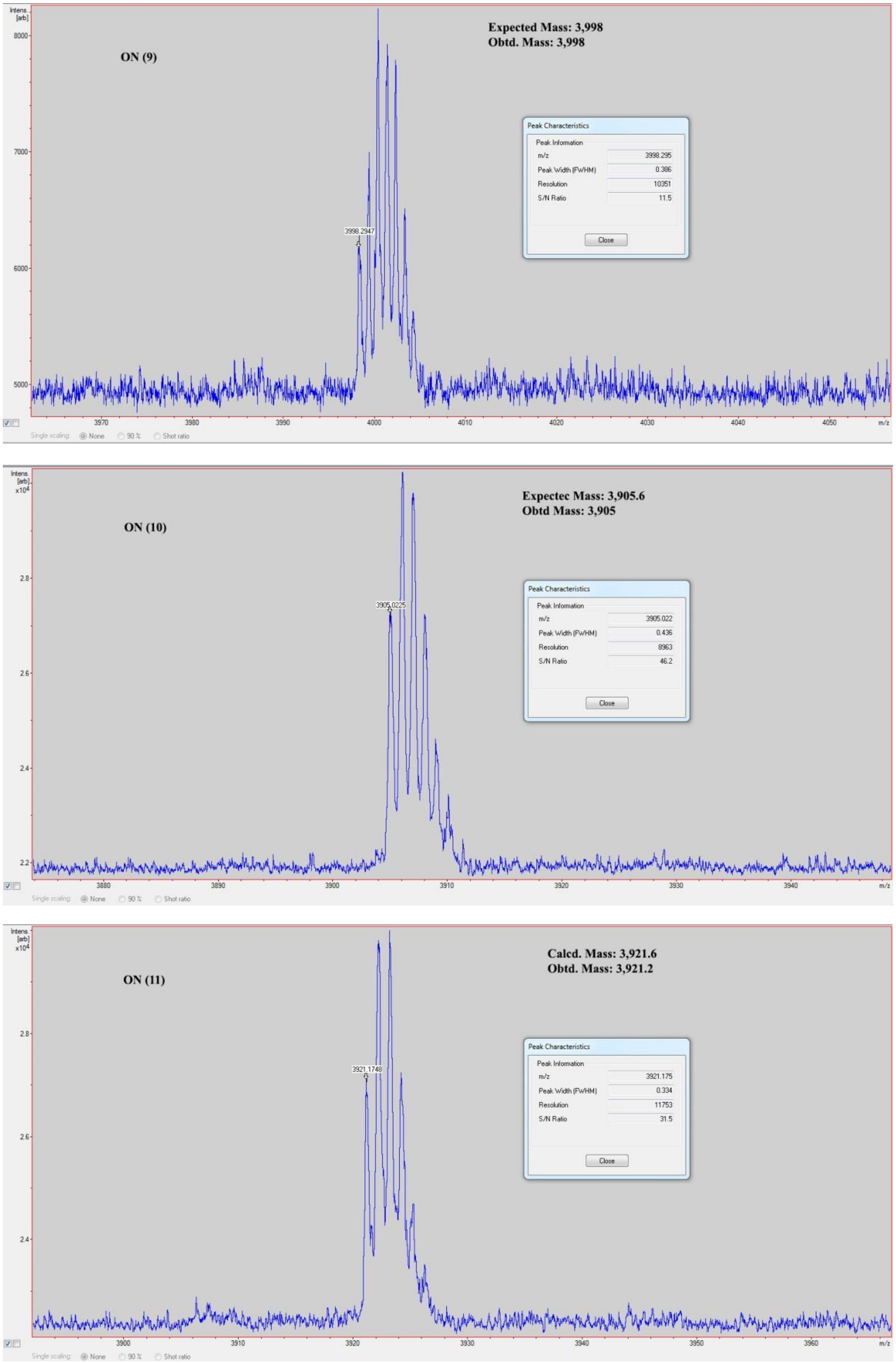

Figure SI-2. MALDI TOF of oligonucleotides **9 - 11**.

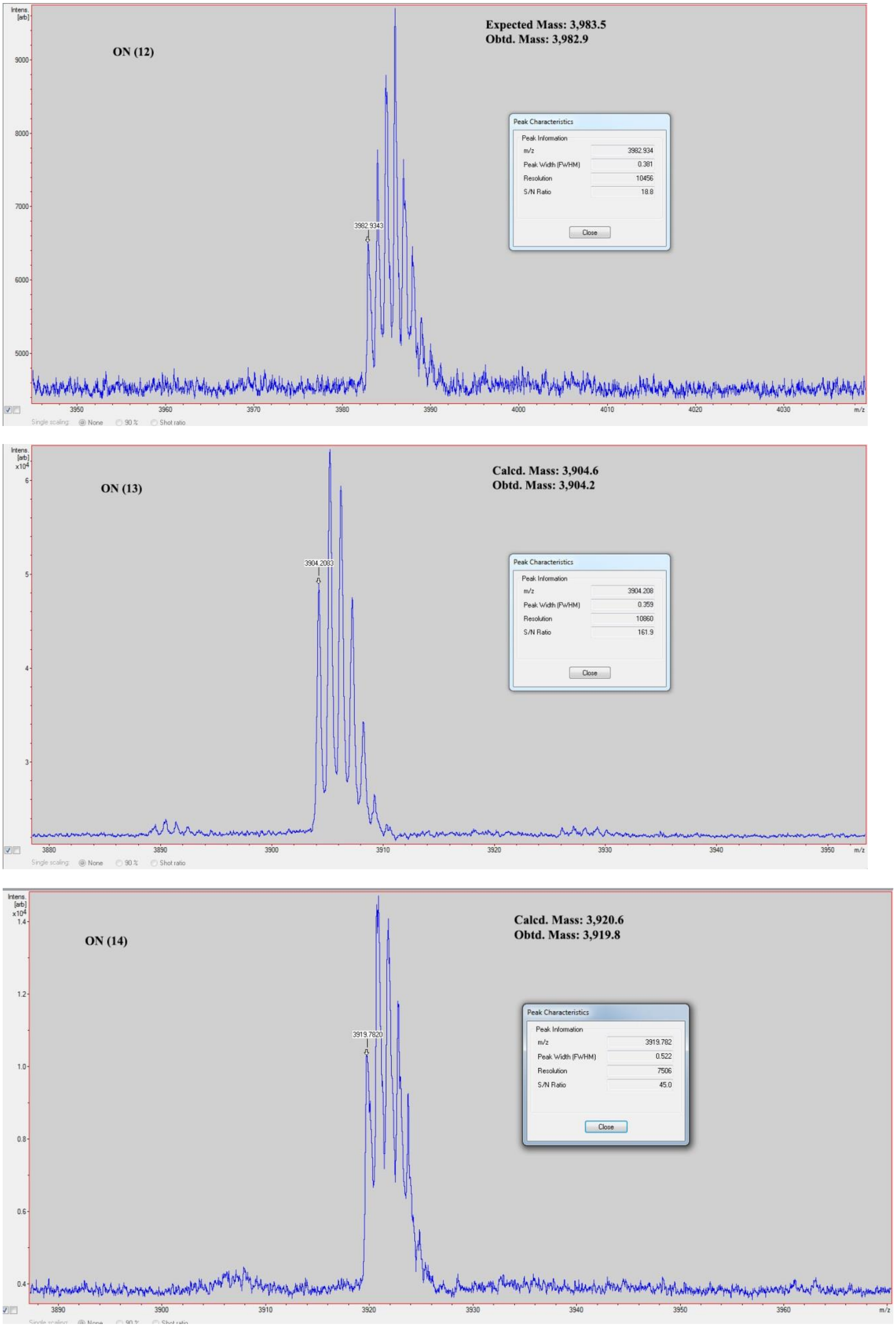

Figure SI-3. MALDI TOF of oligonucleotides **12** - **14**.

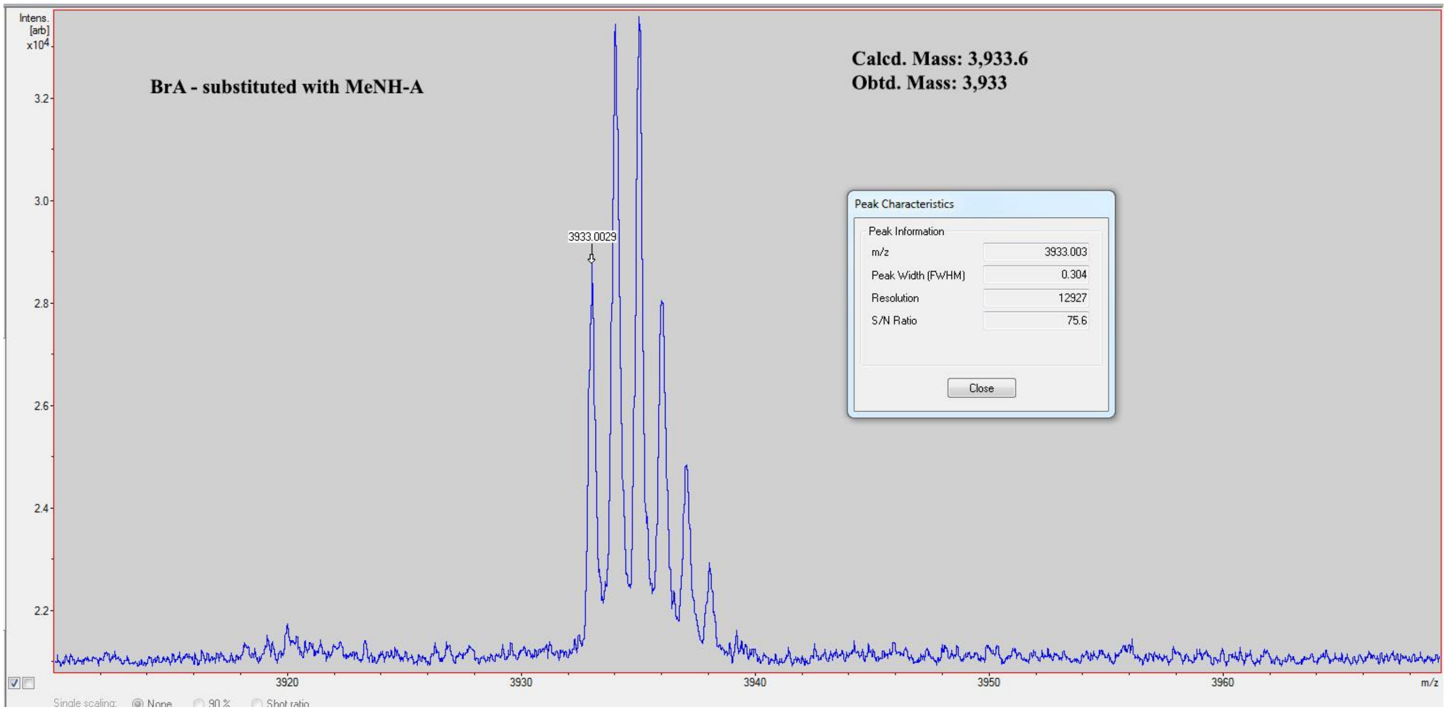

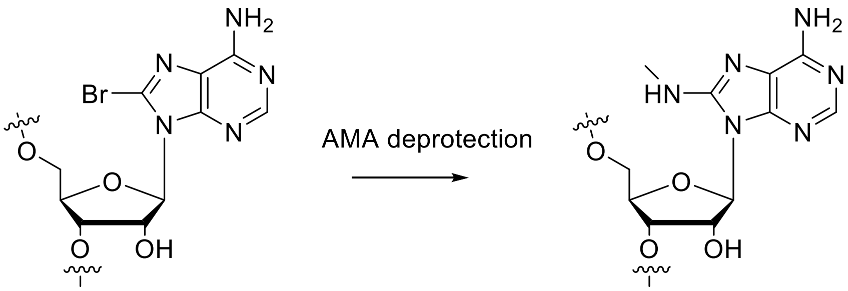

Figure SI-4. MALDI TOF of oligonucleotides 8-BrA substituted with methyl amine.

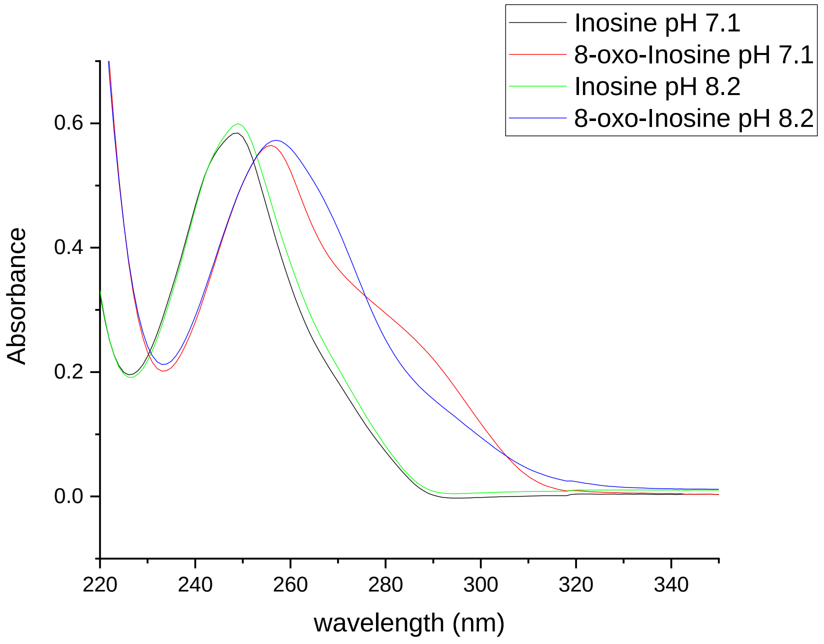

Figure SI-5: Normalized UV-Vis spectral overlay of inosine and 8-oxo-inosine (50 µM) in pH 7.1 and 8.2 phosphate buffer

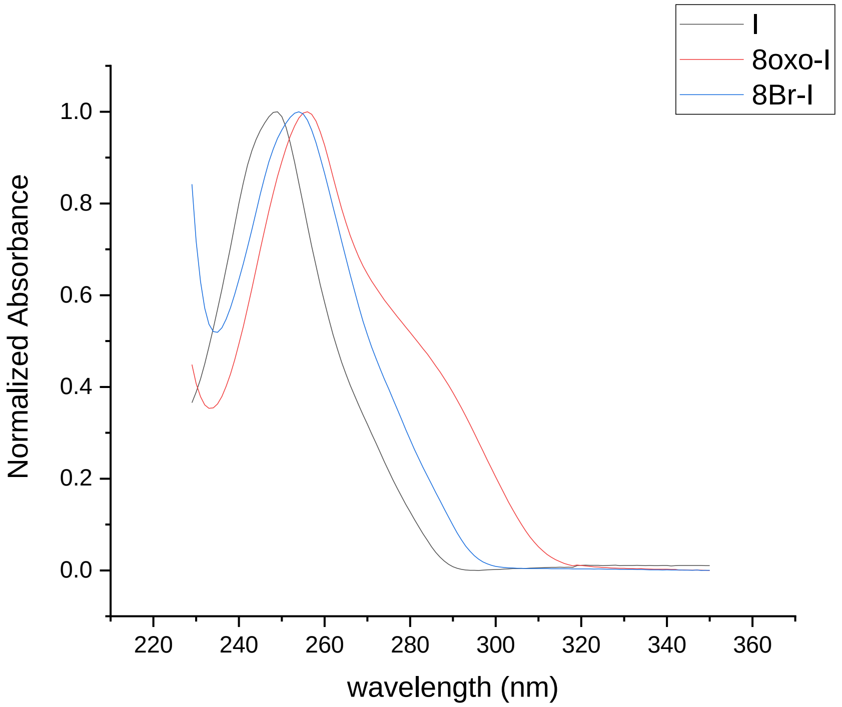

Figure SI-6: Normalized UV-Vis spectra overlay of inosine, 8-oxo-inosine, and 8-bromo-inosine monomers (50 µM) in phosphate buffer, pH 7.1.

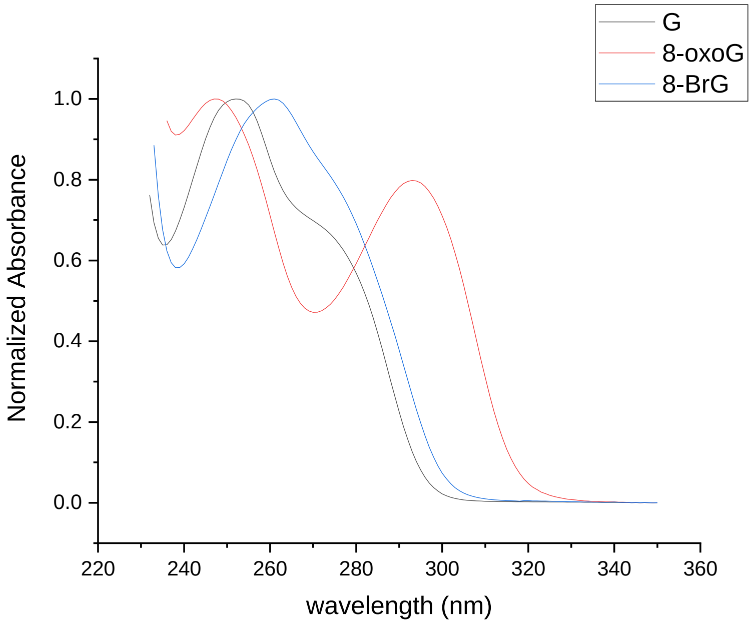

Figure SI-7: UV-Vis spectral overlay of guanosine, 8-oxo-guanosine, and 8-bromo-guanosine monomers (50 µM) in phosphate buffer, pH 7.1.

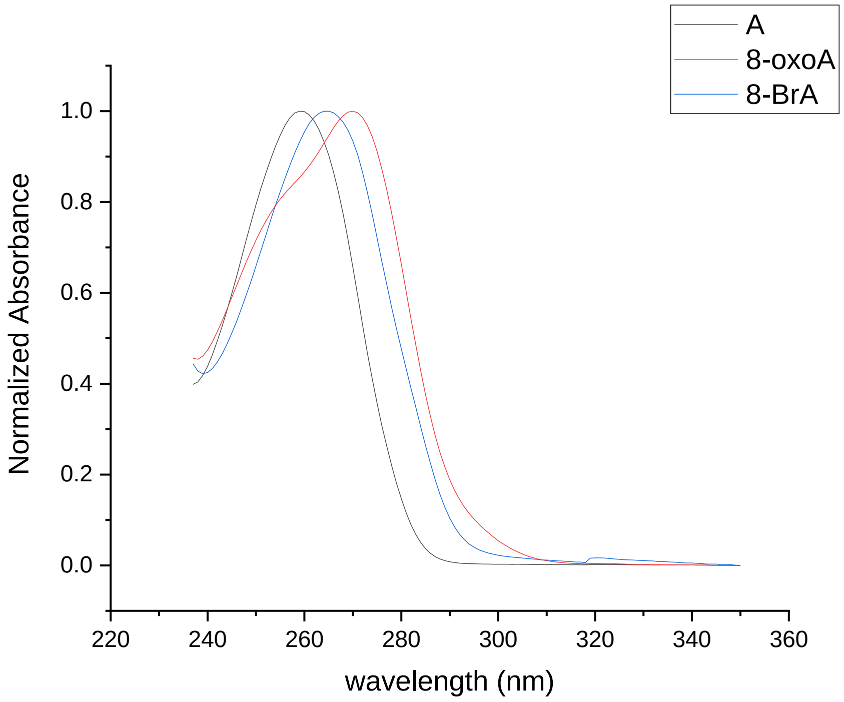

Figure SI-8: UV-Vis spectral overlay of adenosine, 8-oxo-adenosine, and 8-bromo-adenosine monomers (50 µM) in phosphate buffer, pH 7.1.

Experimental conditions: 2.5 µM RNA and 3.0 µM complement RNA prepared in PBS (10 mM NaCl, 5 mM $MgCl_2$, 1 mM Na_2HPO_4 pH 7.2)

5’-AAG-AG**G**-GAU-GAC-3’
3’-UUC-UC**X**-CUA-CUG-5’

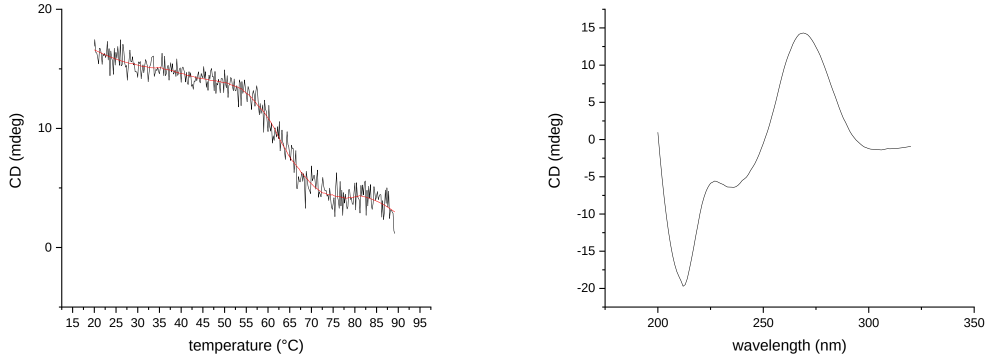

Figure SI-9: X = 8-oxoG; Duplex **1:7** CD data recorded at 270 nm (left) and full CD spectrum of duplex (right)

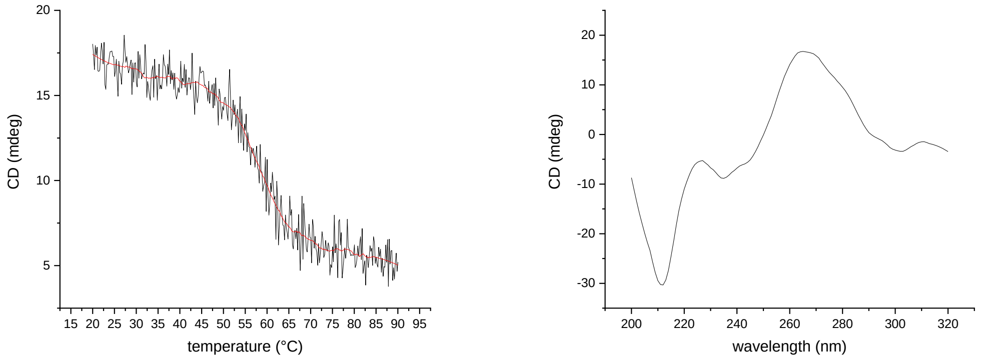

Figure SI-10: X = A; Duplex **1:4** CD data recorded at 270 nm (left) and full CD spectrum of duplex (right)

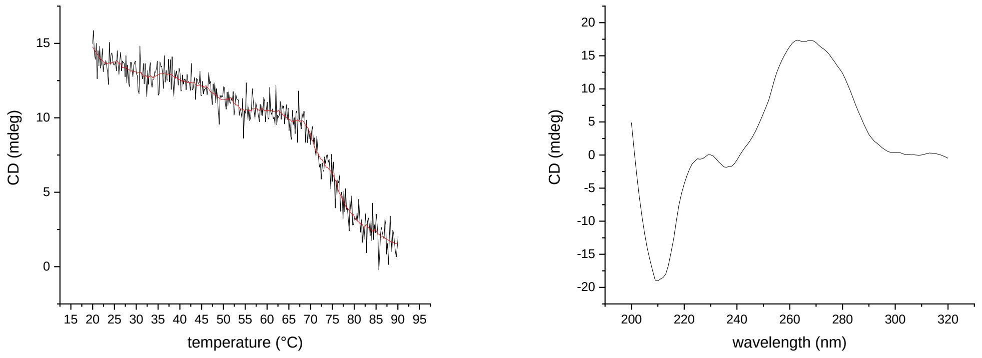

Figure SI-11: X = C; Duplex **1:5** CD data recorded at 270 nm (left) and full CD spectrum of duplex (right)

Experimental conditions: 2.5 µM RNA and 3.0 µM complement RNA prepared in PBS (10 mM NaCl, 5 mM $MgCl_2$, 1 mM Na_2HPO_4 pH 7.2)

5'-AAG-AG**G**-GAU-GAC-3'
3'-UUC-UC**X**-CUA-CUG-5'

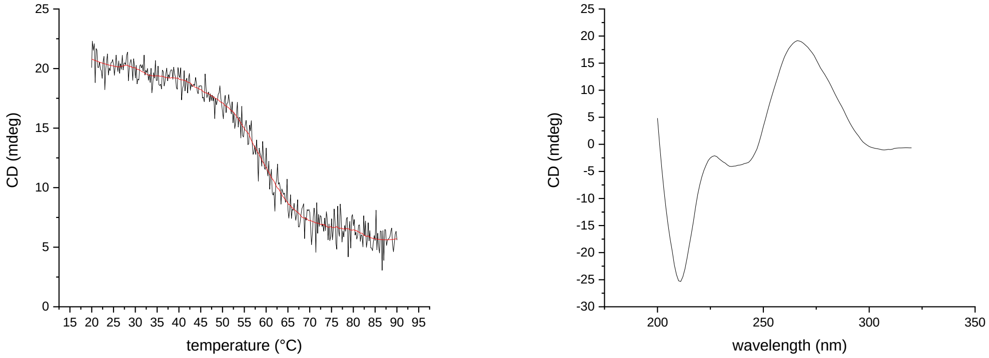

Figure SI-12. X = G; Duplex **1:2** CD data recorded at 270 nm (left) and full CD spectrum of duplex (right)

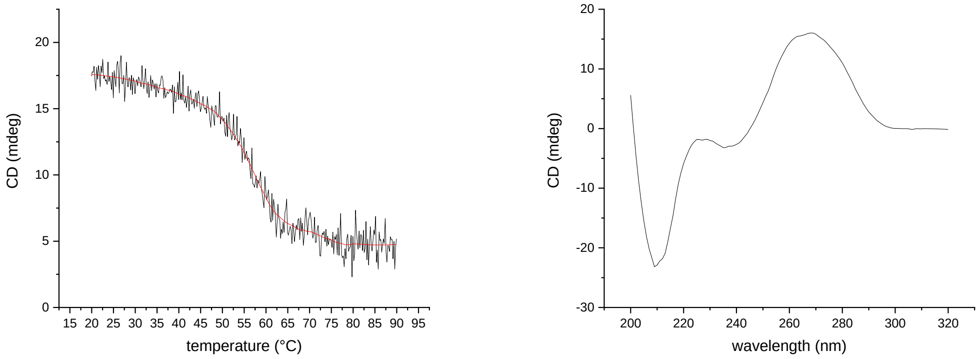

Figure SI-13: X = I; Duplex **1:6** CD data recorded at 270 nm (left) and full CD spectrum of duplex (right)

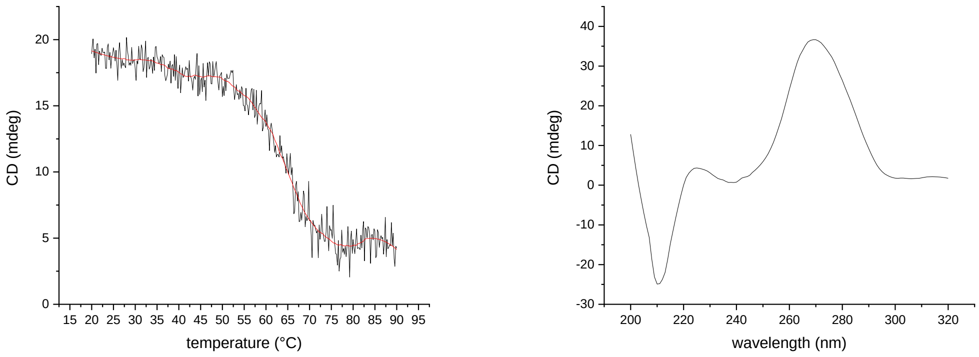

Figure SI-14: X = U; Duplex **1:3** CD data recorded at 270 nm (left) and full CD spectrum of duplex (right)

Experimental conditions: 2.5 µM RNA and 3.0 µM complement RNA prepared in PBS (10 mM NaCl, 5 mM $MgCl_2$, 1 mM Na_2HPO_4 pH 7.2)

5’-AAG-AG**G**^{oxo}-GAU-GAC-3’
3’-UUC-UC**X**-CUA-CUG-5

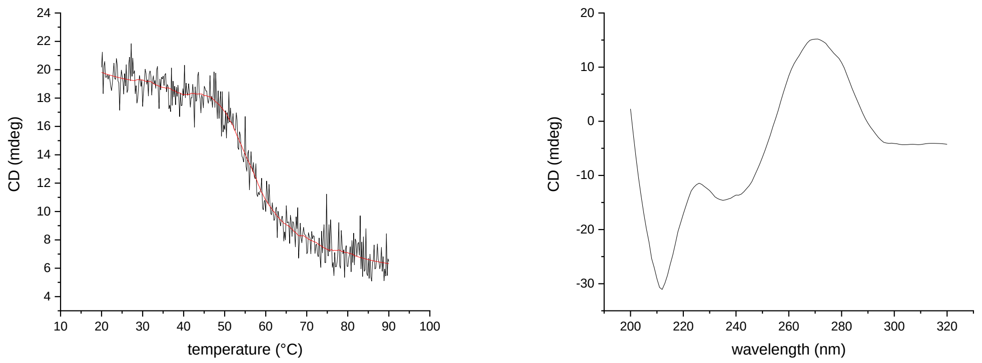

Figure SI-15: X = 8-oxoG; Duplex **8:7** CD data recorded at 270 nm (left) and full CD spectrum of duplex (right)

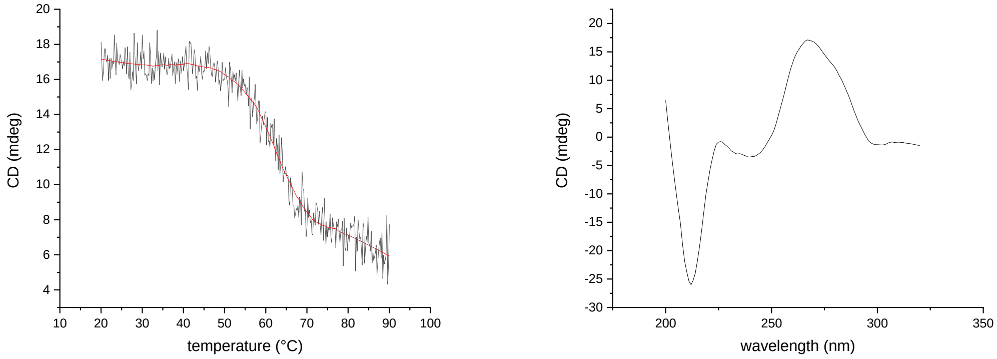

Figure SI-16: X = A; Duplex **8:4** CD data recorded at 270 nm (left) and full CD spectrum of duplex (right)

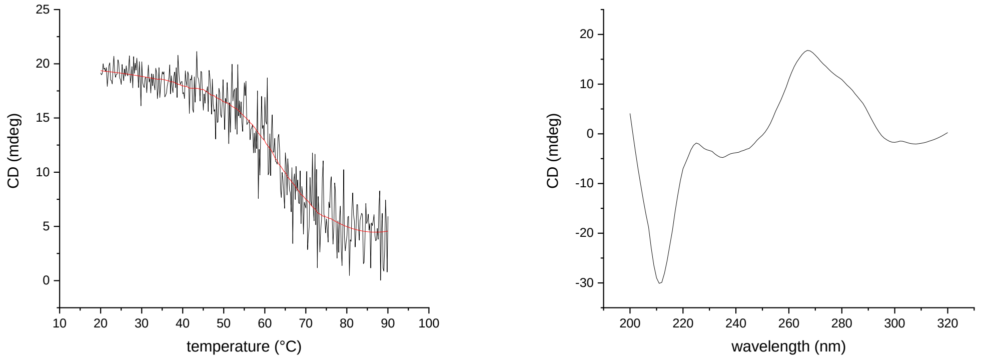

Figure SI-17: X = C; Duplex **8:5** CD data recorded at 270 nm (left) and full CD spectrum of duplex (right)

Experimental conditions: 2.5 µM RNA and 3.0 µM complement RNA prepared in PBS (10 mM NaCl, 5 mM $MgCl_2$, 1 mM Na_2HPO_4 pH 7.2)

5’-AAG-AG**G**^{oxo}-GAU-GAC-3’
3’-UUC-UC**X**-CUA-CUG-5

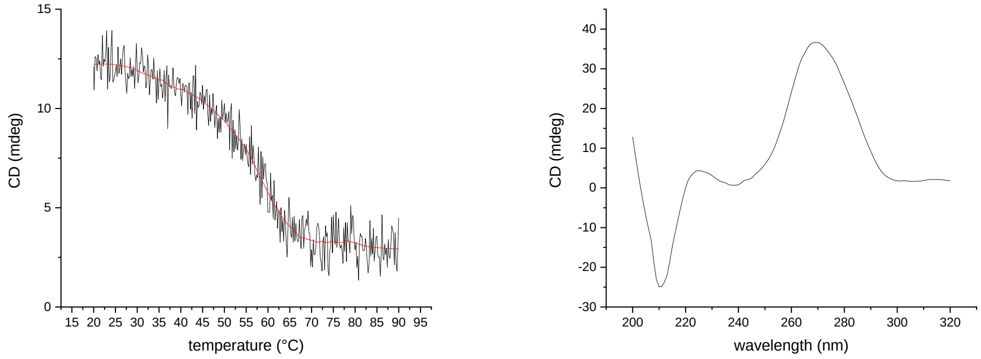

Figure SI-18: X = G; Duplex **8:2** CD data recorded at 270 nm (left) and full CD spectrum of duplex (right)

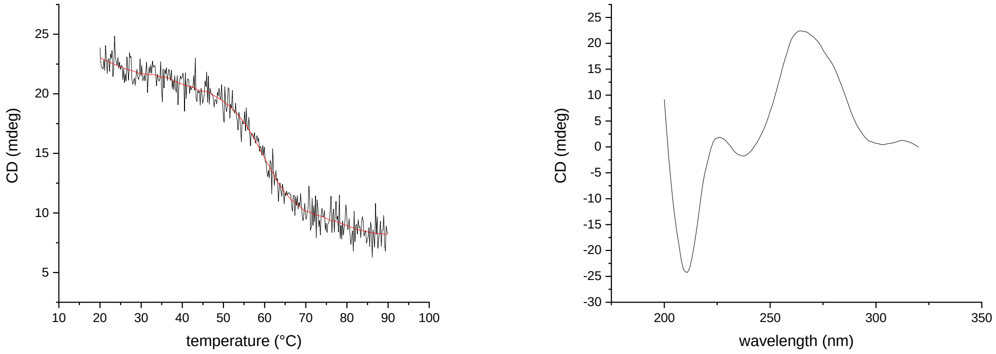

Figure SI-19: X = I; Duplex **8:6** CD data recorded at 270 nm (left) and full CD spectrum of duplex (right)

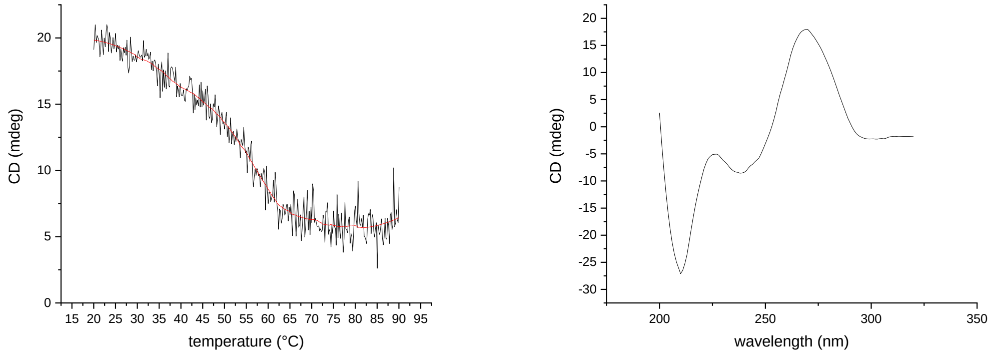

Figure SI-20: X = U; Duplex **8:3** CD data recorded at 270 nm (left) and full CD spectrum of duplex (right)

Experimental conditions: 2.5 µM RNA and 3.0 µM complement RNA prepared in PBS (10 mM NaCl, 5 mM $MgCl_2$, 1 mM Na_2HPO_4 pH 7.2)

5'-AAG-AG**I**-GAU-GAC-3'
3'-UUC-UC**X**-CUA-CUG-5'

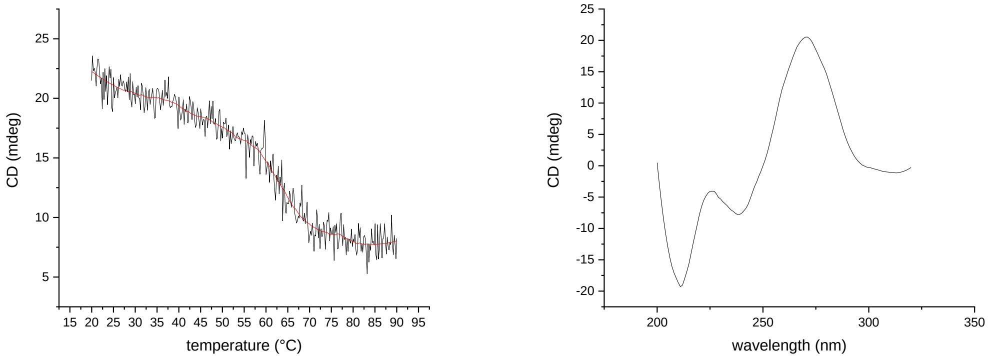

Figure SI-21: X = 8-oxoG; Duplex **10:7** CD data recorded at 270 nm (left) and full CD spectrum of duplex (right)

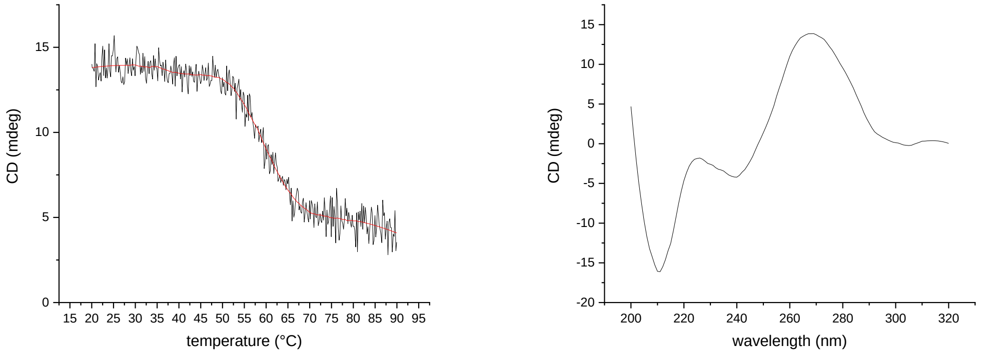

Figure SI-22: X = A; Duplex **10:4** CD data recorded at 270 nm (left) and full CD spectrum of duplex (right)

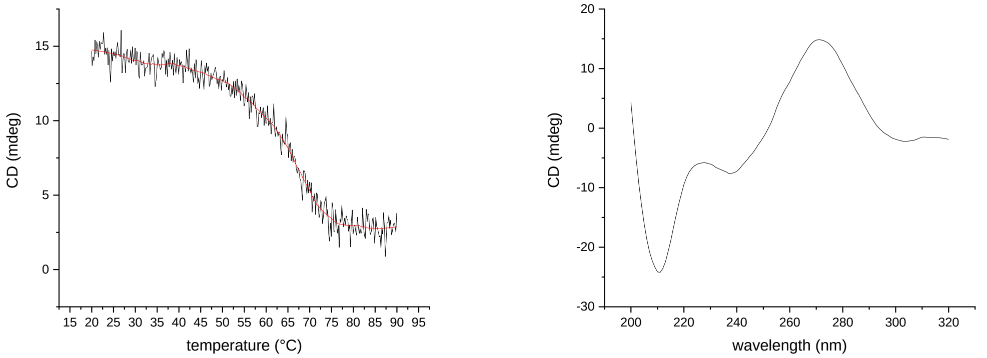

Figure SI-23: X = C; Duplex **10:5** CD data recorded at 270 nm (left) and full CD spectrum of duplex (right)

Experimental conditions: 2.5 µM RNA and 3.0 µM complement RNA prepared in PBS (10 mM NaCl, 5 mM $MgCl_2$, 1 mM Na_2HPO_4 pH 7.2)

5’-AAG-AG**I**-GAU-GAC-3’
3’-UUC-UC**X**-CUA-CUG-5’

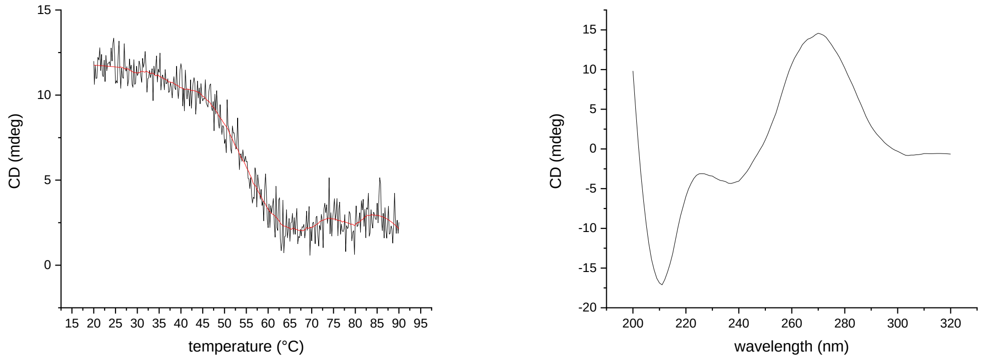

Figure SI-24. X = G; Duplex **10:2** CD data recorded at 270 nm (left) and full CD spectrum of duplex (right)

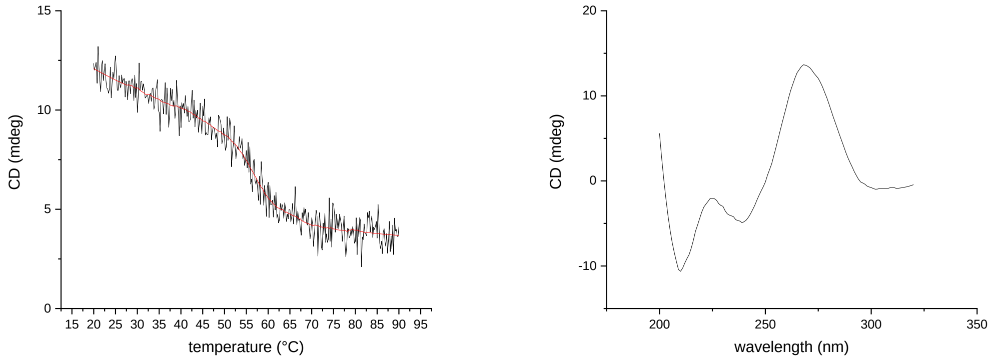

Figure SI-25. X = I; Duplex **10:6** CD data recorded at 270 nm (left) and full CD spectrum of duplex (right)

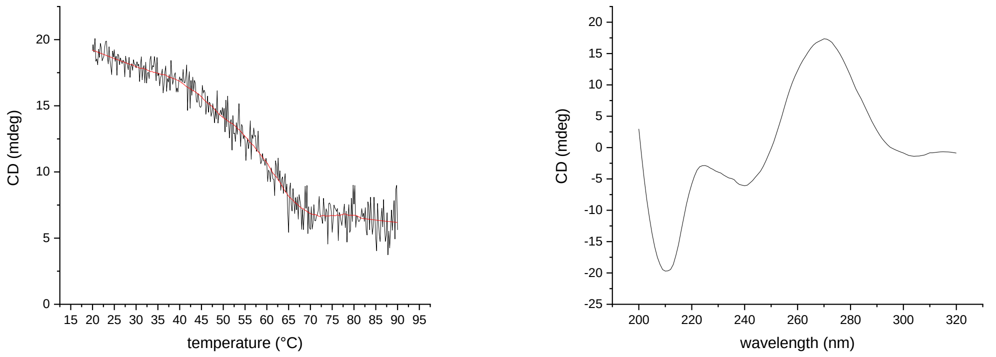

Figure SI-26: X = U; Duplex **10:3** CD data recorded at 270 nm (left) and full CD spectrum of duplex (right)

Experimental conditions: 2.5 µM RNA and 3.0 µM complement RNA prepared in PBS (10 mM NaCl, 5 mM $MgCl_2$, 1 mM Na_2HPO_4 pH 7.2)

5'-AAG-AG**I**$^{\mathbf{oxo}}$-GAU-GAC-3'
3'-UUC-UC**X**-CUA-CUG-5'

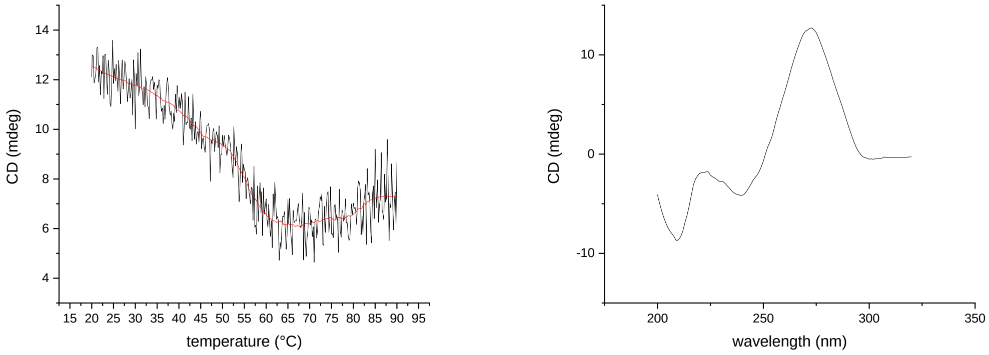

Figure SI-27: X = 8-oxoG Duplex **11:7** CD data recorded at 270 nm (left) and full CD spectrum of duplex (right)

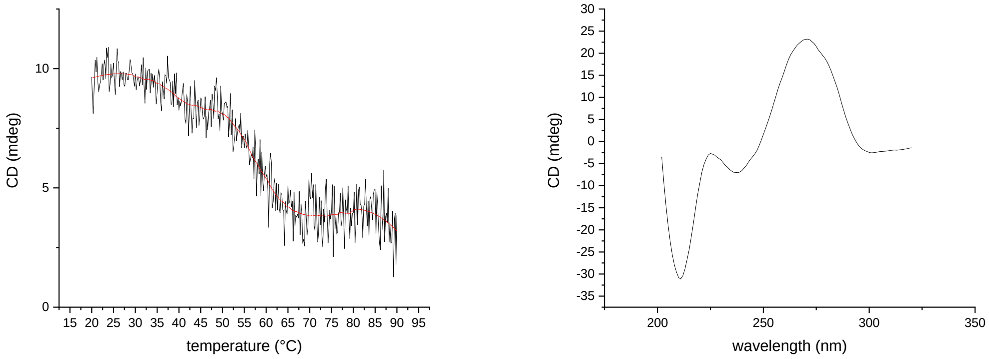

Figure SI-28: X = A; Duplex **11:4** CD data recorded at 270 nm (left) and full CD spectrum of duplex (right)

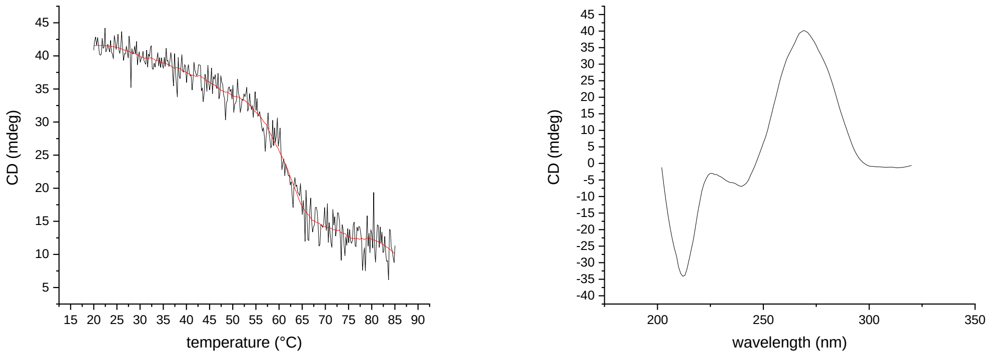

Figure SI-29: X = C; Duplex **11:5** CD data recorded at 270 nm (left) and full CD spectrum of duplex (right)

Experimental conditions: 2.5 µM RNA and 3.0 µM complement RNA prepared in PBS (10 mM NaCl, 5 mM $MgCl_2$, 1 mM Na_2HPO_4 pH 7.2)

5’-AAG-AG**I**^{oxo}-GAU-GAC-3’
3’-UUC-UC**X**-CUA-CUG-5’

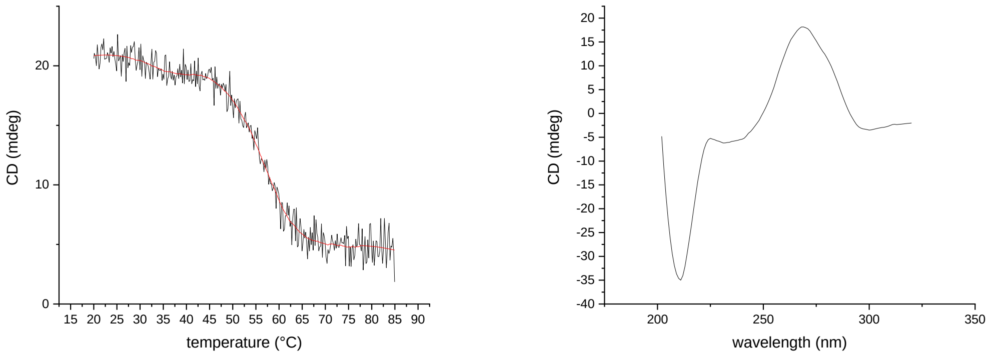

Figure SI-30: X = G; Duplex CD **11:2** data recorded at 270 nm (left) and full CD spectrum of duplex (right)

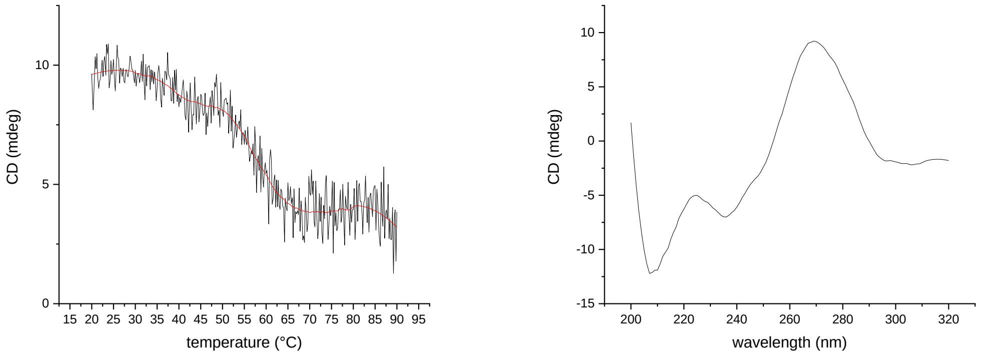

Figure SI-31: X = I; Duplex **11:6** CD data recorded at 270 nm (left) and full CD spectrum of duplex (right)

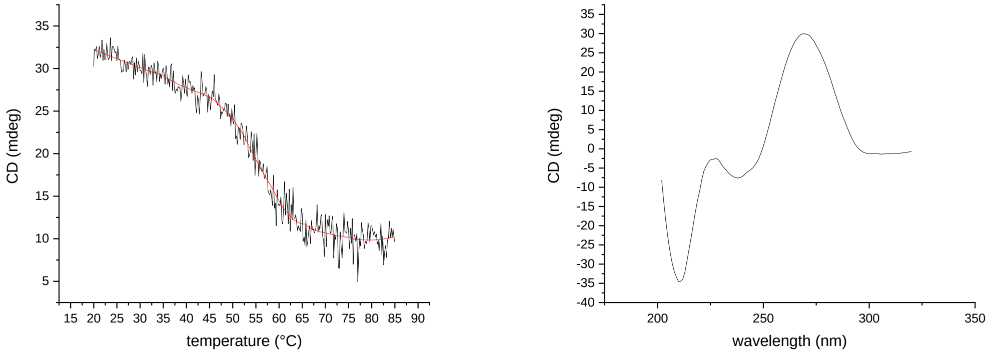

Figure SI-32: X = U; Duplex **11:3** CD data recorded at 270 nm (left) and full CD spectrum of duplex (right)

Experimental conditions: 2.5 µM RNA and 3.0 µM complement RNA prepared in PBS (10 mM NaCl, 5 mM $MgCl_2$, 1 mM Na_2HPO_4 pH 7.2)

5’-AAG-AG**A**-GAU-GAC-3’
3’-UUC-UC**X**-CUA-CUG-5’

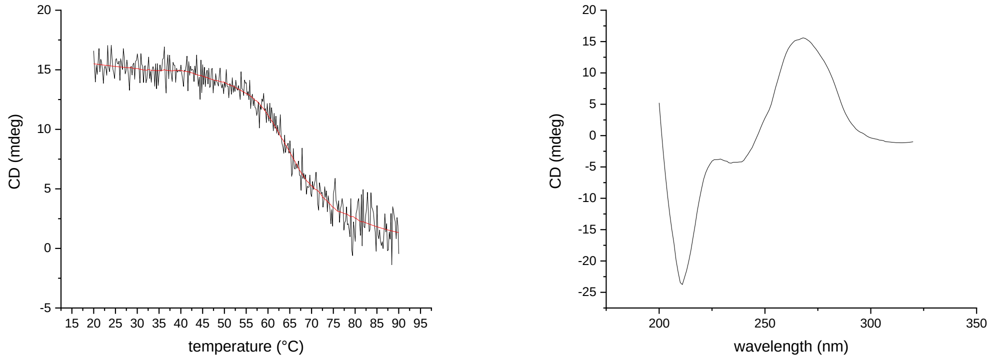

Figure SI-33. X = 8-oxoG; Duplex **13:7** CD data recorded at 270 nm (left) and full CD spectrum of duplex (right)

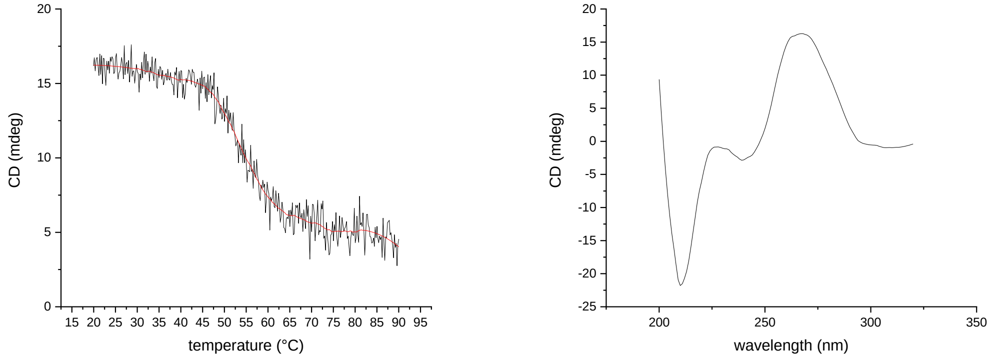

Figure SI-34. X = A; Duplex **13:4** CD data recorded at 270 nm (left) and full CD spectrum of duplex (right)

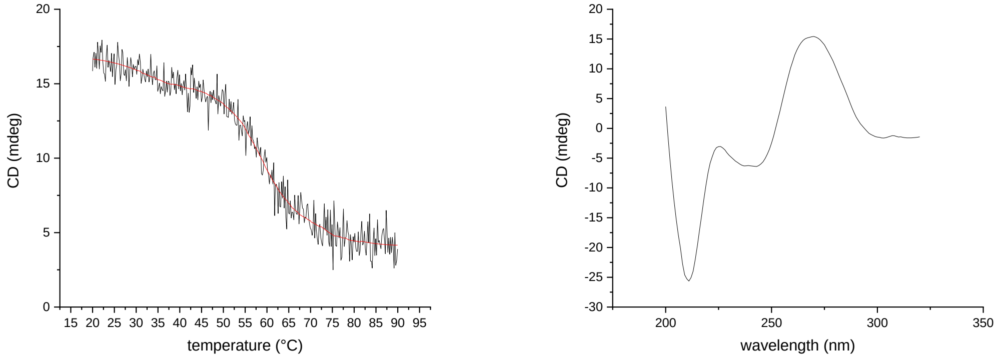

Figure SI-35. X = C; Duplex **13:5** CD data recorded at 270 nm (left) and full CD spectrum of duplex (right)

Experimental conditions: 2.5 µM RNA and 3.0 µM complement RNA prepared in PBS (10 mM NaCl, 5 mM $MgCl_2$, 1 mM Na_2HPO_4 pH 7.2)

5’-AAG-AG**A**-GAU-GAC-3’
3’-UUC-UC**X**-CUA-CUG-5’

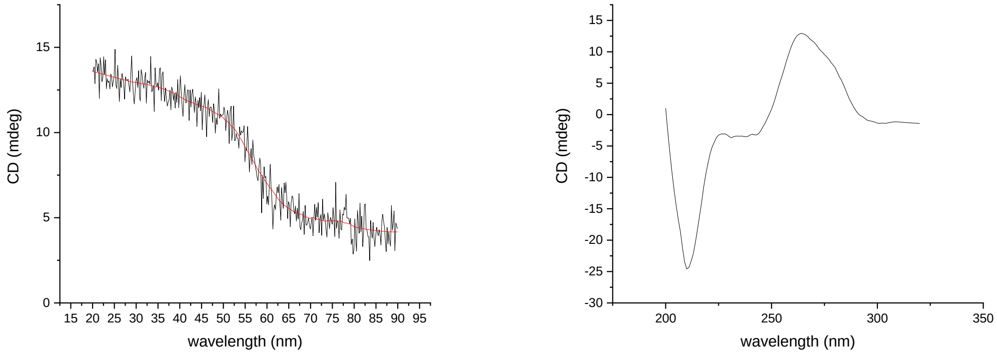

Figure SI-36: X = C; Duplex **13:5** CD data recorded at 270 nm (left) and full CD spectrum of duplex (right)

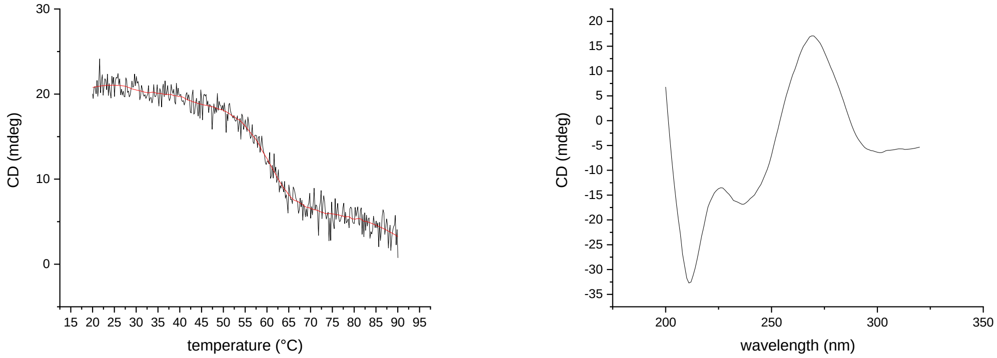

Figure SI-37. X = I; Duplex **13:6** CD data recorded at 270 nm (left) and full CD spectrum of duplex (right)

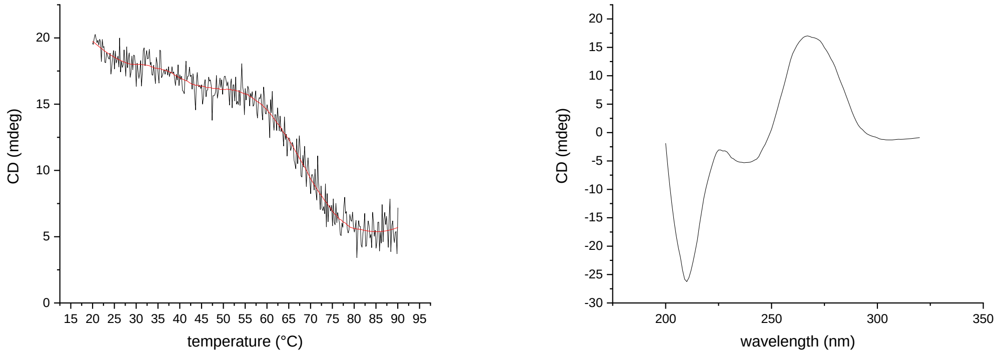

Figure SI-38. X = U; Duplex **13:3** CD data recorded at 270 nm (left) and full CD spectrum of duplex (right)

Experimental conditions: 2.5 µM RNA and 3.0 µM complement RNA prepared in PBS (10 mM NaCl, 5 mM $MgCl_2$, 1 mM Na_2HPO_4 pH 7.2)

5’-AAG-AG**A**^{oxo}-GAU-GAC-3’
3’-UUC-UC**X**-CUA-CUG-5’

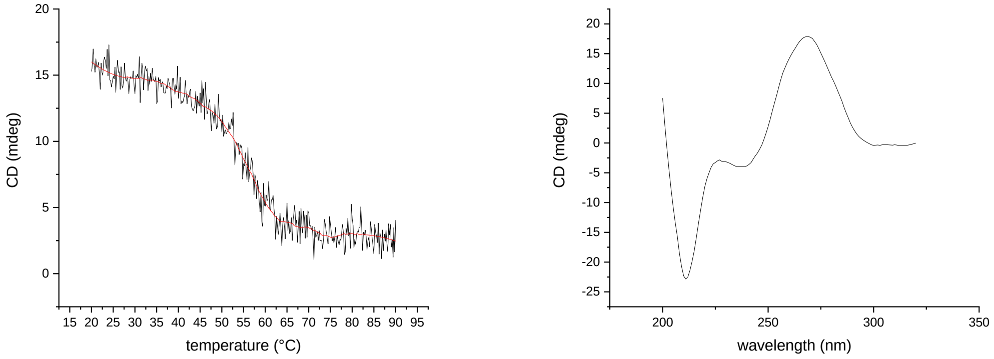

Figure SI-39. X = 8-oxoG; Duplex **14:7** CD data recorded at 270 nm (left) and full CD spectrum of duplex (right)

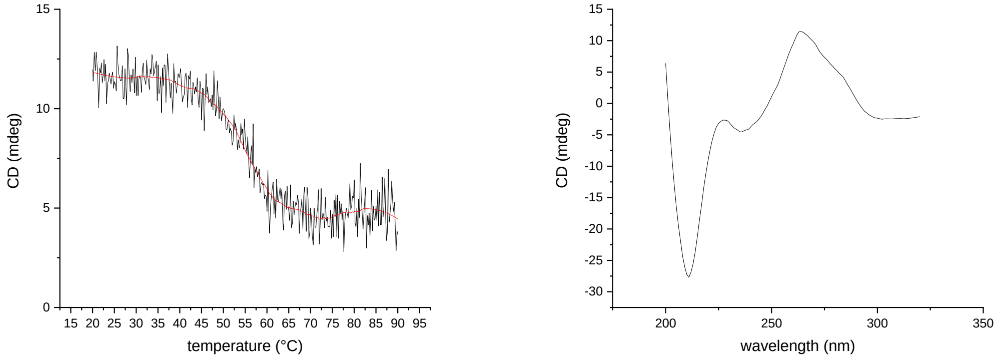

Figure SI-40. X = A; Duplex **14:4** CD data recorded at 270 nm (left) and full CD spectrum of duplex (right)

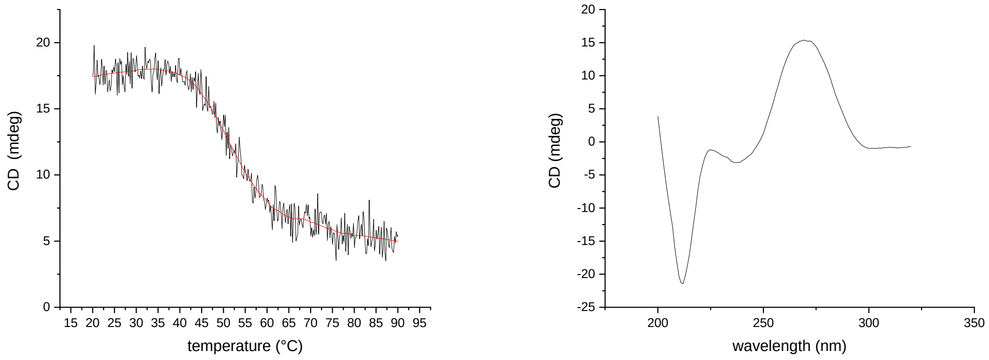

Figure SI-41. X = C; Duplex **14:5** CD data recorded at 270 nm (left) and full CD spectrum of duplex (right)

Experimental conditions: 2.5 µM RNA and 3.0 µM complement RNA prepared in PBS (10 mM NaCl, 5 mM $MgCl_2$, 1 mM Na_2HPO_4 pH 7.2)

5’-AAG-AG**A**^{oxo}-GAU-GAC-3’
3’-UUC-UC**X**-CUA-CUG-5’

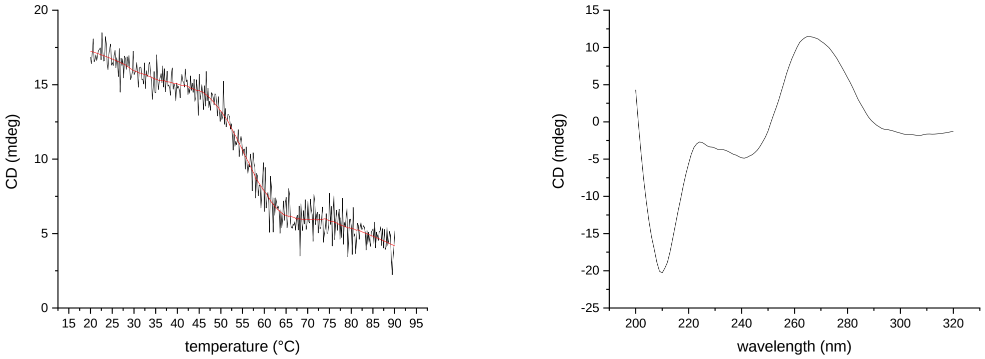

Figure SI-42. X = G; Duplex **14:2** CD data recorded at 270 nm (left) and full CD spectrum of duplex (right)

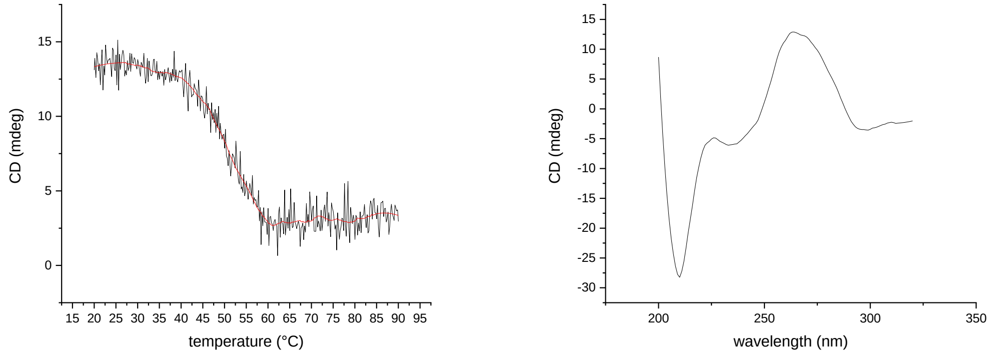

Figure SI-43. X = I; Duplex **14:6** CD data recorded at 270 nm (left) and full CD spectrum of duplex (right)

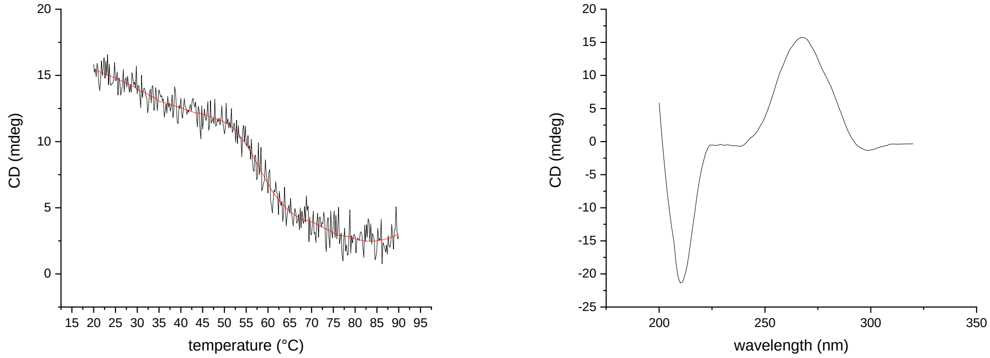

Figure SI-44. X = U; Duplex **14:3** CD data recorded at 270 nm (left) and full CD spectrum of duplex (right)

Experimental conditions: 2.5 µM RNA and 3.0 µM complement RNA prepared in PBS (10 mM NaCl, 5 mM $MgCl_2$, 1 mM Na_2HPO_4 pH 7.2)

5’-AAG-AG**I**$^{\mathbf{Br}}$-GAU-GAC-3’
3’-UUC-UC**X**-CUA-CUG-5’

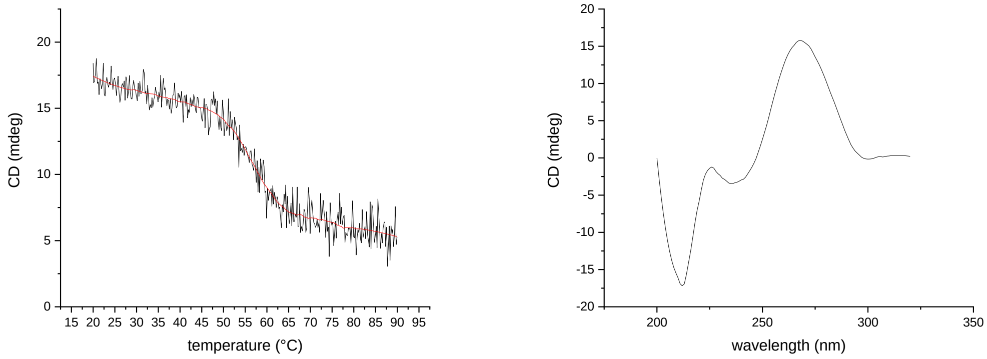

Figure SI-45. X = 8-oxoG; Duplex **12:7** CD data recorded at 270 nm (left) and full CD spectrum of duplex (right)

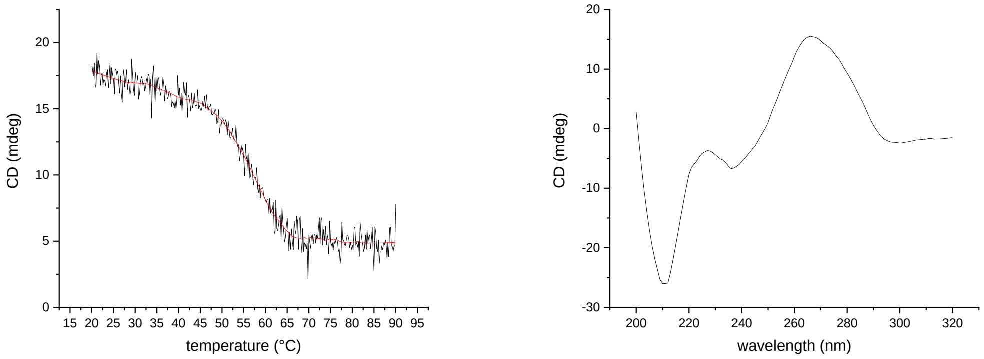

Figure SI-46. X = A; Duplex **12:4** CD data recorded at 270 nm (left) and full CD spectrum of duplex (right)

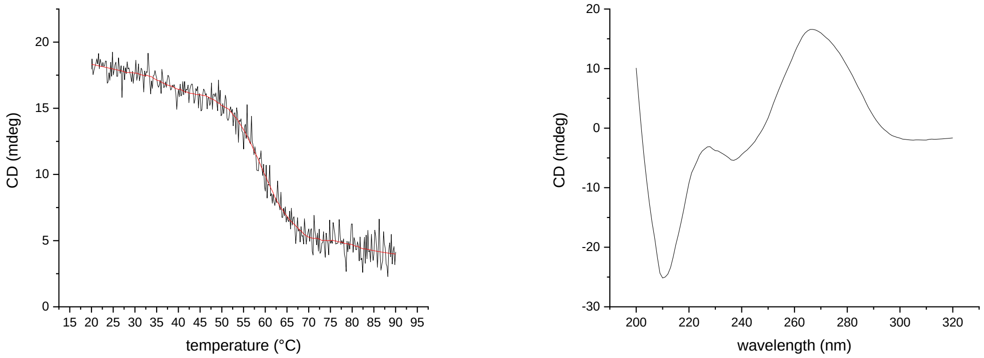

Figure SI-47. X = C; Duplex **12:5** CD data recorded at 270 nm (left) and full CD spectrum of duplex (right)

Experimental conditions: 2.5 µM RNA and 3.0 µM complement RNA prepared in PBS (10 mM NaCl, 5 mM $MgCl_2$, 1 mM Na_2HPO_4 pH 7.2)

5'-AAG-AG$\mathbf{I}^{\mathbf{Br}}$-GAU-GAC-3'
3'-UUC-UC**X**-CUA-CUG-5'

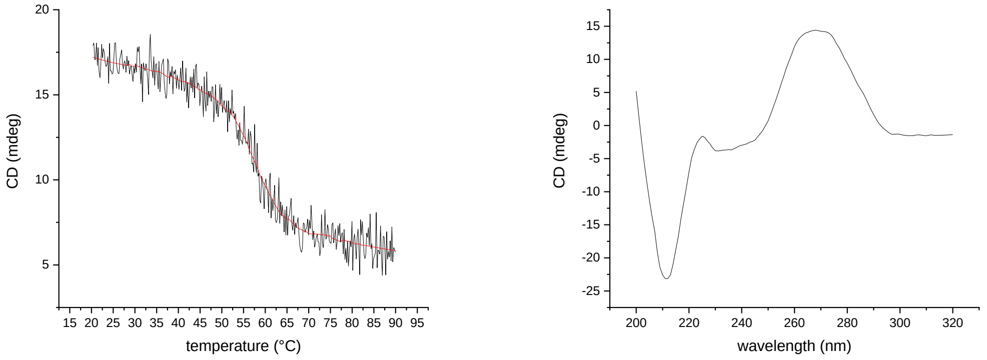

Figure SI-48. X = G; Duplex **12:2** CD data recorded at 270 nm (left) and full CD spectrum of duplex (right)

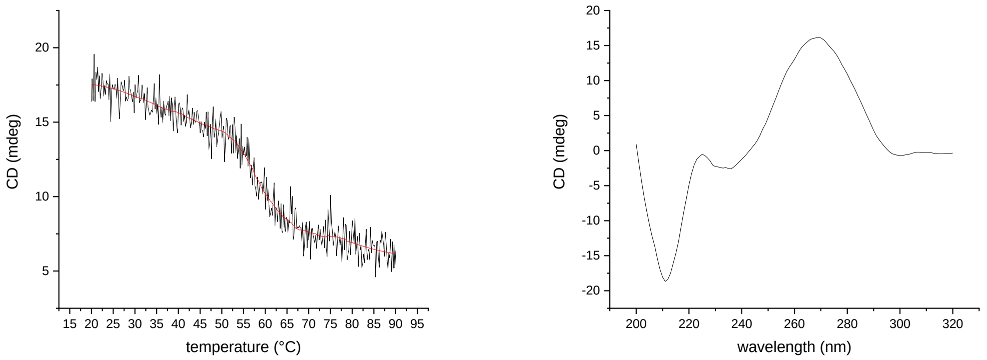

Figure SI-49. X = I; Duplex **12:6** CD data recorded at 270 nm (left) and full CD spectrum of duplex (right)

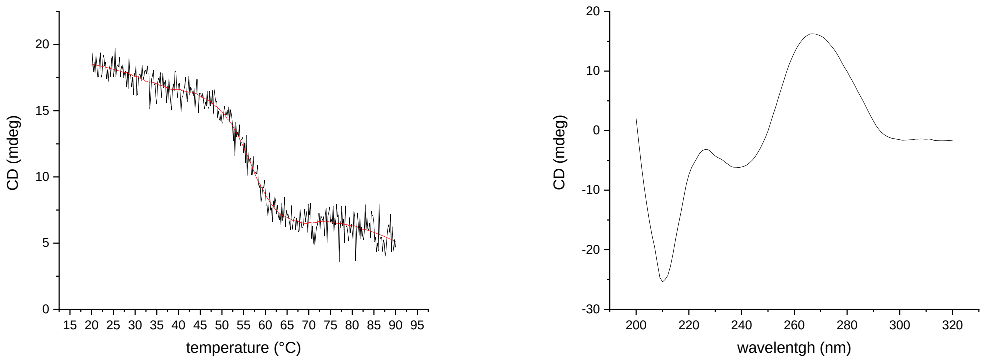

Figure SI-50. X = U; Duplex **12:3** CD data recorded at 270 nm (left) and full CD spectrum of duplex (right)

Experimental conditions: 2.5 µM RNA and 3.0 µM complement RNA prepared in PBS (10 mM NaCl, 5 mM $MgCl_2$, 1 mM Na_2HPO_4 pH 7.2)

5'-AAG-AG**G**^{Br}-GAU-GAC-3'
3'-UUC-UC**X**-CUA-CUG-5'

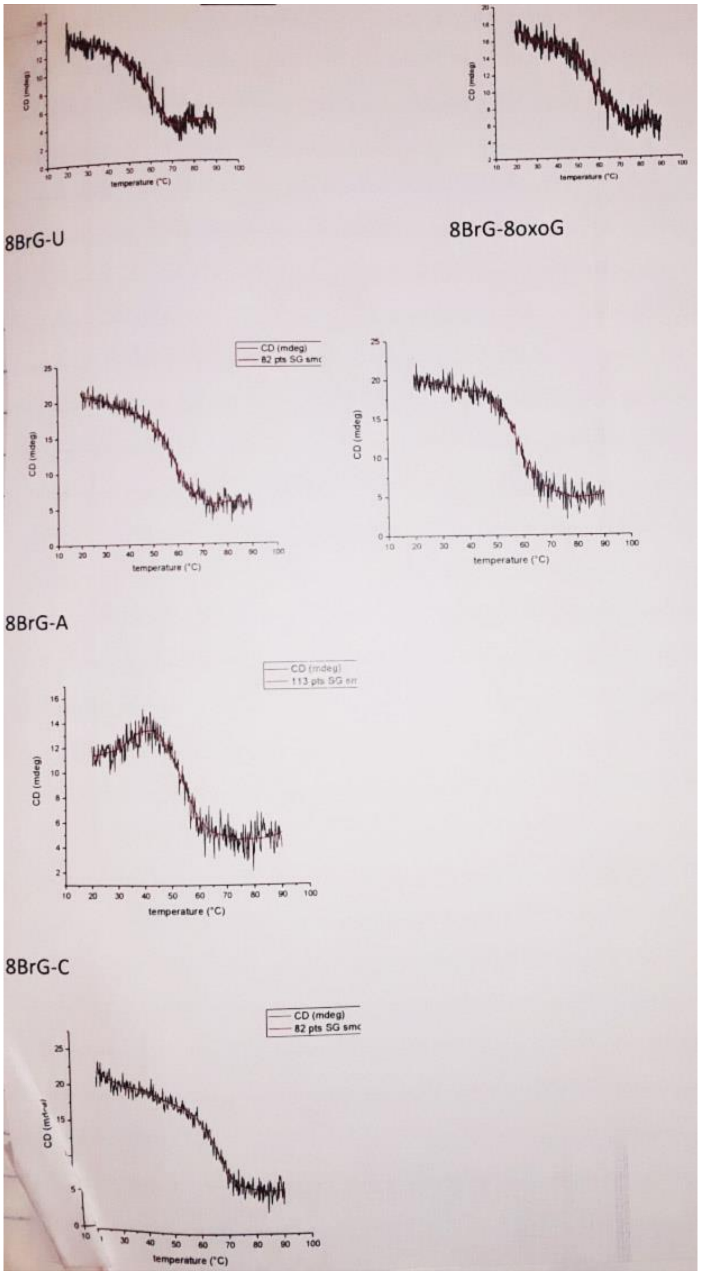

Figure SI-51. X = G (top left), I (top right), U (middle left), 8-oxoG (middle-middle), A (middle-lower left), and C (bottom); Duplexes **9:2-9:7** CD spectrum of duplexes shown

Table SI-1: Compiled T_m data for inosine containing dodecamer versus complementary strands

5'-AAG-AG**I**-GAU-GAC	T_m (°C)					
UUC-UC**X**-CUA-CUG-5'	1	2	3	avg	std dev	RSD
G	54.0	55.5	54.0	54.5	0.9	1.6%
U	62.4	62.0	62.6	62.3	0.3	0.5%
A	59.6	59.8	60.0	59.8	0.2	0.3%
C	66.8	67.4	67.6	67.3	0.4	0.6%
I	54.2	53.6	55.2	54.3	0.8	1.5%
8oxoG	62.6	62.4	62.8	62.6	0.2	0.3%

Table SI-2: Compiled T_m data for 8-oxo-inosine containing dodecamer versus complementary strands

5'-AAG-AG**I**^{oxo}-GAU-GAC	T_m (°C)					
UUC-UC**X**-CUA-CUG-5'	1	2	3	avg	std dev	RSD
G	59.2	60.6	59.2	59.7	0.8	1.4%
U	54.8	53.2	54.8	54.3	0.9	1.7%
A	59.4	59.0	59.6	59.3	0.3	0.5%
C	58.4	59.6	59.8	59.3	0.8	1.3%
I	57.0	57.4	57.4	57.3	0.2	0.4%
8oxoG	54.8	55.6	55.8	55.4	0.5	1.0%

Above duplexes at pH 8.55	T_m (°C)	ΔT_m (°C)
G	58.9	0.8
U	54.6	0.3
	59.8	0.5
C	59.8	0.5

Table SI-3: Compiled T_m data for guanosine containing dodecamer versus complementary strands

5'-AAG-AG**G**-GAU-GAC	T_m (°C)					
UUC-UC**X**-CUA-CUG-5'	1	2	3	avg	std dev	RSD
G	58.2	58.2	58.8	58.4	0.3	0.6%
U	64.8	64.8	65	64.9	0.1	0.2%
A*	58.2					
C*	74.6					
I	57.2	57	58	57.4	0.5	0.9%
8oxoG	62.8	63.2	62.4	62.8	0.4	0.6%

*Experiment only run once to verify previously reported T_m value

Table SI-4: Compiled T_m data for 8-oxo-guanosine containing dodecamer versus complementary strands

5'-AAG-AG**G**^{oxo}-GAU-GAC	T_m (°C)					
UUC-UC**X**-CUA-CUG-5'	1	2	3	avg	std dev	RSD
G	58.6	59.3	60.2	59.4	0.8	1.4%
U	57.0	57.2	57.2	57.1	0.1	0.2%
A	62	61.4	61.8	61.7	0.3	0.5%
C*	62.4			62.4		
I	59.8	60	59.8	59.9	0.1	0.2%
8oxoG	56.7	56.6	55.8	56.4	0.5	0.9%

*Experiment only run once to verify previously reported T_m value

Table SI-5: Compiled T_m data for adenosine containing dodecamer versus complementary strands

5'-AAG-AG**A**-GAU-GAC	T_m (°C)					
UUC-UC**X**-CUA-CUG-5'	1	2	3	avg	std dev	RSD
G	56.2	56.0	55.8	56.0	0.2	0.4%
U	68.6	69.2	68.6	68.8	0.3	0.5%
A	53.0	53.2	53.6	53.3	0.3	0.6%
C	59.4	59.4	59.6	59.5	0.1	0.2%
I	61.0	61.8	61.2	61.3	0.4	0.7%
8oxoG	65.8	66.0	66.0	65.9	0.1	0.2%

Table SI-6: Compiled T_m data for 8-oxo-adenosine containing dodecamer versus complementary strands

5'-AAG-AG**A**^{oxo}-GAU-GAC	T_m (°C)					
UUC-UC**X**-CUA-CUG-5'	1	2	3	avg	std dev	RSD
G	56.2	56.0	57.0	56.4	0.5	1%
U	57.4	57.2	56.8	57.1	0.3	1%
A	56.0	56.6	56.2	56.3	0.3	0.5%
C	51.1	51.2	51.6	51.3	0.3	1%
I	54.1	55.4	54.4	54.6	0.7	1%
8oxoG	56.3	56.4	56.6	56.4	0.2	0%

Table SI-7: Compiled T_m data for 8-oxo-adenosine containing dodecamer versus complementary strands

5'-AAG-AG**I**^{Br}-GAU-GAC	T_m (°C)					
UUC-UC**X**-CUA-CUG-5'	1	2	3	avg	std dev	RSD
G	58.1	58.2	59.0	58.4	0.5	0.8%
U	56.8	56.5	56.6	56.6	0.2	0.3%
A	57.2	56.7	57.4	57.1	0.4	0.6%
C	59.6	59.4	60.2	59.7	0.4	0.7%
I	57.6	58.2	56.7	57.5	0.8	1.3%
8oxoG	56.6	56.0	56.6	56.4	0.3	0.6%

Table SI-8: Compiled T_m data for 8-oxo-adenosine containing dodecamer versus complementary strands

5'-AAG-AGG^{Br}-GAU-GAC-3'	T_m (ºC)					
3'-UUC-UCX-CUA-CUG-5'	1	2	3	Avg	std dev	RSD
G	59.9	61.2	60.4	60.5	0.7	1.1%
U	57.4	58.2	58.0	57.9	0.4	0.7%
A	57.6	57.2	57.4	57.4	0.2	0.3%
C	66.4	65.8	66.7	66.3	0.5	0.7%
I	60.2	61.6	61.4	61.1	0.8	1.2%
8oxoG	59	58.4	58.6	58.7	0.3	0.5%

Electronic Structure Theory Calculations of Free energies

Electronic structure theory calculations, mainly employing density functional theory (DFT), were used to optimize the structures of the compounds and obtain the free energies for dimerization. To assess the accuracy/uncertainty of the results, we selected eight base pairs for calibration calculations. The calibration included three different DFT functionals and two basis sets. Since H-bond is a weak interaction and difficult to be described with some popular DFT functionals such as B3LYP, we included dispersion corrections either using functional M06-2X[1] or using empirical dispersion correction D3(BJ)[2]. As shown in Table SI-9, B3LYP-D3(BJ)/6-31G* provides smaller values than other levels of theory. In addition, there are some negative values in the M06-2X/6-31G* and B3LYP-D3(BJ)/6-31G* results. Nevertheless, for most base pairs the differences in free energies are within 5% and this is reasonable regarding typical errors in DFT calculations. Eventually B3LYP-D3(BJ) is chosen in the paper because not only the results are similar to those from M06-2X/6-31+G* but also the computation times of frequency calculations can be reduced about 40%.

Table SI-9: Comparison of dimerization free energies (kcal mol^{-1}) at various level of theory for anti-G:anti-C and anti-8G: anti-C. T_m is the experimental melting temperature.

	M06-2X/ 6-31G*	B3LYP/ 6-31G*	B3LYP-D3(BJ)/ 6-31G*	M06-2X/ 6-31+G*	B3LYP/ 6-31+G*	B3LYP-D3(BJ) /6-31+G*	T_m (ºC)
anti-G:anti-C	3.74	4.01	-1.43	2.82	3.10	4.12	72.6
anti-8oxoG: anti-C	3.50	4.15	-2.90	2.69	3.36	4.31	62.8
anti-G:anti-A	-5.60	3.31	1.20	7.88	5.28	4.72	55.9
syn-I:anti-A	-3.94	4.73	1.80	8.18	7.70	8.2	59.8
anti-G:syn-G	6.40	7.95	0.26	6.99	3.63	4.4	58.4
anti-8-BrG:anti-C	1.48	3.27	-0.77	4.60	4.28	4.41	66.3
anti-G:anti-U	2.15	2.94	0.79	5.37	4.81	5.76	64.9
anti-8BrG:anti-U	3.62	3.59	3.67	4.50	4.94	5.04	57.9

In addition to DFT, second-order Møller-Plesset perturbation theory (MP2) is also used. Since the computation cost is high, only three pairs were calibrated. The optimization was performed at the MP2/6-31+G* level of theory. In Table SI-10, the first column uses MP2/6-31+G* to calculate the electronic energies and the second column uses MP2/6-311++G** to calculate the electronic energies. The solvent free energy and Gibbs free

energy corrections are the same, in which MP2/6-31+G* was used. The basis set changes the value is ~1 kcal/mol but the trend does not change.

Table SI-10: Comparison of dimerization free energies (kcal mol^{-1}) with the MP2/6-31+G* optimized structures. The first and second columns are electronic energies using MP2/ 6-31+G* and MP2/6-311++G**, respectively.

	6-31+G*	6-311++G**	T_m (ºC)
anti-G:anti-C	1.88	0.86	72.6
anti-8oxoG: anti-C	1.12	0.10	62.8
anti8oxoI-antiC	3.59	2.65	59.3

antiG:antiC – entry1

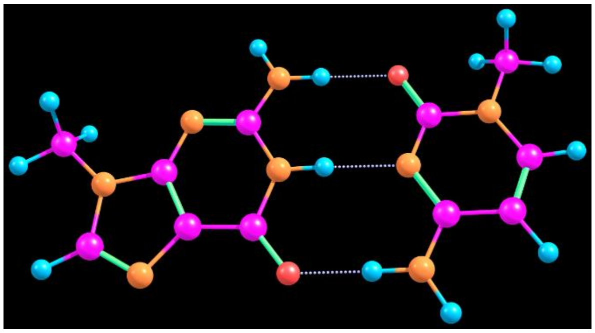

7	-0.697352000	-0.048183000	0.000033000
1	0.331527000	0.054892000	0.000214000
6	-1.236498000	-1.312969000	-0.000357000
7	-2.542868000	-1.545674000	-0.000437000
6	-3.272172000	-0.409455000	-0.000200000
7	-0.365271000	-2.344697000	-0.000908000
1	-0.755096000	-3.274800000	-0.000365000
1	0.651533000	-2.217125000	0.000145000
7	-4.643377000	-0.340770000	-0.000479000
6	-4.961152000	1.012194000	-0.000057000
1	-5.992201000	1.342096000	-0.000169000
7	-3.909568000	1.792137000	0.000484000
6	-2.832100000	0.921411000	0.000170000
6	-1.424003000	1.159523000	0.000234000
8	-0.814605000	2.242265000	0.000584000
6	-5.551078000	-1.472897000	0.000084000
1	-5.384900000	-2.093671000	-0.885351000
1	-6.577224000	-1.098359000	-0.007071000
1	-5.394500000	-2.085966000	0.892697000
1	0.925895000	2.474874000	-0.000138000
7	1.955668000	2.597831000	-0.000814000
1	2.336005000	3.532514000	-0.000650000
6	2.771226000	1.536771000	-0.000444000
7	2.214110000	0.319541000	-0.000097000
6	2.972410000	-0.804125000	0.000361000
8	2.499372000	-1.950893000	0.000768000
7	4.381569000	-0.668379000	0.000370000
6	4.951821000	0.567854000	-0.000052000
1	6.036575000	0.591757000	-0.000019000
6	4.199993000	1.700072000	-0.000460000
1	4.663269000	2.679182000	-0.000771000
6	5.180486000	-1.893053000	0.000841000
1	6.238076000	-1.621156000	0.000829000

1	4.949395000	-2.489611000	0.886603000
1	4.949530000	-2.490212000	-0.884552000

antiI:antiC entry 2

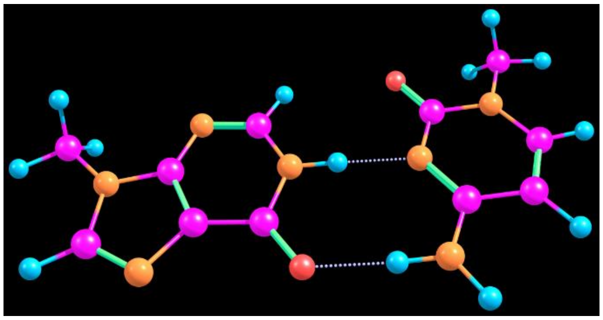

7	-0.652484000	0.018545000	-0.000017000
1	0.383686000	0.132680000	0.000068000
6	-1.158764000	-1.242097000	-0.000044000
7	-2.438403000	-1.532709000	-0.000146000
6	-3.212352000	-0.417296000	-0.000323000
7	-4.585757000	-0.389400000	-0.000462000
6	-4.937651000	0.947832000	-0.000569000
1	-5.976580000	1.252146000	-0.000741000
7	-3.906122000	1.760812000	-0.000669000
6	-2.808082000	0.924248000	-0.000320000
6	-1.400621000	1.211300000	-0.000163000
8	-0.829195000	2.311491000	-0.000237000
6	-5.463501000	-1.547521000	0.000566000
1	-5.279413000	-2.161809000	-0.885153000
1	-6.498885000	-1.200246000	-0.005839000
1	-5.287957000	-2.154451000	0.893140000
1	0.969503000	2.594506000	-0.000309000
7	1.997624000	2.686315000	-0.000366000
1	2.409847000	3.606915000	-0.000363000
6	2.772379000	1.590182000	-0.000126000
7	2.163600000	0.403107000	0.000002000
6	2.859571000	-0.766735000	0.000234000
8	2.325766000	-1.877484000	0.000345000
7	4.279788000	-0.687579000	0.000313000
6	4.903557000	0.520805000	0.000177000
1	5.988594000	0.496400000	0.000253000
6	4.206087000	1.689374000	-0.000042000
1	4.715554000	2.645234000	-0.000154000
6	5.021220000	-1.947016000	0.000553000
1	6.090689000	-1.725786000	0.000592000
1	4.761467000	-2.532883000	0.885683000
1	4.761588000	-2.533150000	-0.884436000
1	-0.415509000	-2.034299000	0.000095000

Anti-8oxoG:anti-C   entry 3

7	-0.367431000	-0.146337000	-0.001538000
1	0.658385000	-0.000994000	-0.001263000
6	-0.865939000	-1.422727000	-0.001434000
7	-2.169624000	-1.693862000	-0.000561000
6	-2.947807000	-0.597890000	-0.000350000
7	0.029074000	-2.432250000	-0.002251000
1	-0.336604000	-3.372165000	-0.000356000
1	1.042030000	-2.275227000	-0.001530000
7	-4.326045000	-0.588225000	-0.002653000
6	-4.802826000	0.739249000	-0.000008000
7	-3.667100000	1.527359000	-0.001098000
6	-2.522681000	0.725587000	-0.000467000
6	-1.149052000	1.024763000	-0.001218000
8	-0.606368000	2.153484000	-0.001537000
6	-5.205856000	-1.741394000	0.005188000
1	-4.588770000	-2.633844000	-0.106893000
1	-5.918483000	-1.669395000	-0.820805000
1	-5.762940000	-1.790883000	0.946027000
1	1.133190000	2.452201000	0.000125000
7	2.157476000	2.612342000	0.001111000
1	2.503554000	3.560245000	0.001075000
6	3.012959000	1.582356000	0.000840000
7	2.502200000	0.345140000	0.000177000
6	3.301811000	-0.750205000	0.000013000
8	2.870430000	-1.913124000	-0.000628000
7	4.704216000	-0.561730000	0.000514000
6	5.227622000	0.694920000	0.001205000
1	6.310679000	0.759395000	0.001578000
6	4.434097000	1.798674000	0.001391000
1	4.860791000	2.794239000	0.001945000
6	5.548735000	-1.756002000	0.000381000
1	6.595281000	-1.444620000	0.000383000
1	5.340141000	-2.360953000	0.885969000
1	5.340079000	-2.360751000	-0.885321000
8	-5.977899000	1.082887000	0.000893000
1	-3.692379000	2.535732000	0.000094000

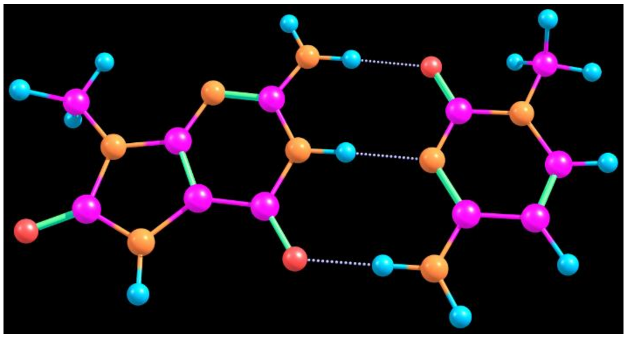

Anti-8BrG:antiC   entry 4

7	-0.575513000	0.275891000	0.000337000
1	-1.587398000	0.058986000	0.000029000
6	-0.179411000	1.592053000	0.000724000
7	1.094527000	1.967349000	0.000768000
6	1.944294000	0.922122000	0.000379000
7	-1.156923000	2.521535000	0.000995000
1	-0.871842000	3.489029000	0.001221000
1	-2.154140000	2.282930000	0.000250000
7	3.320544000	1.005301000	0.000176000
6	3.765021000	-0.309471000	-0.000102000
7	2.818959000	-1.198994000	-0.000247000
6	1.652366000	-0.447256000	0.000013000
6	0.280654000	-0.843041000	-0.000182000
8	-0.199939000	-1.987679000	-0.000709000
6	4.110921000	2.223606000	0.000212000
1	3.413881000	3.063009000	-0.001146000
1	4.744391000	2.267402000	-0.890247000
1	4.742499000	2.268740000	0.891963000
1	-1.907837000	-2.414283000	-0.000307000
7	-2.917479000	-2.647467000	0.000361000
1	-3.194147000	-3.617911000	0.000499000
6	-3.843187000	-1.680927000	0.000229000
7	-3.421844000	-0.410094000	-0.000606000
6	-4.298349000	0.624210000	-0.000866000
8	-3.952440000	1.815757000	-0.001491000
7	-5.683722000	0.336193000	-0.000260000
6	-6.116063000	-0.954763000	0.000803000
1	-7.191736000	-1.096545000	0.001467000
6	-5.245453000	-1.998453000	0.001057000
1	-5.599557000	-3.022097000	0.001983000
6	-6.611366000	1.466838000	-0.000595000
1	-7.632983000	1.081378000	-0.000055000
1	-6.446419000	2.085683000	0.884609000
1	-6.446988000	2.084668000	-0.886614000
35	5.606997000	-0.676375000	-0.000193000

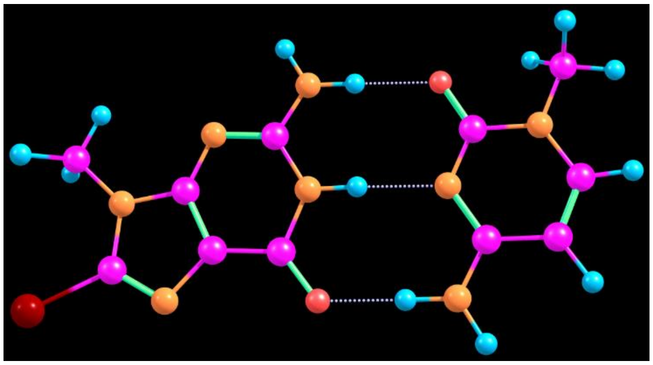

Anti-8oxoI:antiC   entry 5

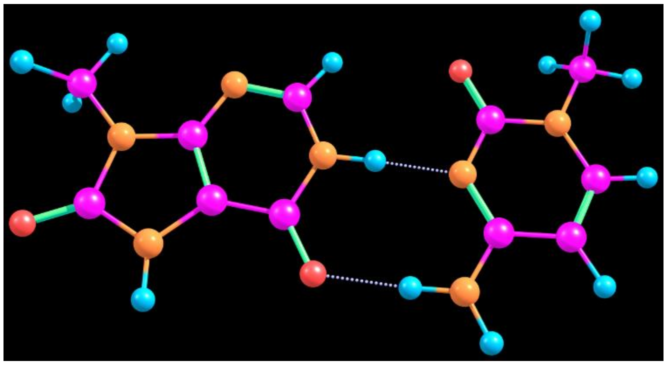

7	-0.316267000	-0.220941000	-0.000512000
1	0.719026000	-0.073648000	-0.000574000
6	-0.795941000	-1.487922000	-0.000721000
7	-2.074031000	-1.801687000	-0.000579000
6	-2.885932000	-0.720288000	-0.000198000
7	-4.264739000	-0.734735000	-0.000126000
6	-4.759268000	0.582260000	0.000405000
7	-3.627289000	1.388522000	0.000419000
6	-2.479920000	0.607655000	0.000040000
6	-1.103829000	0.940553000	-0.000134000
8	-0.593074000	2.080609000	0.000013000
6	-5.127002000	-1.902854000	0.000235000
1	-4.490263000	-2.788394000	-0.007030000
1	-5.768802000	-1.895225000	-0.885268000
1	-5.758714000	-1.903000000	0.893075000
1	1.207292000	2.421560000	-0.000516000
7	2.231943000	2.541792000	-0.000717000
1	2.618659000	3.473398000	-0.000056000
6	3.038115000	1.467824000	-0.000299000
7	2.463691000	0.263969000	-0.000503000
6	3.192318000	-0.886707000	-0.000194000
8	2.687381000	-2.010497000	-0.000244000
7	4.608782000	-0.767494000	0.000386000
6	5.197825000	0.458023000	0.000565000
1	6.283063000	0.464329000	0.001000000
6	4.467879000	1.606939000	0.000226000
1	4.950676000	2.576487000	0.000364000
6	5.385576000	-2.005983000	0.000796000
1	6.448268000	-1.754692000	0.001310000
1	5.141871000	-2.598667000	0.885867000
1	5.142742000	-2.598710000	-0.884484000
8	-5.934485000	0.917908000	0.000712000
1	-3.665439000	2.397266000	0.000562000
1	-0.040492000	-2.267837000	-0.000992000

Anti-8BrI:antiC   entry 6

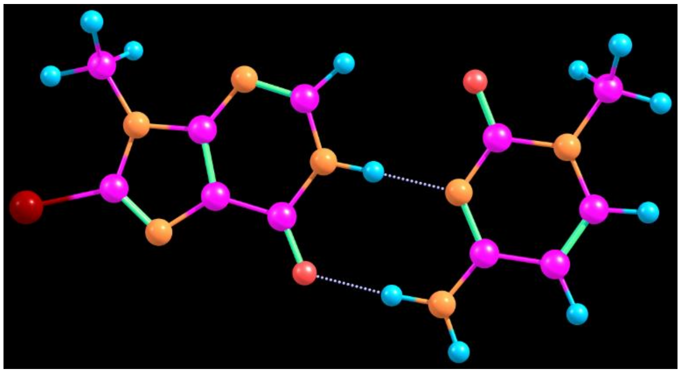

7	-0.664117000	0.322638000	-0.000507000
1	-1.686414000	0.110780000	-0.001517000
6	-0.278604000	1.624104000	0.000121000
7	0.969170000	2.034123000	0.001065000
6	1.842061000	0.997236000	0.000868000
7	3.216039000	1.104119000	0.000555000
6	3.676558000	-0.201237000	0.000244000
7	2.737373000	-1.105359000	0.000162000
6	1.564883000	-0.373284000	0.000337000
6	0.192187000	-0.795035000	-0.000126000
8	-0.270143000	-1.944169000	-0.000456000
6	3.956480000	2.356475000	0.002078000
1	3.689195000	2.943366000	-0.880504000
1	5.022869000	2.134473000	-0.011665000
1	3.709709000	2.930570000	0.899088000
1	-2.042627000	-2.398336000	-0.000467000
7	-3.058057000	-2.579018000	0.000394000
1	-3.388640000	-3.531985000	0.001309000
6	-3.924935000	-1.553950000	0.000300000
7	-3.421381000	-0.318668000	-0.000920000
6	-4.215464000	0.787183000	-0.001416000
8	-3.777930000	1.939250000	-0.002420000
7	-5.622978000	0.585889000	-0.000501000
6	-6.139954000	-0.671926000	0.000950000
1	-7.223018000	-0.741213000	0.001707000
6	-5.344433000	-1.776219000	0.001382000
1	-5.769794000	-2.772316000	0.002496000
6	-6.470484000	1.776770000	-0.001136000
1	-7.516787000	1.463980000	-0.000127000
1	-6.261785000	2.383729000	0.883253000
1	-6.262964000	2.381948000	-0.887025000
1	-1.092594000	2.343375000	-0.000261000
35	5.513295000	-0.586737000	-0.000190000

Syn-G:anti-C   entry 7

7	0.814300000	-0.547110000	-0.013890000
6	0.453740000	0.719120000	-0.009400000
1	-0.576050000	1.057560000	-0.011250000
7	1.537290000	1.575610000	-0.001500000
6	2.660430000	0.786410000	-0.000600000
7	3.945510000	1.230370000	-0.007100000
6	4.822680000	0.255550000	0.005840000
7	4.478160000	-1.073190000	0.009400000
1	5.198750000	-1.782640000	0.091730000
6	2.195450000	-0.530610000	-0.009450000
7	6.169670000	0.547950000	0.075770000
1	6.367200000	1.527600000	-0.091160000
1	6.806030000	-0.077540000	-0.403630000
6	3.145370000	-1.604980000	-0.000020000
8	2.983870000	-2.818710000	0.008540000
6	1.501700000	3.029620000	0.005730000
1	0.457370000	3.346950000	0.004970000
1	2.005250000	3.423960000	-0.881390000
1	2.001290000	3.415010000	0.898930000
1	-0.721590000	-1.734980000	-0.016650000
7	-1.660500000	-2.156770000	-0.015330000
1	-1.750120000	-3.161180000	-0.003090000
6	-2.751500000	-1.366780000	-0.007340000
7	-2.562180000	-0.049830000	-0.013010000
6	-3.609880000	0.816730000	-0.006160000
8	-3.494210000	2.043960000	-0.010110000
7	-4.929670000	0.267000000	0.006380000
6	-5.118090000	-1.078980000	0.011940000
1	-6.149600000	-1.417460000	0.021760000
6	-4.069550000	-1.945580000	0.005570000
1	-4.228900000	-3.017270000	0.009920000
6	-6.046580000	1.206620000	0.013900000
1	-6.982490000	0.642830000	0.022360000
1	-5.990490000	1.848560000	0.896870000
1	-6.004690000	1.845660000	-0.871930000

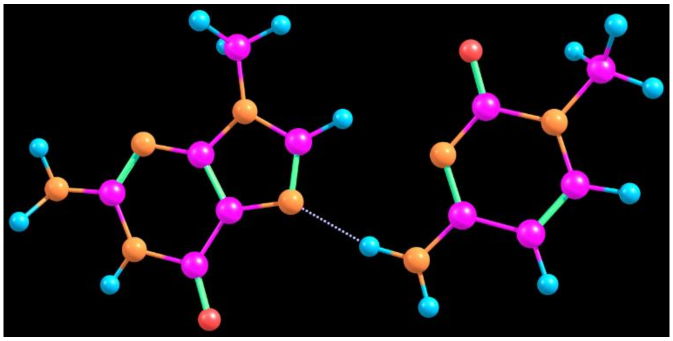

Syn-I:anti-C   entry 8

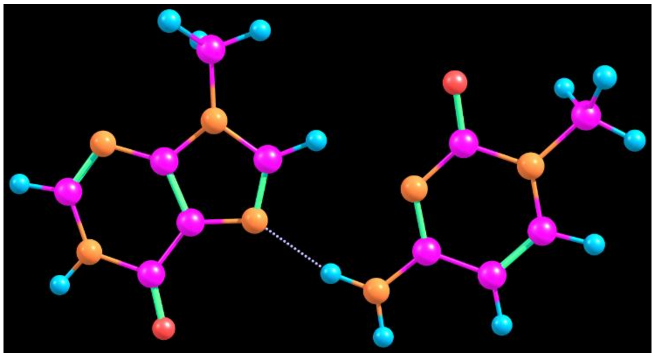

7	0.817795000	-0.553666000	0.004465000
6	0.447957000	0.713961000	0.003558000
1	-0.585822000	1.041062000	0.002772000
7	1.519997000	1.576541000	0.003857000
6	2.649746000	0.794385000	0.004992000
7	3.936979000	1.240600000	0.005541000
6	4.803176000	0.266016000	0.006567000
7	4.472695000	-1.060854000	0.006934000
1	5.210928000	-1.757357000	0.007770000
6	2.193949000	-0.527196000	0.005347000
6	3.149363000	-1.604386000	0.006438000
8	2.981731000	-2.816831000	0.006906000
6	1.472323000	3.031659000	0.002956000
1	0.425272000	3.339507000	0.002965000
1	1.971107000	3.423403000	-0.887508000
1	1.971441000	3.424454000	0.892761000
1	-0.723271000	-1.764679000	0.003415000
7	-1.663377000	-2.180593000	0.002759000
1	-1.759355000	-3.184452000	0.005095000
6	-2.748448000	-1.381833000	0.003460000
7	-2.547351000	-0.066830000	0.002634000
6	-3.586932000	0.809709000	0.002997000
8	-3.457903000	2.035616000	0.002481000
7	-4.911169000	0.272419000	0.004114000
6	-5.111756000	-1.071797000	0.004982000
1	-6.146340000	-1.400764000	0.005880000
6	-4.071314000	-1.948445000	0.004721000
1	-4.240851000	-3.018541000	0.005337000
6	-6.019648000	1.222396000	0.004531000
1	-6.960629000	0.667198000	0.004889000
1	-5.964290000	1.862247000	0.888996000
1	-5.964918000	1.862192000	-0.879999000
1	5.865794000	0.492105000	0.007141000

Syn-8oxoG:anti-C   entry 9

7	-0.120789000	1.343951000	0.341181000
6	0.001936000	2.688461000	0.634959000
7	1.396269000	2.968705000	0.527982000
6	2.066225000	1.835813000	0.160809000
7	3.404188000	1.738832000	-0.028801000
6	3.812653000	0.527595000	-0.332968000
7	2.965525000	-0.541041000	-0.441267000
1	3.335538000	-1.476315000	-0.573912000
6	1.127617000	0.816356000	0.028177000
7	5.160371000	0.280710000	-0.498279000
1	5.707658000	1.131637000	-0.555706000
1	5.422594000	-0.410554000	-1.190924000
6	1.556616000	-0.500965000	-0.234139000
8	0.914591000	-1.564265000	-0.293666000
6	1.967279000	4.277193000	0.775014000
1	1.142738000	4.937625000	1.048955000
1	2.462267000	4.655299000	-0.124770000
1	2.695058000	4.230775000	1.590929000
1	-0.802439000	-1.815897000	0.333687000
7	-1.743968000	-2.038275000	0.679002000
1	-1.903538000	-2.965965000	1.042273000
6	-2.795936000	-1.283123000	0.299060000
7	-2.541050000	-0.121891000	-0.298853000
6	-3.551269000	0.688036000	-0.742284000
8	-3.375783000	1.738252000	-1.349285000
7	-4.892632000	0.274014000	-0.472080000
6	-5.144150000	-0.900750000	0.157668000
1	-6.190052000	-1.144846000	0.316169000
6	-4.137840000	-1.725248000	0.558769000
1	-4.347197000	-2.664731000	1.055694000
6	-5.961601000	1.163854000	-0.918216000
1	-6.921923000	0.740964000	-0.614367000
1	-5.829939000	2.152712000	-0.472613000
1	-5.928669000	1.274293000	-2.005282000
8	-0.856235000	3.499329000	0.943101000
1	-1.021190000	0.888968000	0.110269000

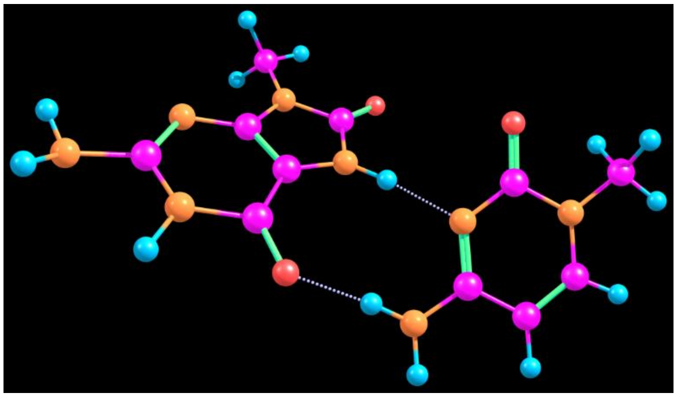

Syn-G:syn-C   entry 10

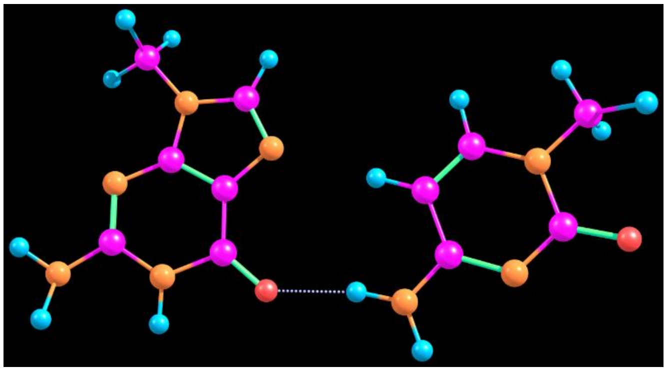

7	3.297690000	-1.839990000	0.001970000
1	3.317210000	-2.848750000	0.070850000
6	4.472530000	-1.128570000	-0.086600000
7	4.527510000	0.179280000	-0.101990000
6	3.305810000	0.762440000	-0.005150000
7	5.637180000	-1.863090000	-0.105330000
1	6.457540000	-1.312540000	-0.329550000
1	5.620390000	-2.777410000	-0.534380000
7	3.057970000	2.112200000	0.022370000
6	1.681900000	2.247530000	0.115180000
1	1.215790000	3.223070000	0.154580000
7	1.050810000	1.097860000	0.153170000
6	2.051100000	0.146750000	0.077670000
6	1.997090000	-1.271130000	0.087140000
8	1.015600000	-2.049340000	0.163410000
6	4.050930000	3.170360000	-0.079470000
1	5.022720000	2.759110000	0.196960000
1	3.792440000	3.982180000	0.604490000
1	4.105000000	3.557450000	-1.102080000
1	-2.276380000	-3.100900000	0.045290000
7	-1.924240000	-2.154200000	0.084590000
1	-0.922380000	-1.995490000	0.131470000
6	-2.828430000	-1.148580000	0.043260000
7	-4.108180000	-1.476000000	-0.035570000
6	-5.074860000	-0.508970000	-0.079250000
8	-6.281720000	-0.723890000	-0.156230000
7	-4.642270000	0.855720000	-0.030220000
6	-3.328230000	1.178250000	0.048770000
1	-3.085200000	2.231080000	0.078260000
6	-2.353610000	0.207360000	0.088180000
1	-1.308020000	0.463030000	0.149660000
6	-5.681830000	1.880060000	-0.074510000
1	-5.209390000	2.864120000	-0.020500000
1	-6.257780000	1.793510000	-1.000090000
1	-6.372620000	1.751930000	0.763290000

Syn-I:syn-C   entry 11

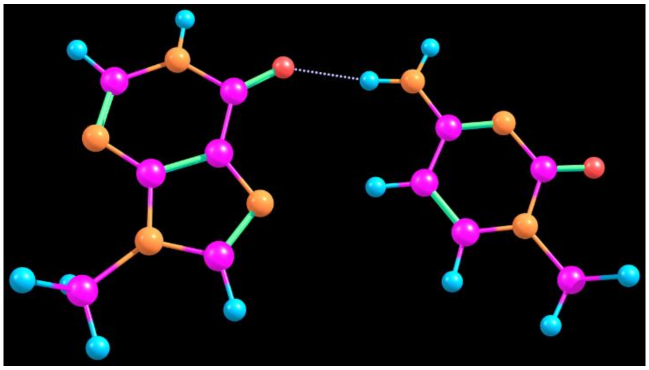

7	3.300295000	-1.825266000	-0.013154000
1	3.334542000	-2.840127000	-0.014229000
6	4.468769000	-1.115349000	-0.017891000
7	4.537685000	0.186864000	-0.017108000
6	3.309976000	0.773273000	-0.011043000
7	3.059900000	2.122314000	-0.008862000
6	1.685655000	2.252639000	-0.002521000
1	1.214547000	3.226879000	0.000388000
7	1.052336000	1.099871000	-0.000583000
6	2.054720000	0.155722000	-0.005860000
6	1.985587000	-1.280434000	-0.006790000
8	1.015619000	-2.032508000	-0.003104000
6	4.059466000	3.181068000	-0.012293000
1	4.692638000	3.102710000	0.875158000
1	3.545337000	4.143925000	-0.009449000
1	4.685167000	3.103975000	-0.905133000
1	-2.306442000	-3.124990000	0.013556000
7	-1.946148000	-2.181485000	0.012631000
1	-0.942340000	-2.028310000	0.007217000
6	-2.839715000	-1.166736000	0.016465000
7	-4.128850000	-1.481521000	0.022158000
6	-5.082835000	-0.507008000	0.026358000
8	-6.296290000	-0.709566000	0.031001000
7	-4.638609000	0.856017000	0.025272000
6	-3.312557000	1.158722000	0.019097000
1	-3.064106000	2.216072000	0.018239000
6	-2.359612000	0.189500000	0.014497000
1	-1.303119000	0.430942000	0.009813000
6	-5.667433000	1.889067000	0.029418000
1	-5.183602000	2.869272000	0.031535000
1	-6.304475000	1.790161000	-0.853753000
1	-6.301922000	1.784973000	0.913731000
1	5.382392000	-1.702488000	-0.022538000

Anti-G:anti-U   entry 12

7	0.873151000	0.679830000	-0.030066000
1	-0.128350000	0.920405000	-0.035366000
6	1.797671000	1.686559000	-0.028307000
7	3.099856000	1.486914000	-0.008944000
6	3.418597000	0.168878000	-0.004445000
7	1.302735000	2.964240000	-0.097176000
1	1.963054000	3.691874000	0.140801000
1	0.345151000	3.121889000	0.190120000
7	4.690218000	-0.344731000	0.003973000
6	4.545363000	-1.725797000	0.013660000
7	3.295032000	-2.116213000	0.008704000
6	2.564722000	-0.942009000	-0.003207000
6	1.152583000	-0.706220000	-0.010056000
8	0.213350000	-1.510680000	-0.000035000
6	5.922028000	0.423930000	0.026268000
1	5.908325000	1.171269000	-0.769883000
1	6.761804000	-0.255687000	-0.128720000
1	6.041259000	0.934279000	0.986517000
6	-3.530214000	-1.818503000	-0.007365000
7	-2.541303000	-0.808731000	0.012761000
6	-2.756281000	0.540311000	0.024270000
8	-1.845311000	1.384077000	0.042671000
7	-4.081806000	0.952580000	0.014118000
6	-5.109792000	0.031671000	-0.007525000
1	-6.106519000	0.459180000	-0.015500000
6	-4.892786000	-1.300275000	-0.017746000
1	-5.709820000	-2.009287000	-0.034245000
6	-4.338583000	2.393021000	0.018544000
1	-5.418351000	2.549770000	0.008783000
1	-3.909612000	2.851844000	0.912914000
1	-3.891722000	2.860536000	-0.862523000
8	-3.220657000	-2.998932000	-0.014518000
1	5.409778000	-2.377263000	0.020165000
1	-1.549087000	-1.121148000	0.013850000

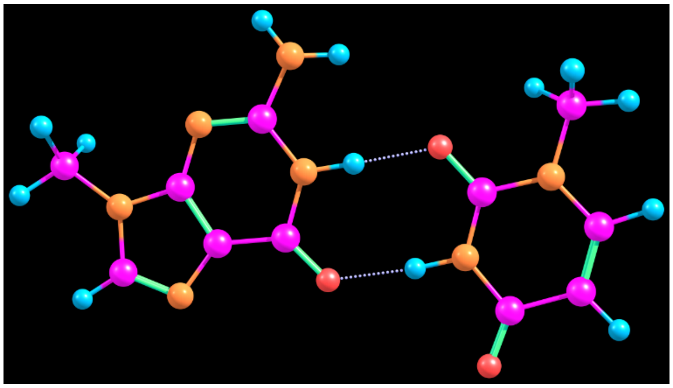

Anti-I:anti-U entry 13

7	0.897242000	0.740353000	0.007876000
1	-0.100788000	1.004271000	0.008593000
6	1.842714000	1.718596000	0.016145000
7	3.134896000	1.512072000	0.016168000
6	3.440066000	0.187401000	0.006881000
7	4.703473000	-0.349151000	0.004465000
6	4.530753000	-1.721080000	-0.005738000
7	3.270087000	-2.089875000	-0.009918000
6	2.564724000	-0.905488000	-0.002100000
6	1.149729000	-0.651553000	-0.001946000
8	0.212409000	-1.454701000	-0.008941000
6	5.951403000	0.396627000	0.011539000
1	6.014433000	1.036125000	-0.873140000
1	6.780935000	-0.313387000	0.006982000
1	6.012538000	1.022483000	0.906057000
6	-3.555165000	-1.839715000	-0.014338000
7	-2.564158000	-0.833897000	-0.006459000
6	-2.771115000	0.517546000	0.002363000
8	-1.856800000	1.354481000	0.009193000
7	-4.096246000	0.934535000	0.003458000
6	-5.126760000	0.017323000	-0.003689000
1	-6.122193000	0.448040000	-0.001947000
6	-4.915470000	-1.316146000	-0.012278000
1	-5.735832000	-2.021467000	-0.017866000
6	-4.345958000	2.376394000	0.013147000
1	-5.424958000	2.538430000	0.011831000
1	-3.906620000	2.830930000	0.904550000
1	-3.902581000	2.843444000	-0.869720000
8	-3.248998000	-3.021795000	-0.021952000
1	5.380503000	-2.391652000	-0.009637000
1	-1.573694000	-1.141007000	-0.007686000
1	1.464753000	2.736860000	0.023163000

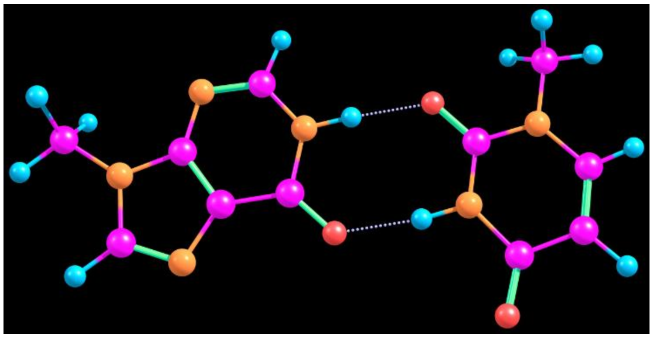

Anti-8oxoG:anti-U   entry 14

7	-0.856926000	-0.009043000	-0.082365000
1	0.164223000	0.136775000	-0.113010000
6	-1.360301000	-1.275802000	-0.087622000
7	-2.652508000	-1.552382000	-0.043753000
6	-3.433760000	-0.451744000	-0.005214000
7	-0.453890000	-2.297794000	-0.190664000
1	-0.813150000	-3.215835000	0.033928000
1	0.507193000	-2.117426000	0.069726000
7	-4.808334000	-0.446073000	0.027695000
6	-5.285577000	0.884448000	0.067974000
7	-4.146684000	1.670670000	0.051638000
6	-3.006794000	0.869824000	0.005439000
6	-1.630296000	1.170847000	-0.034013000
8	-1.075168000	2.286610000	-0.031830000
6	-5.681031000	-1.604780000	0.041290000
1	-5.272112000	-2.371403000	-0.622660000
1	-6.664465000	-1.284166000	-0.306877000
1	-5.772130000	-2.016065000	1.053486000
6	2.257322000	3.967082000	-0.333280000
7	1.727519000	2.661330000	-0.222115000
6	2.436620000	1.494362000	-0.199271000
8	1.912917000	0.371007000	-0.108836000
7	3.816622000	1.611346000	-0.283817000
6	4.416826000	2.850058000	-0.394211000
1	5.499473000	2.829735000	-0.456956000
6	3.711756000	4.000746000	-0.421876000
1	4.198307000	4.963574000	-0.508869000
6	4.599400000	0.374951000	-0.265596000
1	5.657271000	0.637172000	-0.316884000
1	4.403672000	-0.182282000	0.653825000
1	4.334990000	-0.254689000	-1.119282000
8	-6.456650000	1.232696000	0.104396000
8	1.521393000	4.941214000	-0.348094000
1	-4.169323000	2.679826000	0.065457000
1	0.692499000	2.578143000	-0.153885000

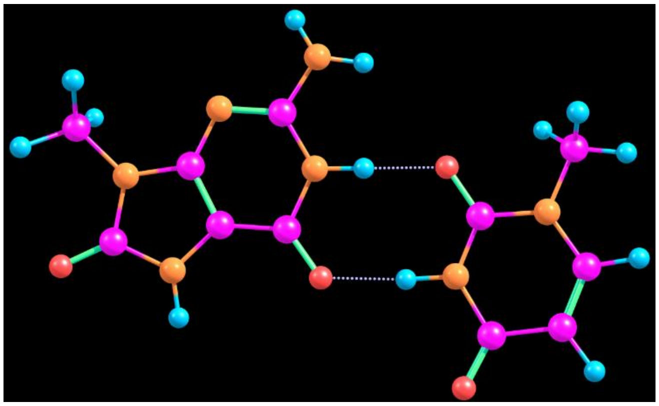

Anti-8BrG:anti-U   entry 15

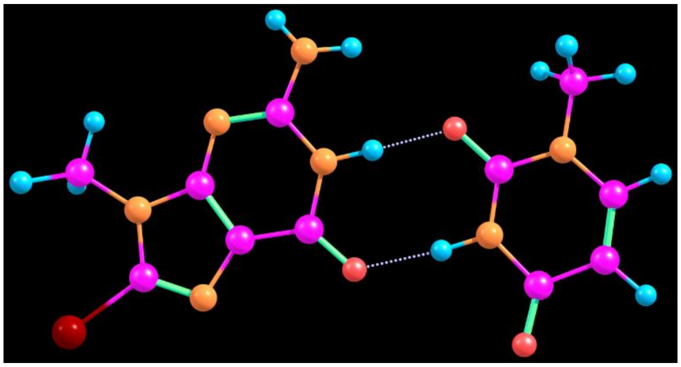

7	-0.470975000	1.124860000	-0.055911000
1	-1.501126000	1.159181000	-0.061130000
6	0.231371000	2.296649000	-0.042476000
7	1.548126000	2.362899000	-0.016693000
6	2.125539000	1.139049000	-0.014983000
7	-0.508419000	3.447703000	-0.103696000
1	-0.011975000	4.295572000	0.133494000
1	-1.484167000	3.408581000	0.161542000
7	3.479555000	0.891121000	0.004220000
6	3.595739000	-0.491437000	0.004593000
7	2.462195000	-1.126787000	-0.011843000
6	1.512049000	-0.118888000	-0.025384000
6	0.082092000	-0.176051000	-0.040912000
8	-0.672324000	-1.154860000	-0.041582000
6	4.541309000	1.883036000	0.018099000
1	4.069552000	2.866632000	0.009177000
1	5.177929000	1.770253000	-0.863698000
1	5.152080000	1.773350000	0.918400000
6	-4.288900000	-2.192938000	-0.021978000
7	-3.517768000	-1.007711000	-0.009816000
6	-3.993557000	0.272280000	0.020989000
8	-3.265577000	1.278932000	0.031535000
7	-5.373524000	0.415556000	0.040219000
6	-6.200622000	-0.689842000	0.030947000
1	-7.261806000	-0.466782000	0.048689000
6	-5.726443000	-1.952922000	0.001444000
1	-6.388397000	-2.808663000	-0.005796000
6	-5.908956000	1.777015000	0.071387000
1	-6.998333000	1.718101000	0.065244000
1	-5.575082000	2.295947000	0.973536000
1	-5.566841000	2.338111000	-0.801603000
8	-3.752357000	-3.288468000	-0.049496000
35	5.292650000	-1.289830000	0.028196000
1	-2.484592000	-1.120260000	-0.024060000

Anti-8oxoI:anti-U   entry 16

7	-0.593970000	-1.111267000	-0.000205000
1	0.421607000	-1.307310000	-0.000217000
6	-1.480806000	-2.138926000	0.000135000
7	-2.786062000	-2.001489000	0.000297000
6	-3.185244000	-0.707165000	0.000086000
7	-4.485765000	-0.258288000	0.000221000
6	-4.505181000	1.153237000	0.000001000
7	-3.165768000	1.525490000	-0.000369000
6	-2.353490000	0.403031000	-0.000310000
6	-0.944699000	0.253421000	-0.000358000
8	-0.082343000	1.149588000	-0.000540000
6	-5.685195000	-1.075891000	0.000433000
1	-5.711271000	-1.711927000	-0.889053000
1	-6.538565000	-0.395946000	-0.002855000
1	-5.714622000	-1.707167000	0.893215000
6	3.631520000	1.808770000	0.000255000
7	2.722704000	0.728490000	-0.000091000
6	3.033796000	-0.602413000	-0.000192000
8	2.185626000	-1.507357000	-0.000304000
7	4.386327000	-0.915248000	-0.000160000
6	5.342055000	0.079909000	0.000153000
1	6.367973000	-0.271895000	0.000175000
6	5.028007000	1.393287000	0.000314000
1	5.791466000	2.159852000	0.000502000
6	4.748140000	-2.333670000	0.000038000
1	5.836422000	-2.410762000	0.000741000
1	4.343604000	-2.827189000	0.887073000
1	4.344752000	-2.827186000	-0.887522000
8	-5.491931000	1.871583000	0.000027000
8	3.231080000	2.962832000	0.000469000
1	-2.857697000	2.487455000	-0.000537000
1	1.711612000	0.959150000	-0.000183000
1	-1.050419000	-3.135592000	0.000273000

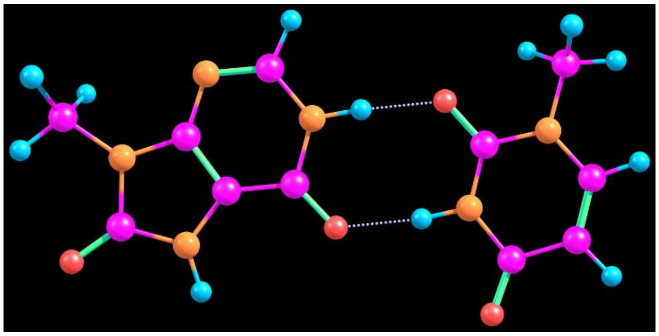

Anti-8BrI:anti-U   entry 17

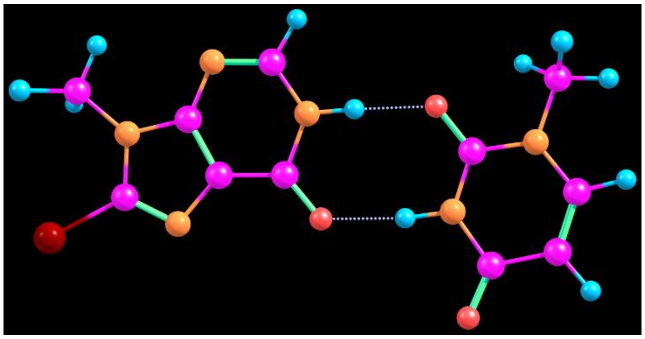

7	-0.459080000	1.188763000	0.007798000
1	-1.490734000	1.246382000	0.012388000
6	0.269704000	2.336257000	0.014613000
7	1.577502000	2.394015000	0.009830000
6	2.143241000	1.160615000	-0.002988000
7	3.493327000	0.889833000	-0.010697000
6	3.582350000	-0.489606000	-0.022902000
7	2.433504000	-1.106255000	-0.023582000
6	1.505449000	-0.083865000	-0.011096000
6	0.068903000	-0.123814000	-0.005628000
8	-0.684122000	-1.100674000	-0.010899000
6	4.572680000	1.864985000	-0.006691000
1	4.117249000	2.856126000	0.003981000
1	5.189495000	1.749512000	-0.901775000
1	5.197028000	1.733773000	0.880967000
6	-4.308912000	-2.219489000	-0.005827000
7	-3.534574000	-1.038154000	0.000818000
6	-4.002601000	0.246185000	0.013430000
8	-3.269884000	1.246499000	0.019291000
7	-5.383181000	0.395050000	0.019595000
6	-6.213775000	-0.706719000	0.013889000
1	-7.274296000	-0.479578000	0.019798000
6	-5.745266000	-1.972868000	0.001726000
1	-6.411490000	-2.825282000	-0.002716000
6	-5.911327000	1.759893000	0.033670000
1	-7.001088000	1.706696000	0.034255000
1	-5.568268000	2.289618000	0.925805000
1	-5.570069000	2.306914000	-0.848598000
8	-3.775699000	-3.317744000	-0.016856000
35	5.260880000	-1.318841000	-0.036740000
1	-2.504014000	-1.146260000	-0.003959000
1	-0.304179000	3.258259000	0.024792000

Syn-G:anti-U entry 18

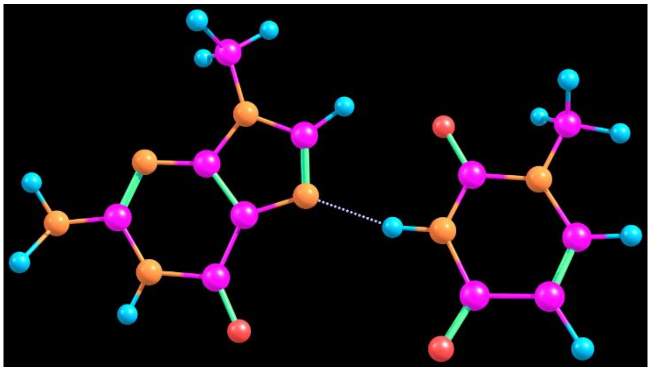

7	3.808604000	-1.518848000	0.120422000
1	4.262346000	-2.414641000	0.265939000
6	4.562099000	-0.380110000	0.004786000
7	4.044539000	0.822196000	-0.089058000
6	2.685887000	0.816364000	-0.044160000
7	5.932035000	-0.531521000	0.046720000
1	6.430695000	0.311847000	-0.210258000
1	6.320847000	-1.372356000	-0.362501000
7	1.883734000	1.930076000	-0.095365000
6	0.580898000	1.476790000	-0.032538000
7	0.505469000	0.166350000	0.056483000
6	1.816852000	-0.272394000	0.051656000
6	2.371678000	-1.594534000	0.163543000
8	1.838230000	-2.683794000	0.291776000
6	2.334393000	3.305826000	-0.225787000
1	3.162456000	3.486267000	0.462714000
1	1.503153000	3.970474000	0.021063000
1	2.670895000	3.507374000	-1.247346000
6	-2.965607000	-1.791869000	-0.185814000
7	-2.380641000	-0.518363000	-0.036302000
6	-3.036142000	0.683630000	0.081480000
8	-2.466405000	1.770055000	0.185948000
7	-4.431318000	0.610373000	0.074648000
6	-5.081224000	-0.597506000	-0.051816000
1	-6.164560000	-0.538406000	-0.043145000
6	-4.423364000	-1.770216000	-0.180663000
1	-4.950024000	-2.709806000	-0.282691000
6	-5.161357000	1.868822000	0.215484000
1	-6.230624000	1.655264000	0.155280000
1	-4.932447000	2.336787000	1.177608000
1	-4.877775000	2.561550000	-0.581118000
8	-2.273607000	-2.792775000	-0.305635000
1	-0.276080000	2.137287000	-0.043647000
1	-1.346241000	-0.470197000	-0.008481000

Syn-I:anti-U   entry 19

7	3.821180000	-1.512283000	-0.030518000
1	4.294684000	-2.410166000	-0.030951000
6	4.559925000	-0.364853000	-0.040249000
7	4.052386000	0.836757000	-0.040644000
6	2.689945000	0.824634000	-0.030150000
7	1.879008000	1.934315000	-0.027932000
6	0.584786000	1.467789000	-0.016399000
7	0.517618000	0.150264000	-0.011151000
6	1.828776000	-0.275731000	-0.019590000
6	2.388561000	-1.606714000	-0.019235000
8	1.849941000	-2.700333000	-0.011316000
6	2.321435000	3.320150000	-0.036364000
1	2.930375000	3.526445000	0.847851000
1	1.439960000	3.963852000	-0.031655000
1	2.916264000	3.520141000	-0.931566000
6	-2.984016000	-1.814306000	0.024287000
7	-2.388429000	-0.536451000	0.014337000
6	-3.033676000	0.676732000	0.013604000
8	-2.454288000	1.763359000	0.004906000
7	-4.428599000	0.615202000	0.023562000
6	-5.088740000	-0.593871000	0.033564000
1	-6.171454000	-0.524735000	0.040821000
6	-4.441305000	-1.779256000	0.034294000
1	-4.976162000	-2.719634000	0.042190000
6	-5.148076000	1.887784000	0.023319000
1	-6.219243000	1.678767000	0.030282000
1	-4.881633000	2.474502000	0.906248000
1	-4.891911000	2.468192000	-0.866762000
8	-2.299820000	-2.826962000	0.024134000
1	-0.280295000	2.118016000	-0.012115000
1	-1.354850000	-0.495942000	0.006700000
1	5.639101000	-0.490766000	-0.048118000

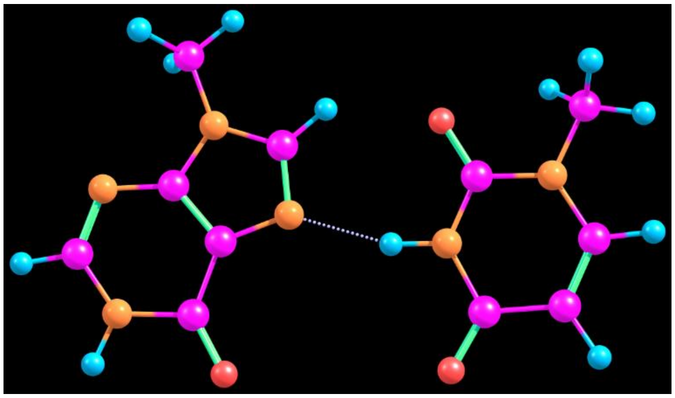

Syn-8oxoG:anti-U   entry 20

7	-4.485371000	-1.351440000	-0.009948000
1	-5.104093000	-2.151514000	-0.090891000
6	-4.994915000	-0.082085000	-0.002461000
7	-4.242060000	0.995759000	0.011453000
6	-2.913296000	0.729192000	0.001788000
7	-6.365688000	0.047627000	-0.068953000
1	-6.681671000	0.994655000	0.103271000
1	-6.925268000	-0.657034000	0.395758000
7	-1.915368000	1.677591000	0.002085000
6	-0.665860000	1.035773000	0.004377000
7	-0.930240000	-0.308880000	0.005699000
6	-2.306375000	-0.516371000	0.005423000
6	-3.092633000	-1.700262000	-0.005418000
8	-2.759287000	-2.881481000	-0.016967000
6	-2.085121000	3.118475000	-0.001818000
1	-2.638825000	3.433256000	-0.891159000
1	-1.087697000	3.560729000	-0.008381000
1	-2.629500000	3.439811000	0.891054000
6	2.632513000	-1.270394000	0.010891000
7	2.833776000	0.107755000	0.005252000
6	4.047962000	0.769067000	-0.002367000
8	4.159312000	1.985405000	-0.007073000
7	5.174789000	-0.067114000	-0.004337000
6	5.049300000	-1.432615000	0.001033000
1	5.984945000	-1.982026000	-0.001266000
6	3.848523000	-2.058814000	0.008702000
1	3.765648000	-3.137144000	0.012775000
6	6.479566000	0.594100000	-0.013082000
1	7.256493000	-0.172331000	-0.013576000
1	6.577299000	1.221946000	-0.902213000
1	6.585487000	1.229147000	0.869961000
8	0.431134000	1.607484000	0.005389000
8	1.492857000	-1.750632000	0.017257000
1	1.990524000	0.711494000	0.005792000
1	-0.172324000	-1.002674000	0.008872000

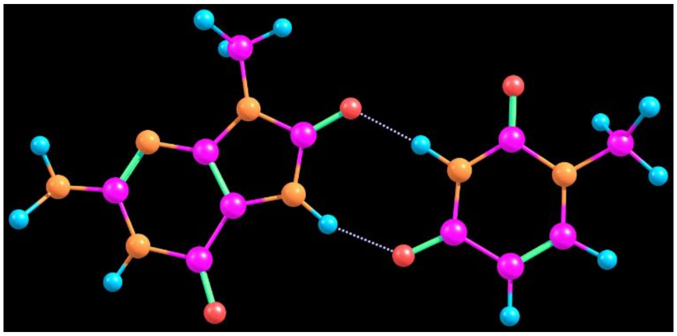

Syn-8oxoI:anti-U   entry 21

7	-4.807663000	-1.354914000	0.000849000
1	-5.435923000	-2.152262000	0.001127000
6	-5.319822000	-0.091635000	0.001058000
7	-4.591798000	0.993502000	0.000711000
6	-3.256669000	0.743836000	0.000104000
7	-2.262422000	1.697963000	-0.000356000
6	-1.013730000	1.061634000	-0.000886000
7	-1.276114000	-0.289524000	-0.000908000
6	-2.643875000	-0.499933000	-0.000180000
6	-3.420525000	-1.698558000	0.000144000
8	-3.062595000	-2.871987000	-0.000051000
6	-2.439023000	3.139127000	-0.000074000
1	-2.990856000	3.453520000	-0.890386000
1	-1.443821000	3.586134000	-0.001281000
1	-2.988616000	3.453496000	0.891644000
6	2.275124000	-1.262647000	-0.000523000
7	2.488587000	0.113318000	-0.000363000
6	3.708889000	0.764496000	0.000181000
8	3.829450000	1.979708000	0.000442000
7	4.827749000	-0.081685000	0.000422000
6	4.689835000	-1.445851000	0.000249000
1	5.620420000	-2.003717000	0.000505000
6	3.483435000	-2.061659000	-0.000214000
1	3.391112000	-3.139207000	-0.000361000
6	6.138695000	0.567935000	0.001176000
1	6.908628000	-0.205432000	0.000254000
1	6.245876000	1.199341000	-0.884262000
1	6.245970000	1.197308000	0.888070000
8	0.083674000	1.628366000	-0.001248000
8	1.130616000	-1.732596000	-0.000894000
1	1.652129000	0.724264000	-0.000663000
1	-0.513588000	-0.981142000	-0.000902000
1	-6.402865000	-0.011316000	0.001565000

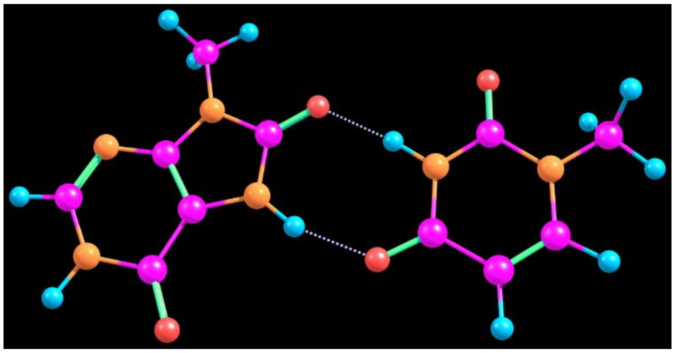

Anti-G:anti-A   entry 22

6	2.109950000	-0.718080000	-0.006340000
6	3.522220000	-0.654260000	0.052440000
6	4.105630000	0.605230000	-0.114130000
6	2.141470000	1.589410000	-0.359500000
7	3.461160000	1.766040000	-0.327940000
7	1.441080000	0.449360000	-0.201530000
6	-2.129400000	1.532910000	0.207950000
6	-3.509820000	-0.785010000	-0.226690000
7	4.488430000	-1.627510000	0.231730000
7	5.464630000	0.391320000	-0.034380000
6	5.623570000	-0.965540000	0.177640000
7	-4.500170000	-1.734980000	-0.396690000
6	6.497350000	1.412330000	-0.107660000
1	7.366840000	1.019350000	-0.640550000
1	6.092570000	2.267750000	-0.651020000
1	6.798410000	1.737470000	0.894450000
6	-2.081840000	-0.899020000	-0.236700000
6	-4.072000000	0.476350000	0.000050000
7	-1.469700000	0.359650000	-0.027710000
7	-3.440300000	1.652410000	0.236630000
7	-5.430770000	0.285920000	-0.031410000
6	-5.621200000	-1.065980000	-0.280550000
8	-1.376070000	-1.901240000	-0.397220000
1	-0.433710000	0.379210000	-0.107980000
7	-1.341070000	2.649950000	0.378480000
1	-1.845130000	3.447800000	0.744740000
1	-0.429480000	2.505630000	0.795090000
6	-6.444200000	1.317660000	0.105820000
1	-6.707090000	1.742930000	-0.869010000
1	-6.050970000	2.111980000	0.743920000
7	1.426720000	-1.863020000	0.129990000
1	1.949520000	-2.723460000	0.216230000
1	0.415680000	-1.908500000	-0.056180000
1	-6.616040000	-1.485650000	-0.356610000
1	-7.336610000	0.888400000	0.567680000
1	6.607770000	-1.405010000	0.278080000
1	1.544390000	2.480160000	-0.544490000

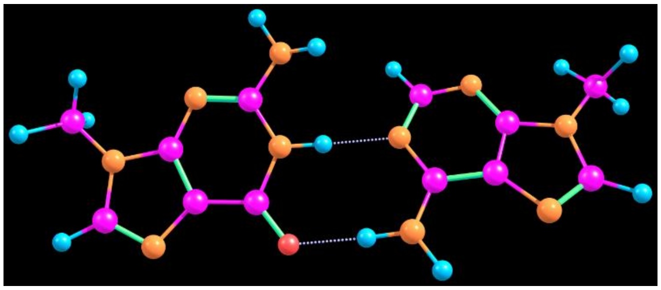

Anti-I:anti-A   entry 23

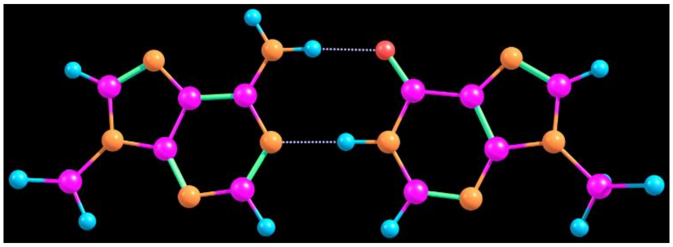

6	2.109540000	-0.593580000	-0.046620000
6	3.518970000	-0.470060000	-0.070500000
6	4.039310000	0.827170000	-0.078980000
6	2.025810000	1.738820000	-0.044490000
7	3.336130000	1.973360000	-0.066700000
7	1.383050000	0.554930000	-0.033910000
6	-2.205220000	1.606050000	0.026960000
6	-3.524500000	-0.768290000	0.048650000
7	4.533090000	-1.410490000	-0.088080000
7	5.408700000	0.670890000	-0.102430000
6	5.635340000	-0.692930000	-0.106660000
7	-4.487640000	-1.755080000	0.064710000
6	6.392270000	1.742360000	-0.118410000
1	7.008270000	1.685000000	-1.020970000
1	5.849520000	2.689170000	-0.112560000
1	7.033670000	1.688650000	0.766550000
6	-2.089770000	-0.855180000	0.024410000
6	-4.120130000	0.498320000	0.059050000
7	-1.513320000	0.436080000	0.014950000
7	-3.509640000	1.712120000	0.049050000
7	-5.475220000	0.271810000	0.081930000
6	-5.627890000	-1.102150000	0.084180000
8	-1.372420000	-1.860430000	0.012090000
1	-0.471120000	0.474940000	-0.002640000
6	-6.516740000	1.287630000	0.099820000
1	-7.155770000	1.195330000	-0.783520000
1	-7.126390000	1.193890000	1.003530000
1	-6.030790000	2.264500000	0.092610000
7	1.484420000	-1.778200000	-0.036460000
1	2.042060000	-2.620400000	-0.045980000
1	0.459450000	-1.845900000	-0.018950000
1	-6.609640000	-1.557930000	0.100680000
1	6.640590000	-1.094350000	-0.123950000
1	-1.600770000	2.508760000	0.016970000
1	1.383620000	2.616940000	-0.033320000

Anti-8oxoG:anti-A   entry 24

7	1.099164000	0.348985000	-0.003048000
1	0.059623000	0.325577000	-0.076458000
6	1.730371000	1.552502000	0.095537000
7	3.043352000	1.702769000	0.091880000
6	3.713860000	0.536993000	-0.029907000
7	5.081553000	0.398041000	-0.064176000
7	0.926138000	2.667167000	0.157189000
1	1.419983000	3.509717000	0.423963000
1	0.018998000	2.556598000	0.593265000
6	5.424853000	-0.969311000	-0.172161000
7	4.213054000	-1.637465000	-0.197329000
6	3.157508000	-0.730755000	-0.115996000
6	1.756798000	-0.897879000	-0.092127000
8	1.103895000	-1.958132000	-0.139809000
6	6.061579000	1.465534000	0.001343000
1	5.963793000	2.015591000	0.941859000
1	7.047409000	1.002319000	-0.057584000
1	5.924915000	2.159486000	-0.834141000
1	-0.688139000	-1.981797000	0.250702000
7	-1.695148000	-1.938897000	0.456401000
1	-2.191836000	-2.795947000	0.656785000
6	-2.415738000	-0.840929000	0.184035000
7	-1.785905000	0.310063000	-0.169799000
6	-2.521400000	1.401790000	-0.461774000
1	-1.952754000	2.277843000	-0.767099000
7	-3.845155000	1.542237000	-0.434013000
6	-4.450723000	0.399951000	-0.061905000
7	-5.800433000	0.162268000	0.071814000
6	-3.827998000	-0.810841000	0.256413000
7	-4.762025000	-1.778587000	0.578260000
6	-5.916380000	-1.160550000	0.457756000
6	-6.865099000	1.125147000	-0.157169000
1	-7.825302000	0.628866000	-0.002075000
1	-6.812386000	1.506069000	-1.180671000
1	-6.771952000	1.964288000	0.537716000
8	6.554595000	-1.433248000	-0.228472000
1	-6.886387000	-1.607591000	0.633617000
1	4.136623000	-2.640299000	-0.280485000

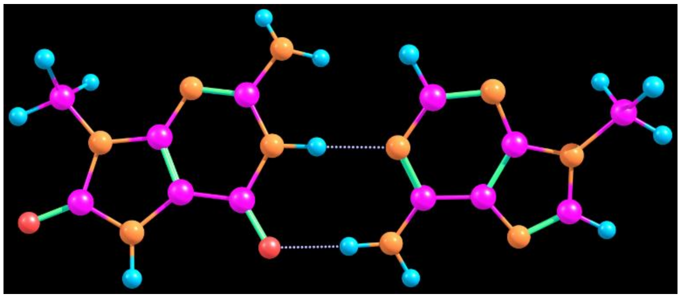

Anti-8oxoI:anti-A   entry 25

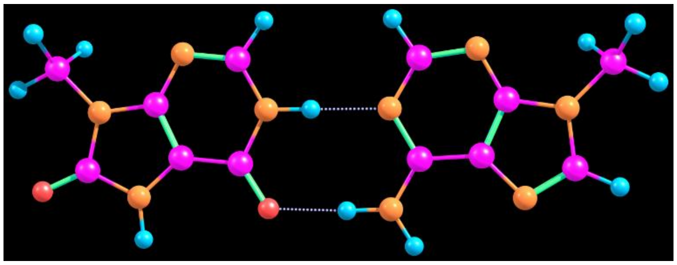

7	1.118782000	0.496860000	0.001631000
1	0.072257000	0.466114000	0.001906000
6	1.741347000	1.701856000	0.001223000
7	3.042440000	1.879701000	0.000531000
6	3.735378000	0.716566000	0.000261000
7	5.105627000	0.586583000	-0.000535000
6	5.456641000	-0.779915000	-0.000845000
7	4.242630000	-1.457135000	0.000140000
6	3.187844000	-0.557692000	0.000718000
6	1.782945000	-0.745075000	0.001363000
8	1.159348000	-1.822159000	0.001635000
6	6.078133000	1.664198000	-0.002246000
1	5.955349000	2.287884000	0.887784000
1	7.067986000	1.205266000	-0.001630000
1	5.955311000	2.285166000	-0.894198000
1	-0.676490000	-1.928441000	0.001373000
7	-1.703446000	-1.925329000	0.001305000
1	-2.207176000	-2.800939000	0.000152000
6	-2.404239000	-0.783067000	0.000772000
7	-1.755128000	0.410752000	0.001399000
6	-2.473846000	1.551407000	0.001013000
1	-1.889726000	2.469171000	0.001466000
7	-3.796390000	1.699363000	0.000158000
6	-4.422141000	0.508635000	-0.000491000
7	-5.777262000	0.263891000	-0.001869000
6	-3.818485000	-0.752262000	-0.000223000
7	-4.768866000	-1.757044000	-0.001200000
6	-5.914778000	-1.111893000	-0.002025000
6	-6.827343000	1.269082000	-0.000323000
1	-7.795422000	0.764145000	-0.016467000
1	-6.735497000	1.910004000	-0.881409000
1	-6.754431000	1.889484000	0.897234000
8	6.584696000	-1.247008000	-0.001741000
1	-6.893134000	-1.575211000	-0.003062000
1	4.171432000	-2.464383000	0.000110000
1	1.089739000	2.570533000	0.001479000

Anti-8BrG:anti-A   entry 26

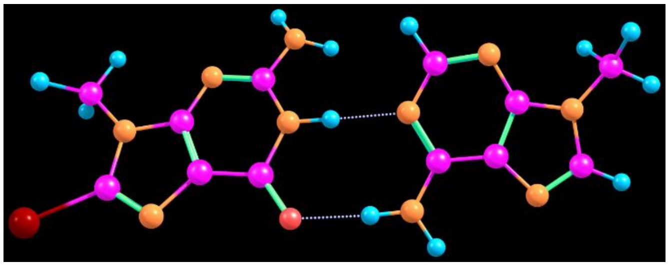

7	0.132634000	-0.600629000	0.057342000
1	-0.900606000	-0.494937000	0.120528000
6	0.653351000	-1.854725000	-0.090825000
7	1.943066000	-2.126041000	-0.091407000
6	2.702906000	-1.019408000	0.078584000
7	4.078306000	-0.987901000	0.131901000
7	-0.255157000	-2.881907000	-0.202379000
1	0.153182000	-3.756485000	-0.507122000
1	-1.146338000	-2.662445000	-0.629943000
6	4.403707000	0.350598000	0.292655000
7	3.380508000	1.151306000	0.343083000
6	2.288549000	0.309253000	0.211049000
6	0.884364000	0.590481000	0.191801000
8	0.301446000	1.676641000	0.273952000
6	4.975273000	-2.127577000	0.038929000
1	4.358961000	-3.019134000	-0.085227000
1	5.642818000	-2.016458000	-0.819910000
1	5.573468000	-2.215317000	0.949971000
1	-1.476975000	1.862964000	-0.132429000
7	-2.480339000	1.909102000	-0.353284000
1	-2.902871000	2.810072000	-0.529472000
6	-3.289180000	0.861559000	-0.138765000
7	-2.758257000	-0.348711000	0.179752000
6	-3.583033000	-1.388345000	0.415356000
1	-3.091365000	-2.317557000	0.696850000
7	-4.912950000	-1.423620000	0.358176000
6	-5.419656000	-0.224004000	0.021208000
7	-6.743659000	0.124360000	-0.127199000
6	-4.697941000	0.945484000	-0.237272000
7	-5.546854000	1.995395000	-0.537046000
6	-6.748105000	1.465970000	-0.461996000
6	-7.884579000	-0.759512000	0.045340000
1	-8.799834000	-0.183822000	-0.107506000
1	-7.883122000	-1.181395000	1.054066000
1	-7.842844000	-1.577142000	-0.679777000
35	6.201128000	0.871211000	0.419501000
1	-7.676426000	1.994548000	-0.637202000

Anti-8BrI:anti-A entry 27

6	2.106321000	-0.600696000	-0.047220000
6	3.514981000	-0.471731000	-0.070545000
6	4.030614000	0.827405000	-0.079050000
6	2.013902000	1.731870000	-0.045602000
7	3.322976000	1.970929000	-0.067301000
7	1.375613000	0.545079000	-0.035031000
6	-2.196094000	1.596121000	0.026467000
6	-3.521167000	-0.770840000	0.048347000
7	4.532271000	-1.408466000	-0.087439000
7	5.400364000	0.676128000	-0.101827000
6	5.631912000	-0.686873000	-0.105679000
7	-4.485989000	-1.759108000	0.064516000
6	6.380355000	1.751105000	-0.117453000
1	6.996945000	1.695494000	-1.019667000
1	5.834442000	2.696085000	-0.112109000
1	7.021310000	1.699585000	0.767897000
6	-2.087008000	-0.864488000	0.023681000
6	-4.113074000	0.495734000	0.059028000
7	-1.507732000	0.425226000	0.014189000
7	-3.501082000	1.706365000	0.048982000
7	-5.472486000	0.274092000	0.082318000
6	-5.611524000	-1.100823000	0.084158000
8	-1.375762000	-1.872979000	0.011092000
1	-0.463690000	0.462061000	-0.003779000
6	-6.516082000	1.287152000	0.100725000
1	-7.156080000	1.186446000	-0.779772000
1	-7.125192000	1.185866000	1.002804000
1	-6.025615000	2.261482000	0.092547000
7	1.486420000	-1.788263000	-0.037101000
1	2.047896000	-2.627951000	-0.046096000
1	0.462469000	-1.860947000	-0.019853000
1	6.638582000	-1.084694000	-0.122434000
1	-1.589831000	2.497509000	0.016382000
1	1.368421000	2.607565000	-0.034890000
35	-7.318131000	-1.872633000	0.113296000

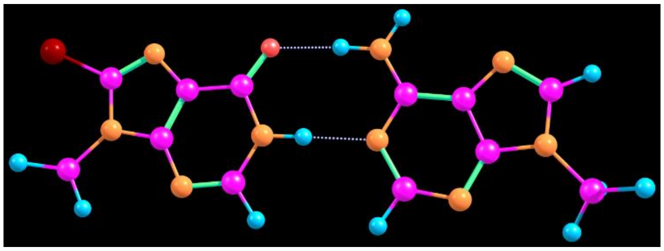

Syn-G:anti-A   entry 28

7	-4.947892000	1.418772000	-0.000918000
1	-5.536678000	2.241796000	0.072883000
6	-5.514835000	0.169288000	-0.012208000
7	-4.816525000	-0.941793000	-0.019041000
6	-3.475337000	-0.722982000	-0.000310000
7	-2.505170000	-1.695156000	0.002036000
7	-6.890945000	0.110278000	0.041702000
1	-7.255444000	-0.818447000	-0.133215000
1	-7.409865000	0.844438000	-0.424312000
6	-1.290354000	-1.035418000	0.007179000
1	-0.331982000	-1.542269000	0.010251000
7	-1.427498000	0.272139000	0.004385000
6	-2.790554000	0.493774000	-0.000675000
6	-3.543068000	1.716206000	0.002360000
8	-3.178148000	2.883194000	0.014318000
6	-2.730330000	-3.131069000	0.027817000
1	-3.427409000	-3.412618000	-0.765321000
1	-1.774625000	-3.634276000	-0.131172000
1	-3.146990000	-3.437121000	0.992137000
1	0.343861000	1.099588000	0.013427000
7	1.339699000	1.344209000	0.019028000
1	1.614331000	2.316177000	0.013954000
6	2.296768000	0.402415000	0.008438000
7	1.956101000	-0.910662000	0.004444000
6	2.933363000	-1.832918000	-0.004201000
1	2.595718000	-2.867465000	-0.006903000
7	4.258295000	-1.655400000	-0.009880000
6	4.570970000	-0.348903000	-0.005940000
7	5.826126000	0.222855000	-0.009900000
6	3.675696000	0.724826000	0.002972000
7	4.351944000	1.933619000	0.004831000
6	5.620985000	1.590239000	-0.002696000
6	7.089188000	-0.493381000	-0.015618000
1	7.904910000	0.232975000	-0.025663000
1	7.157861000	-1.130612000	-0.902037000
1	7.171802000	-1.121849000	0.876018000
1	6.455798000	2.279648000	-0.004113000

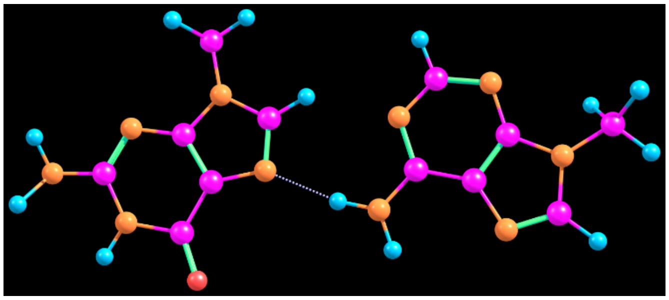

Syn-I:anti-A   entry 29

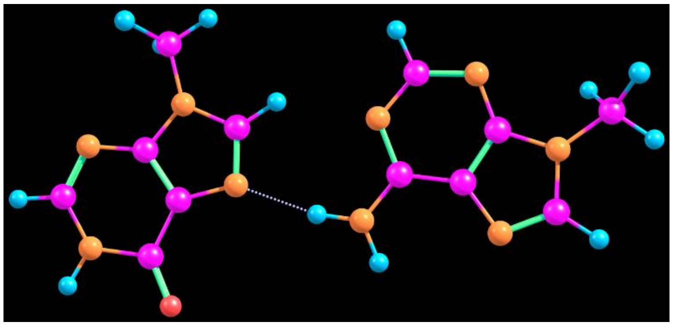

7	5.309765000	-1.418046000	-0.001169000
1	5.919207000	-2.229807000	-0.001749000
6	5.860196000	-0.167324000	-0.001316000
7	5.171849000	0.940528000	-0.000808000
6	3.827995000	0.717833000	-0.000089000
7	2.849296000	1.683092000	0.000375000
6	1.645571000	1.014145000	0.001136000
1	0.680340000	1.508818000	0.001576000
7	1.793044000	-0.296132000	0.001102000
6	3.153332000	-0.505726000	0.000317000
6	3.912379000	-1.730960000	-0.000330000
8	3.543076000	-2.896046000	-0.000331000
6	3.063285000	3.122335000	0.000126000
1	3.624333000	3.419532000	-0.889837000
1	2.089732000	3.615645000	-0.000077000
1	3.624193000	3.419942000	0.890051000
1	0.011909000	-1.133690000	0.002125000
7	-0.985043000	-1.368958000	0.001550000
1	-1.268651000	-2.338298000	0.002700000
6	-1.933161000	-0.417393000	0.001337000
7	-1.579789000	0.892166000	0.001164000
6	-2.548499000	1.824079000	0.000276000
1	-2.200994000	2.855329000	0.000181000
7	-3.874535000	1.658722000	-0.000524000
6	-4.199867000	0.355303000	-0.000242000
7	-5.460095000	-0.204576000	-0.001202000
6	-3.314630000	-0.726829000	0.000848000
7	-4.001990000	-1.929120000	0.000628000
6	-5.267802000	-1.573823000	-0.000439000
6	-6.716667000	0.523273000	-0.001793000
1	-7.538822000	-0.195774000	-0.009345000
1	-6.782706000	1.161588000	-0.887492000
1	-6.790189000	1.151480000	0.890593000
1	-6.108988000	-2.255424000	-0.000946000
1	6.945723000	-0.124177000	-0.001915000

Syn-8oxoG:anti-A   entry 30

7	-3.595970000	-0.370928000	0.379164000
1	-3.954549000	0.565754000	0.532200000
6	-4.448014000	-1.442265000	0.349235000
7	-4.035949000	-2.686574000	0.245259000
6	-2.688972000	-2.814728000	0.184987000
7	-2.000374000	-3.994392000	0.084193000
7	-5.793636000	-1.179217000	0.494397000
1	-6.374133000	-1.988952000	0.310142000
1	-6.150179000	-0.325422000	0.082193000
6	-0.603934000	-3.726189000	0.031140000
7	-0.493164000	-2.356479000	0.102558000
6	-1.754215000	-1.785399000	0.196219000
6	-2.175410000	-0.443853000	0.302472000
8	-1.511743000	0.604688000	0.341351000
6	-2.561826000	-5.329963000	0.037402000
1	-3.215615000	-5.440609000	-0.833076000
1	-1.724651000	-6.026118000	-0.036976000
1	-3.137032000	-5.535460000	0.945197000
1	0.360166000	0.872377000	0.267547000
7	1.346842000	1.141224000	0.235048000
1	1.591230000	2.119605000	0.287853000
6	2.334457000	0.236552000	0.130605000
7	2.038447000	-1.082563000	0.060543000
6	3.031908000	-1.988684000	-0.044000000
1	2.706552000	-3.025474000	-0.094102000
7	4.345371000	-1.763319000	-0.091909000
6	4.621311000	-0.450278000	-0.021589000
7	5.859404000	0.155199000	-0.041610000
6	3.700942000	0.597273000	0.088571000
7	4.345369000	1.821610000	0.136102000
6	5.621331000	1.513274000	0.056219000
6	7.138720000	-0.525847000	-0.143951000
1	7.935168000	0.221484000	-0.141286000
1	7.183949000	-1.104661000	-1.070654000
1	7.272783000	-1.205635000	0.702163000
1	6.437858000	2.224105000	0.062908000
8	0.281880000	-4.567369000	-0.059755000
1	0.417286000	-1.849768000	0.089993000

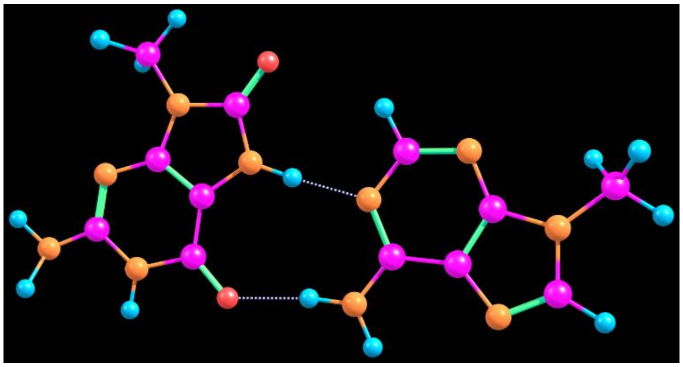

Syn-8oxoI:anti-A entry 31

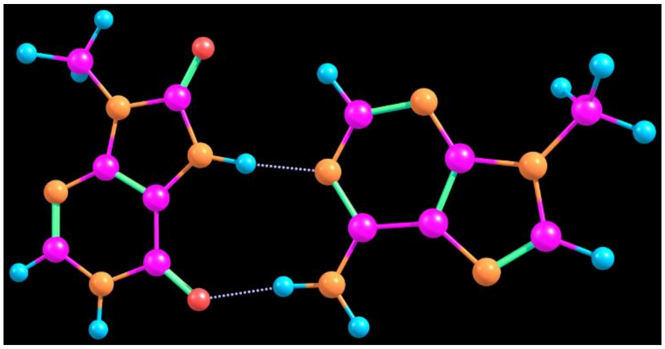

7	3.867314000	-2.340847000	-0.000409000
1	3.920310000	-3.354594000	-0.000151000
6	5.015634000	-1.602978000	-0.000778000
7	5.044204000	-0.296050000	-0.000922000
6	3.812807000	0.273782000	-0.000572000
7	3.541233000	1.618115000	-0.000855000
6	2.136531000	1.815132000	-0.000038000
7	1.589161000	0.545482000	0.000221000
6	2.594497000	-0.398382000	-0.000006000
6	2.551252000	-1.815753000	0.000202000
8	1.572810000	-2.578480000	0.000762000
6	4.504864000	2.702458000	-0.000709000
1	5.141737000	2.647097000	-0.888363000
1	3.937507000	3.634566000	-0.007810000
1	5.132685000	2.655710000	0.893956000
1	-0.290217000	-2.219706000	0.001318000
7	-1.309542000	-2.138904000	0.001168000
1	-1.869556000	-2.979243000	0.000060000
6	-1.933622000	-0.949261000	0.001202000
7	-1.209034000	0.194448000	0.002225000
6	-1.838193000	1.388140000	0.002273000
1	-1.181321000	2.255154000	0.003080000
7	-3.150493000	1.622030000	0.001364000
6	-3.854266000	0.477414000	0.000310000
7	-5.224219000	0.327702000	-0.001003000
6	-3.341673000	-0.824085000	0.000222000
7	-4.361731000	-1.759708000	-0.001058000
6	-5.458844000	-1.034513000	-0.001768000
6	-6.198863000	1.405490000	-0.001526000
1	-7.201212000	0.971855000	-0.002565000
1	-6.069816000	2.029491000	-0.890237000
1	-6.071414000	2.028950000	0.887793000
1	-6.467699000	-1.427205000	-0.002875000
8	1.560279000	2.894427000	0.000192000
1	0.559560000	0.362661000	0.001210000
1	5.945724000	-2.162859000	-0.001039000

Syn-8BrG:anti-A   entry 32

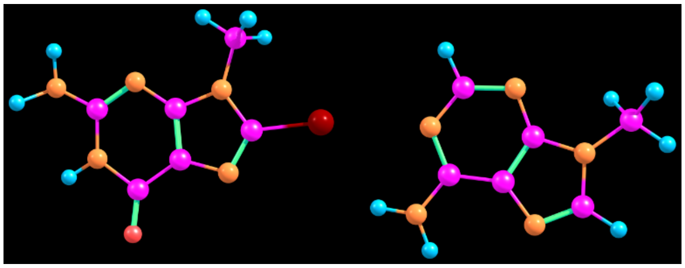

7	6.228266000	0.980129000	-0.020549000
1	7.060087000	1.555208000	-0.100555000
6	6.336481000	-0.386299000	-0.032842000
7	5.301586000	-1.193019000	-0.021575000
6	4.116023000	-0.530903000	-0.010763000
7	2.872477000	-1.119187000	-0.005866000
7	7.609193000	-0.910028000	-0.114959000
1	7.637235000	-1.910139000	0.043771000
1	8.352530000	-0.405298000	0.352056000
6	1.967643000	-0.068864000	0.022636000
7	2.524927000	1.110499000	0.035551000
6	3.883253000	0.845944000	0.015304000
6	5.007595000	1.740176000	0.006201000
8	5.067727000	2.960418000	0.010010000
6	2.586893000	-2.544387000	-0.028499000
1	3.541330000	-3.071786000	-0.059319000
1	2.032041000	-2.833513000	0.868322000
1	1.995193000	-2.797369000	-0.912468000
1	-1.559523000	1.764855000	0.013579000
7	-2.564893000	1.867266000	-0.000794000
1	-2.977126000	2.788327000	-0.011722000
6	-3.354679000	0.772147000	0.003244000
7	-2.787581000	-0.452648000	0.022577000
6	-3.580436000	-1.538288000	0.028847000
1	-3.061106000	-2.493373000	0.046044000
7	-4.914762000	-1.591852000	0.016286000
6	-5.454439000	-0.363335000	-0.005271000
7	-6.789803000	-0.021955000	-0.024433000
6	-4.763304000	0.853998000	-0.012544000
7	-5.641679000	1.923730000	-0.034796000
6	-6.829424000	1.359818000	-0.042807000
6	-7.907025000	-0.950813000	-0.029001000
1	-8.837010000	-0.380217000	-0.028799000
1	-7.870412000	-1.587675000	0.858341000
1	-7.868181000	-1.584604000	-0.918451000
35	0.110581000	-0.397260000	0.039519000
1	-7.772832000	1.890642000	-0.061158000

Syn-8BrI:anti-A   entry 33

7	-4.541328000	2.172897000	0.006353000
1	-4.789593000	3.157502000	0.004072000
6	-5.531008000	1.233542000	0.080971000
7	-5.327496000	-0.055705000	0.093524000
6	-4.006811000	-0.373263000	0.020868000
7	-3.480133000	-1.646144000	0.008737000
6	-2.110404000	-1.475089000	-0.072426000
7	-1.742158000	-0.218896000	-0.119379000
6	-2.918153000	0.493131000	-0.060979000
6	-3.136244000	1.916419000	-0.071756000
8	-2.336572000	2.838496000	-0.132706000
6	-4.250190000	-2.878748000	0.112713000
1	-5.152378000	-2.781588000	-0.493820000
1	-3.641275000	-3.704989000	-0.254630000
1	-4.532014000	-3.065157000	1.153450000
1	0.083451000	0.596114000	-0.164915000
7	0.983504000	1.074730000	-0.148833000
1	1.003051000	2.083861000	-0.115663000
6	2.132832000	0.379854000	-0.110038000
7	2.090965000	-0.972929000	-0.134299000
6	3.244843000	-1.655031000	-0.100484000
1	3.145551000	-2.738525000	-0.125199000
7	4.498680000	-1.187449000	-0.040435000
6	4.512355000	0.154911000	-0.014297000
7	5.608000000	0.991864000	0.050820000
6	3.401202000	1.004128000	-0.046058000
7	3.791819000	2.333403000	-0.000974000
6	5.103728000	2.279590000	0.055667000
6	6.996806000	0.575438000	0.113320000
1	7.629805000	1.465520000	0.134925000
1	7.248997000	-0.029445000	-0.762286000
1	7.174615000	-0.019837000	1.014006000
1	5.763814000	3.136743000	0.102981000
1	-6.546901000	1.615244000	0.133182000
35	-0.929776000	-2.916466000	-0.119959000

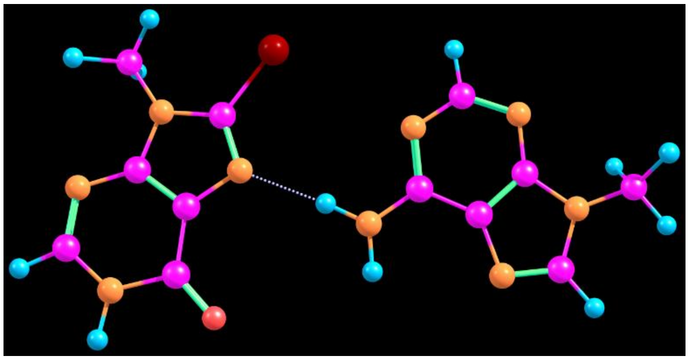

Anti-G:syn-A   entry 34

6	1.838430000	-0.896900000	-0.273440000
6	3.262100000	-0.730550000	-0.296760000
6	3.785760000	0.535680000	-0.010950000
6	1.815580000	1.507310000	0.308500000
7	3.120530000	1.673650000	0.311480000
7	1.192040000	0.326150000	0.023230000
6	-2.616220000	-1.807420000	0.382770000
6	-2.661610000	-0.421930000	0.079790000
6	-3.936340000	0.143930000	-0.040650000
6	-4.939910000	-1.758800000	0.458700000
7	-3.794860000	-2.441690000	0.584060000
7	-5.112810000	-0.469220000	0.137560000
7	-1.690380000	0.540970000	-0.163180000
7	-3.726250000	1.468180000	-0.380690000
6	-2.365430000	1.641550000	-0.435370000
6	-4.766090000	2.460530000	-0.600260000
1	-5.239960000	2.734820000	0.347360000
1	-4.319490000	3.346270000	-1.057980000
1	-5.526460000	2.046760000	-1.266900000
7	4.280230000	-1.631280000	-0.548620000
7	5.148800000	0.398270000	-0.089210000
6	5.380060000	-0.929230000	-0.422210000
1	0.157960000	0.322780000	-0.047170000
7	0.986850000	2.582370000	0.564520000
6	6.127820000	1.457050000	0.086300000
1	7.083110000	1.015290000	0.378100000
1	6.258300000	2.028060000	-0.839440000
1	5.784510000	2.132650000	0.872330000
8	1.158720000	-1.909430000	-0.468250000
1	0.104720000	2.362990000	1.013120000
1	1.475080000	3.367220000	0.978870000
1	6.386500000	-1.306180000	-0.549240000
7	-1.478260000	-2.515440000	0.495240000
1	-1.582580000	-3.512770000	0.628580000
1	-0.575420000	-2.181860000	0.140790000
1	-5.847280000	-2.331520000	0.634280000
1	-1.921690000	2.594930000	-0.693020000

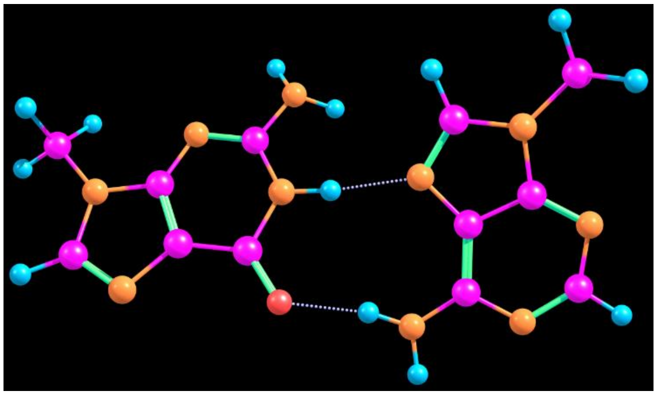

Anti-I:syn-A   entry 35

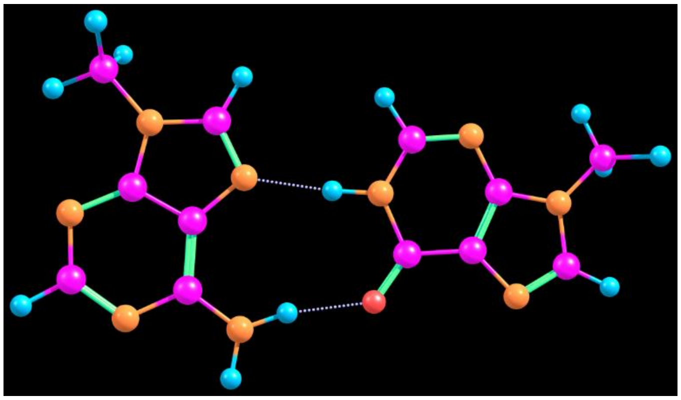

6	1.897520000	-0.788580000	0.156320000
6	3.327850000	-0.643170000	0.124980000
6	3.869530000	0.633250000	-0.065430000
6	1.909080000	1.649430000	-0.200330000
7	3.207420000	1.808480000	-0.232670000
7	1.267240000	0.464430000	-0.022530000
6	-2.654970000	-1.713840000	0.325950000
6	-2.643210000	-0.308750000	0.119530000
6	-3.894750000	0.312820000	0.037290000
6	-4.976920000	-1.591570000	0.324860000
7	-3.860660000	-2.323140000	0.424260000
7	-5.095530000	-0.271370000	0.131770000
7	-1.633960000	0.635810000	-0.026560000
7	-3.632820000	1.656780000	-0.162050000
6	-2.266410000	1.781820000	-0.190250000
6	-4.633060000	2.703510000	-0.308130000
1	-5.613340000	2.229380000	-0.236420000
1	-4.531460000	3.446550000	0.488570000
1	-4.538050000	3.192760000	-1.282140000
7	4.331610000	-1.579240000	0.254980000
7	5.233040000	0.464740000	-0.050330000
6	5.443570000	-0.887350000	0.146020000
1	0.227920000	0.463330000	-0.014900000
6	6.231450000	1.511250000	-0.209280000
1	6.861210000	1.577270000	0.683000000
1	6.857100000	1.312210000	-1.084490000
1	5.705320000	2.456710000	-0.349750000
8	1.222470000	-1.809200000	0.310460000
1	-1.789410000	2.742670000	-0.334860000
1	6.443720000	-1.298040000	0.199080000
7	-1.550940000	-2.467750000	0.428430000
1	-1.679280000	-3.459530000	0.575150000
1	-0.596430000	-2.102640000	0.367630000
1	1.266760000	2.517130000	-0.322720000
1	-5.908410000	-2.145510000	0.412970000

Anti-8oxoG:syn-A   entry 36

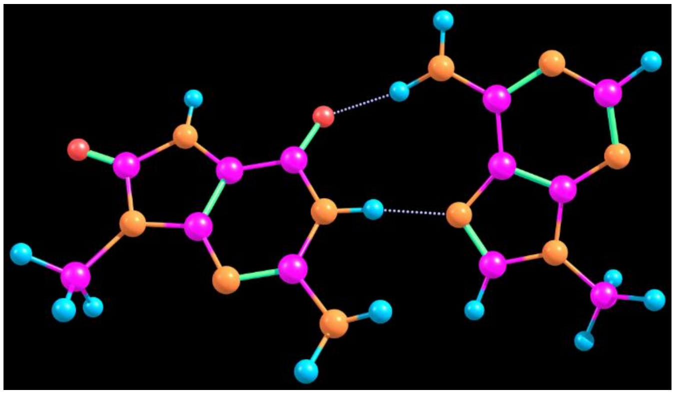

7	-0.394372000	1.145286000	-0.779567000
1	0.285252000	0.409791000	-1.055471000
6	-1.472216000	0.790284000	-0.026132000
7	-2.454145000	1.612489000	0.294789000
6	-2.294685000	2.856949000	-0.207530000
7	-3.153545000	3.914733000	-0.020877000
7	-1.532199000	-0.527724000	0.375508000
1	-2.221871000	-0.692674000	1.099222000
1	-0.642201000	-0.985396000	0.537973000
6	-2.649844000	5.065202000	-0.670102000
7	-1.454349000	4.657111000	-1.236215000
6	-1.226070000	3.309023000	-0.967612000
6	-0.162094000	2.439749000	-1.292510000
8	0.874723000	2.687783000	-1.935600000
6	-4.415703000	3.893623000	0.694211000
1	-4.307323000	3.303877000	1.607652000
1	-4.676095000	4.925871000	0.935853000
1	-5.206663000	3.455692000	0.075311000
1	4.164052000	1.999625000	-1.821529000
7	3.405420000	1.354137000	-1.644255000
1	2.451456000	1.718378000	-1.739114000
6	3.704218000	0.046471000	-1.753324000
7	4.996123000	-0.305416000	-1.946835000
6	5.315258000	-1.599719000	-2.072211000
1	6.370931000	-1.809160000	-2.223804000
7	4.504106000	-2.665697000	-2.044299000
6	3.233874000	-2.298399000	-1.836278000
7	2.129111000	-3.125425000	-1.745412000
6	2.748056000	-0.997409000	-1.660171000
7	1.374897000	-1.029205000	-1.449463000
6	1.051742000	-2.307206000	-1.514888000
6	2.143833000	-4.573203000	-1.882660000
1	1.129120000	-4.951908000	-1.736888000
1	2.811786000	-5.010338000	-1.135412000
1	2.499606000	-4.851180000	-2.879396000
8	-3.174624000	6.168414000	-0.702712000
1	0.048596000	-2.701392000	-1.411677000
1	-0.861164000	5.268972000	-1.776058000

Anti-8oxoI:syn-A   entry 37

7	0.869830000	0.550433000	0.001234000
1	-0.172936000	0.545096000	0.000710000
6	1.515106000	1.743575000	0.002719000
7	2.818606000	1.897741000	0.002911000
6	3.490294000	0.721577000	0.001337000
7	4.857694000	0.566074000	0.000903000
6	5.182889000	-0.807054000	-0.001557000
7	3.956002000	-1.461046000	-0.002104000
6	2.918732000	-0.541916000	-0.000445000
6	1.509984000	-0.703809000	-0.000705000
8	0.865861000	-1.766590000	-0.002253000
6	5.850911000	1.624674000	0.002021000
1	5.740091000	2.248484000	0.893552000
1	6.831640000	1.146517000	0.002039000
1	5.740856000	2.249876000	-0.888660000
1	-2.041042000	-3.443709000	0.002503000
7	-1.912653000	-2.441132000	0.001812000
1	-0.958877000	-2.069931000	0.000036000
6	-3.019319000	-1.682454000	0.001342000
7	-4.222170000	-2.304105000	0.002282000
6	-5.342571000	-1.571657000	0.001657000
1	-6.271069000	-2.137336000	0.002536000
7	-5.467271000	-0.237724000	0.000111000
6	-4.268303000	0.357572000	-0.000682000
7	-4.014399000	1.716460000	-0.002287000
6	-3.013158000	-0.262700000	0.000000000
7	-2.009302000	0.699740000	-0.000839000
6	-2.649726000	1.853183000	-0.002072000
6	-5.021954000	2.764864000	-0.002013000
1	-4.520956000	3.735169000	-0.010154000
1	-5.647374000	2.681765000	0.890769000
1	-5.657072000	2.671928000	-0.886867000
8	6.301718000	-1.295196000	-0.002849000
1	-2.181013000	2.828768000	-0.003136000
1	3.865726000	-2.466793000	-0.004083000
1	0.879730000	2.624219000	0.003809000

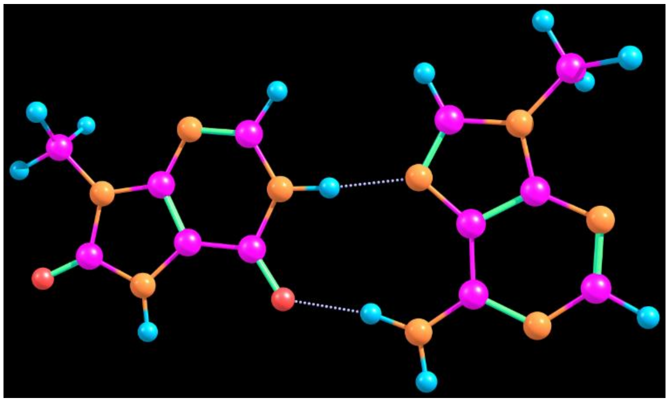

Anti-8BrG:syn-A   entry 38

7	0.132010000	0.578377000	-0.076915000
1	1.159080000	0.496704000	0.044548000
6	-0.386252000	1.801517000	-0.392386000
7	-1.674541000	2.061384000	-0.473876000
6	-2.437376000	0.976151000	-0.201228000
7	-3.813679000	0.939269000	-0.199902000
7	0.528638000	2.814247000	-0.592637000
1	0.126562000	3.634297000	-1.030349000
1	1.423274000	2.535014000	-0.978485000
6	-4.140184000	-0.372811000	0.108332000
7	-3.116530000	-1.152893000	0.294430000
6	-2.023706000	-0.323653000	0.105293000
6	-0.618211000	-0.594823000	0.171015000
8	-0.031612000	-1.656082000	0.400855000
6	-4.711937000	2.052717000	-0.456377000
1	-4.097128000	2.921205000	-0.697174000
1	-5.373011000	1.821213000	-1.295856000
1	-5.317620000	2.262922000	0.429704000
1	2.617103000	-3.465670000	-0.602102000
7	2.585748000	-2.465921000	-0.452145000
1	1.700267000	-2.064524000	-0.127009000
6	3.769259000	-1.842092000	-0.323612000
7	4.901098000	-2.559841000	-0.513767000
6	6.091299000	-1.963401000	-0.371802000
1	6.956292000	-2.600296000	-0.539203000
7	6.353811000	-0.691202000	-0.042064000
6	5.223104000	0.005334000	0.124943000
7	5.107614000	1.341464000	0.462700000
6	3.911703000	-0.464821000	-0.011638000
7	3.011212000	0.567066000	0.223137000
6	3.763177000	1.613890000	0.506987000
6	6.215683000	2.243145000	0.733796000
1	5.820285000	3.246820000	0.903478000
1	6.899028000	2.255941000	-0.118944000
1	6.764072000	1.910491000	1.619413000
1	3.389232000	2.597326000	0.762910000
35	-5.938902000	-0.888953000	0.230273000

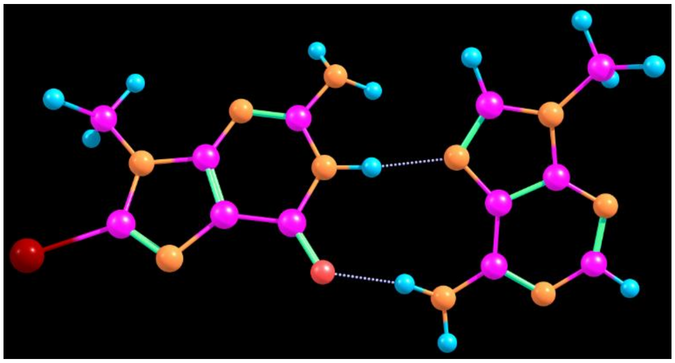

Anti-8BrI:syn-A   entry 39

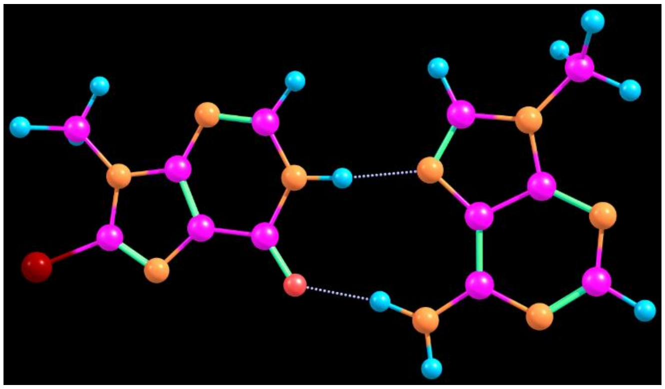

6	0.596221000	-0.473712000	-0.000202000
6	2.008726000	-0.204611000	-0.000066000
6	2.441657000	1.124595000	-0.000083000
6	0.404256000	1.981952000	-0.000390000
7	1.685496000	2.251554000	-0.000229000
7	-0.136031000	0.735624000	-0.000391000
6	-3.863247000	-1.772990000	-0.000151000
6	-3.961452000	-0.356178000	-0.000165000
6	-5.258007000	0.171484000	0.000095000
6	-6.187597000	-1.832487000	0.000335000
7	-5.017231000	-2.481620000	0.000133000
7	-6.409151000	-0.511492000	0.000353000
7	-3.030088000	0.675748000	-0.000498000
7	-5.102919000	1.546461000	-0.000079000
6	-3.751079000	1.780752000	-0.000306000
6	-6.183107000	2.521778000	0.000792000
1	-7.122697000	1.966801000	-0.003671000
1	-6.136213000	3.146648000	0.897681000
1	-6.131388000	3.153201000	-0.891193000
7	3.086924000	-1.067424000	0.000087000
7	3.818037000	1.070851000	0.000063000
6	4.124113000	-0.276844000	0.000116000
1	-1.173184000	0.648617000	-0.000509000
6	4.730585000	2.203581000	0.000046000
1	5.363133000	2.179227000	0.891258000
1	5.362576000	2.179677000	-0.891578000
1	4.125152000	3.110980000	0.000458000
8	0.012698000	-1.559263000	-0.000179000
1	-3.351545000	2.786735000	-0.000449000
7	-2.703754000	-2.446786000	-0.000510000
1	-2.754611000	-3.456279000	-0.000200000
1	-1.781129000	-2.004496000	-0.000390000
1	-0.307880000	2.802376000	-0.000532000
1	-7.072692000	-2.463979000	0.000519000
35	5.912386000	-0.833993000	0.000211000

Syn-G:syn-A   entry 40

7	3.300564000	-1.851879000	0.406255000
1	3.151750000	-2.807956000	0.711880000
6	4.466386000	-1.202720000	0.728253000
7	4.686252000	0.062189000	0.456991000
6	3.637300000	0.658714000	-0.165679000
7	3.584168000	1.969186000	-0.561261000
7	5.410708000	-1.929080000	1.419178000
1	6.304276000	-1.457810000	1.491011000
1	5.486416000	-2.918906000	1.215508000
6	2.347314000	2.133179000	-1.171136000
7	1.628624000	1.038211000	-1.187576000
6	2.417413000	0.096746000	-0.558471000
6	2.167513000	-1.279441000	-0.250864000
8	1.184030000	-1.986420000	-0.451849000
6	4.619219000	2.969547000	-0.361664000
1	5.556109000	2.639094000	-0.817641000
1	4.296729000	3.900870000	-0.831011000
1	4.785376000	3.140207000	0.705757000
1	-1.747459000	-3.126235000	-0.861277000
7	-1.728552000	-2.227534000	-0.399038000
1	-0.827549000	-1.761562000	-0.331954000
6	-2.896292000	-1.559460000	-0.308214000
7	-4.033225000	-2.195123000	-0.674244000
6	-5.201166000	-1.541403000	-0.587928000
1	-6.077014000	-2.108328000	-0.896809000
7	-5.428823000	-0.286754000	-0.179079000
6	-4.288188000	0.317294000	0.187829000
7	-4.130906000	1.604127000	0.659743000
6	-2.998946000	-0.232137000	0.171316000
7	-2.067059000	0.684904000	0.633770000
6	-2.778375000	1.753763000	0.911083000
6	-5.195966000	2.567783000	0.863985000
1	-4.752770000	3.534446000	1.116047000
1	-5.858083000	2.246586000	1.674458000
1	-5.786276000	2.666461000	-0.052025000
1	-2.383294000	2.685876000	1.296191000
1	2.050011000	3.089695000	-1.579572000

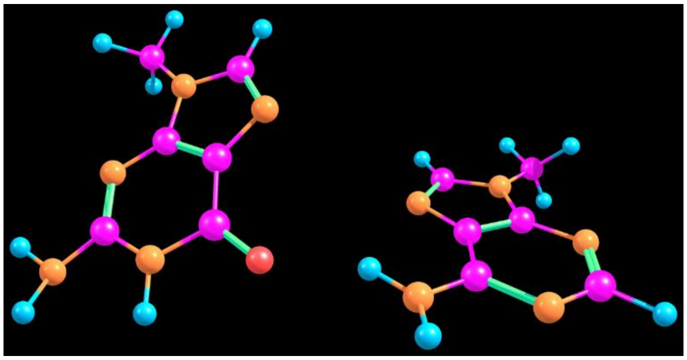

Syn-8oxoG:syn-A   entry 41

7	-3.434734000	-2.038615000	-0.004670000
1	-3.515331000	-3.046906000	-0.084080000
6	-4.557435000	-1.253523000	0.000909000
7	-4.518265000	0.061164000	0.010912000
6	-3.263531000	0.570952000	0.000350000
7	-2.936023000	1.903385000	-0.002275000
7	-5.773952000	-1.897923000	-0.064999000
1	-6.558189000	-1.277453000	0.097618000
1	-5.858720000	-2.789450000	0.408099000
6	-1.522191000	2.042409000	0.000761000
7	-1.029781000	0.759909000	0.006612000
6	-2.075842000	-0.148966000	0.007239000
6	-2.092090000	-1.559753000	0.000692000
8	-1.153477000	-2.369048000	-0.006852000
6	-3.854213000	3.025280000	-0.009466000
1	-4.487940000	3.007305000	0.882420000
1	-3.249889000	3.934013000	-0.016307000
1	-4.489214000	2.994257000	-0.899952000
1	1.921659000	-3.650521000	-0.005535000
7	1.771511000	-2.651592000	-0.000206000
1	0.811975000	-2.308524000	-0.003305000
6	2.859257000	-1.859068000	-0.000529000
7	4.081056000	-2.439655000	-0.002482000
6	5.173809000	-1.662555000	-0.002513000
1	6.122860000	-2.193200000	-0.004061000
7	5.249515000	-0.325264000	-0.001035000
6	4.029360000	0.228496000	0.000933000
7	3.713246000	1.575004000	0.002558000
6	2.802571000	-0.444908000	0.001636000
7	1.761399000	0.468526000	0.003927000
6	2.340992000	1.652714000	0.004191000
6	4.667933000	2.671068000	0.001484000
1	4.117891000	3.614209000	0.007221000
1	5.297918000	2.618799000	-0.890697000
1	5.306204000	2.612716000	0.887318000
8	-0.902897000	3.102841000	-0.001019000
1	1.806141000	2.595228000	0.005345000
1	-0.013912000	0.541735000	0.006366000

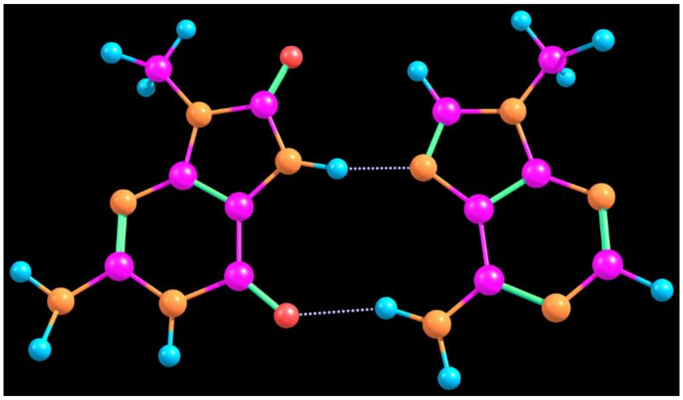

Syn-8oxoI:syn-A   entry 42

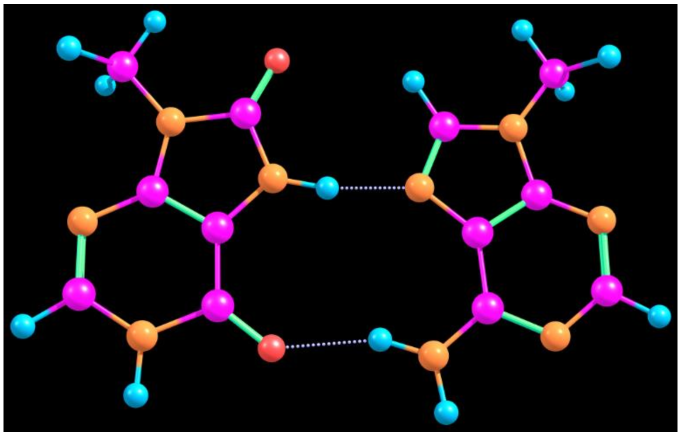

7	3.546020000	-2.413415000	-0.000331000
1	3.568371000	-3.428336000	-0.000413000
6	4.717504000	-1.711715000	-0.000590000
7	4.789562000	-0.406285000	-0.000462000
6	3.577003000	0.202886000	0.000144000
7	3.345234000	1.557018000	-0.000062000
6	1.948938000	1.793647000	0.000506000
7	1.365617000	0.542666000	0.001418000
6	2.340106000	-0.430884000	0.000940000
6	2.246160000	-1.846452000	0.000732000
8	1.241024000	-2.569882000	0.001135000
6	4.342023000	2.611272000	-0.002032000
1	4.973643000	2.537341000	-0.892062000
1	3.804506000	3.560890000	-0.005855000
1	4.971297000	2.543379000	0.890161000
1	-1.934958000	-3.614166000	0.000110000
7	-1.701860000	-2.631246000	0.000008000
1	-0.717832000	-2.368440000	0.000783000
6	-2.719039000	-1.749768000	-0.000199000
7	-3.985107000	-2.225170000	-0.000888000
6	-5.008595000	-1.358883000	-0.001237000
1	-5.998954000	-1.807590000	-0.001802000
7	-4.970950000	-0.019997000	-0.000984000
6	-3.708686000	0.428825000	-0.000279000
7	-3.280632000	1.744191000	0.000296000
6	-2.542999000	-0.345446000	0.000139000
7	-1.428866000	0.477988000	0.000898000
6	-1.907414000	1.707059000	0.001073000
6	-4.140474000	2.916423000	0.000188000
1	-3.513560000	3.810297000	0.002667000
1	-4.778612000	2.912664000	0.888003000
1	-4.775212000	2.915219000	-0.890084000
8	1.398217000	2.889544000	0.000230000
1	-1.295208000	2.601225000	0.001596000
1	0.333269000	0.399533000	0.001503000
1	5.629034000	-2.301349000	-0.001081000

Syn-8BrG:syn-A   entry 43

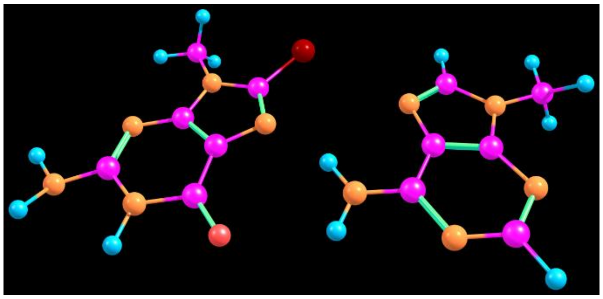

7	-4.037986000	-2.294495000	-0.017442000
1	-4.297021000	-3.272795000	-0.093157000
6	-5.007913000	-1.323274000	-0.053043000
7	-4.745083000	-0.036045000	-0.045505000
6	-3.416193000	0.232444000	-0.016534000
7	-2.845125000	1.484686000	-0.013930000
7	-6.310529000	-1.751138000	-0.154576000
1	-6.991472000	-1.014479000	-0.017135000
1	-6.559006000	-2.630258000	0.281241000
6	-1.476445000	1.268522000	0.030225000
7	-1.147073000	0.006681000	0.059040000
6	-2.352812000	-0.668035000	0.031034000
6	-2.623519000	-2.075676000	0.030667000
8	-1.862809000	-3.034414000	0.055932000
6	-3.550387000	2.756098000	-0.062277000
1	-4.618626000	2.540643000	-0.022541000
1	-3.266233000	3.375536000	0.792256000
1	-3.311256000	3.287433000	-0.987018000
1	0.706223000	-2.880675000	0.090364000
7	1.129565000	-1.962584000	0.095622000
1	0.510598000	-1.154211000	0.090029000
6	2.463674000	-1.833529000	0.044872000
7	3.239402000	-2.943692000	0.023928000
6	4.572278000	-2.802592000	-0.020266000
1	5.137703000	-3.732054000	-0.035037000
7	5.298918000	-1.676745000	-0.049172000
6	4.507643000	-0.592728000	-0.029564000
7	4.892263000	0.732776000	-0.049168000
6	3.108940000	-0.572091000	0.016076000
7	2.635102000	0.730538000	0.022667000
6	3.721600000	1.468292000	-0.017184000
6	6.257480000	1.221650000	-0.103534000
1	6.241737000	2.312756000	-0.045617000
1	6.736755000	0.912596000	-1.037898000
1	6.834966000	0.820027000	0.734398000
35	-0.284504000	2.704432000	0.046387000
1	3.743488000	2.551324000	-0.023996000

Anti-G:syn-G   entry 44

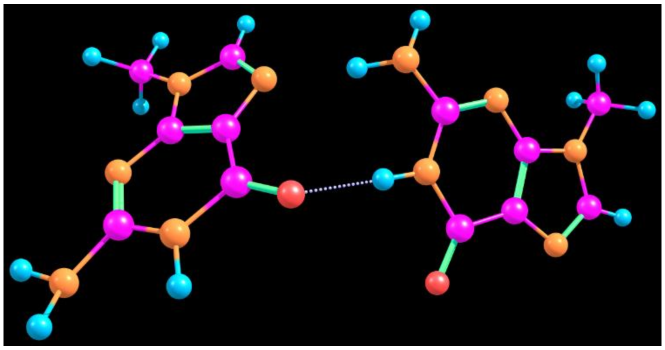

6	-2.378267000	-1.467845000	0.191671000
6	-3.722876000	-0.991719000	0.388921000
6	-4.032455000	0.313869000	-0.001169000
6	-1.988544000	0.817570000	-0.732759000
7	-3.220624000	1.256334000	-0.539257000
7	-1.577169000	-0.448816000	-0.410598000
6	2.157712000	-0.908293000	-0.729408000
6	2.559411000	0.399028000	-0.312812000
6	3.828634000	0.571255000	0.242518000
6	4.423946000	-1.568548000	0.075430000
7	3.197184000	-1.849893000	-0.478341000
7	4.793498000	-0.360665000	0.443446000
7	1.878683000	1.600413000	-0.354028000
7	3.918760000	1.906559000	0.546894000
6	2.713960000	2.472696000	0.164423000
8	1.103746000	-1.288522000	-1.231748000
1	2.934126000	-2.810019000	-0.677488000
6	5.065333000	2.570607000	1.149496000
1	5.936513000	2.487678000	0.492909000
1	4.818741000	3.623619000	1.297805000
1	5.301253000	2.112203000	2.114330000
6	-6.128664000	1.704830000	0.129300000
1	-5.706509000	2.273219000	-0.701796000
1	-7.170518000	1.458900000	-0.090083000
1	-6.084978000	2.319678000	1.036687000
7	-4.838217000	-1.618713000	0.917346000
7	-5.366336000	0.480148000	0.289662000
6	-5.788886000	-0.718515000	0.846586000
8	-1.879351000	-2.556028000	0.455793000
1	-0.634133000	-0.746220000	-0.677651000
7	5.266367000	-2.628467000	0.291980000
1	6.211635000	-2.362594000	0.540286000
1	5.197985000	-3.437414000	-0.311945000
1	-6.813678000	-0.856195000	1.167211000
7	-1.065208000	1.641346000	-1.319954000
1	-1.389090000	2.596473000	-1.402871000
1	-0.082047000	1.550389000	-1.057717000
1	2.521147000	3.529354000	0.294732000

Anti-I:syn-G   entry 45

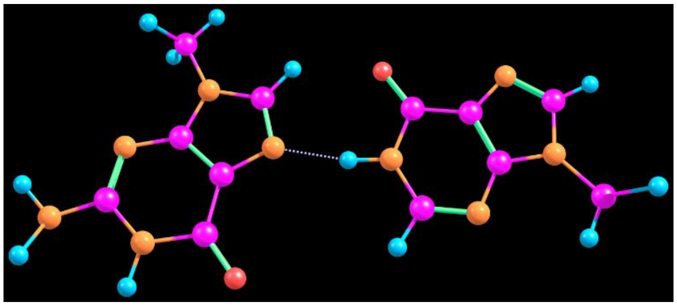

6	-2.281770000	0.976120000	-0.003290000
6	-3.706270000	0.757110000	-0.001980000
6	-4.185410000	-0.557580000	-0.000230000
6	-2.180720000	-1.495660000	-0.001040000
7	-3.472790000	-1.714850000	0.000330000
7	-1.598950000	-0.268640000	-0.002960000
6	2.894340000	-1.691690000	-0.000430000
6	2.485440000	-0.317560000	-0.006300000
6	3.456890000	0.683640000	0.000160000
6	5.195940000	-0.706600000	-0.000670000
7	4.325670000	-1.769280000	0.004270000
7	4.808340000	0.547610000	-0.010550000
7	1.225830000	0.245100000	-0.005660000
7	2.765900000	1.871520000	0.003130000
6	1.423960000	1.545950000	-0.000400000
8	2.231650000	-2.720890000	0.008200000
1	4.683040000	-2.715930000	0.080650000
6	3.350090000	3.203770000	0.009490000
1	3.966960000	3.349210000	-0.881570000
1	2.540530000	3.935650000	0.016080000
1	3.971690000	3.338300000	0.898870000
7	-4.755710000	1.653230000	-0.001860000
7	-5.556930000	-0.453430000	0.001010000
6	-5.833200000	0.900710000	-0.000080000
1	-0.563400000	-0.208750000	-0.004060000
7	6.539160000	-1.004040000	0.063560000
1	7.133580000	-0.201000000	-0.103440000
1	6.857200000	-1.849210000	-0.394450000
6	-6.500720000	-1.559640000	0.002860000
1	-7.132220000	-1.523290000	0.895950000
1	-7.131080000	-1.527460000	-0.891200000
1	-5.926440000	-2.487520000	0.005370000
8	-1.651030000	2.033420000	-0.004560000
1	0.627190000	2.280170000	0.001220000
1	-6.852260000	1.266110000	0.000590000
1	-1.494850000	-2.338510000	-0.000570000

Anti-I:syn-G # 2 entry 46

6	2.821450000	-1.679230000	-0.017650000
6	4.034300000	-0.893200000	-0.036170000
6	3.939100000	0.500430000	0.013230000
6	1.724270000	0.549290000	0.091890000
7	2.819740000	1.270330000	0.077340000
7	1.692590000	-0.810520000	0.050440000
6	-2.155900000	-1.107100000	0.070860000
6	-2.441730000	0.294990000	0.052060000
6	-3.775570000	0.707760000	-0.011380000
6	-4.637480000	-1.345780000	-0.024250000
7	-3.364910000	-1.860880000	0.024450000
7	-4.895100000	-0.057680000	-0.038710000
7	-1.595790000	1.387010000	0.080470000
7	-3.736500000	2.079520000	-0.017910000
6	-2.395640000	2.426090000	0.034750000
8	-1.084750000	-1.706870000	0.110920000
1	-3.217780000	-2.863790000	-0.031440000
6	-4.881190000	2.971980000	-0.120070000
1	-4.698510000	3.867400000	0.478580000
1	-5.060880000	3.258240000	-1.161490000
1	-5.760700000	2.451600000	0.262960000
7	5.356260000	-1.285060000	-0.098010000
7	5.235340000	0.963080000	-0.018160000
6	6.036860000	-0.160820000	-0.085830000
1	0.772300000	-1.260370000	0.068140000
7	-5.669310000	-2.247560000	-0.115350000
1	-6.585110000	-1.834690000	0.011550000
1	-5.556210000	-3.153650000	0.321120000
6	5.648010000	2.356050000	0.008770000
1	6.304470000	2.544830000	0.864520000
1	6.170520000	2.621970000	-0.916310000
1	4.748500000	2.967670000	0.102220000
8	2.672340000	-2.893950000	-0.051420000
1	-2.082070000	3.461710000	0.041240000
1	7.115910000	-0.080630000	-0.122910000
1	0.753620000	1.035810000	0.136510000

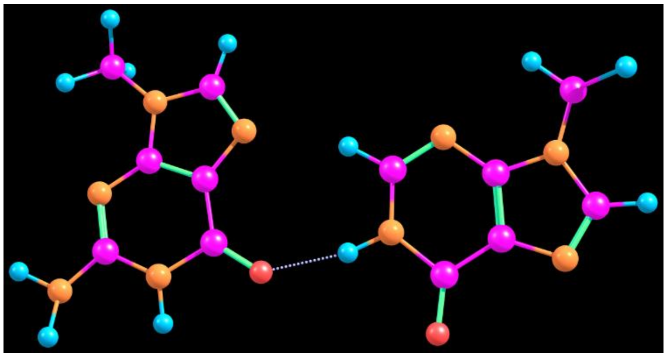

Anti-8oxoG:syn-G   entry 47

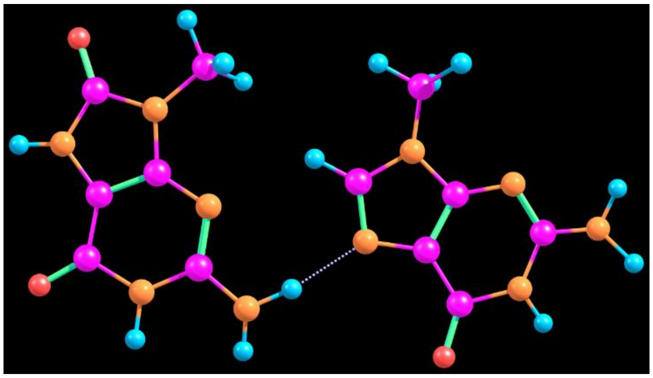

8	3.806075000	-2.804369000	-0.024784000
6	3.967157000	-1.591867000	-0.021839000
7	5.293448000	-1.052746000	-0.104790000
1	6.013002000	-1.758392000	-0.223221000
6	5.630026000	0.278564000	-0.120398000
7	4.747596000	1.249337000	-0.058586000
6	3.469129000	0.797366000	0.008238000
7	2.341986000	1.581102000	0.070292000
7	6.964428000	0.580809000	-0.263956000
1	7.174543000	1.559408000	-0.108869000
1	7.641524000	-0.054244000	0.140037000
6	1.267680000	0.714751000	0.137854000
1	0.239521000	1.046403000	0.193869000
7	1.636287000	-0.546559000	0.123998000
6	3.013917000	-0.521179000	0.043507000
6	2.308363000	3.035209000	0.058295000
1	2.738163000	3.415695000	-0.871844000
1	2.878193000	3.432503000	0.902310000
1	1.268628000	3.358462000	0.137469000
1	-0.594822000	-3.319015000	0.075761000
7	-0.653940000	-2.340566000	0.318690000
1	0.218662000	-1.795489000	0.258346000
6	-1.822575000	-1.677462000	0.158850000
7	-1.838364000	-0.348505000	0.173060000
6	-3.073292000	0.183573000	0.054687000
7	-2.971457000	-2.420335000	0.023024000
1	-2.926258000	-3.433182000	0.048452000
7	-3.357773000	1.533307000	0.047062000
6	-4.749933000	1.735368000	-0.097647000
7	-5.283636000	0.464428000	-0.174684000
6	-4.271652000	-0.494579000	-0.084058000
6	-4.300060000	-1.908848000	-0.107535000
8	-5.254970000	-2.679818000	-0.216420000
6	-2.407533000	2.618102000	0.160694000
1	-1.839780000	2.531789000	1.093220000
1	-2.977677000	3.548661000	0.161930000
1	-1.715164000	2.614634000	-0.688225000
8	-5.324285000	2.815838000	-0.141321000
1	-6.270490000	0.283664000	-0.281689000

Anti-8oxoG:syn-G #2 entry 48

6	-2.301025000	-1.420639000	0.189389000
6	-3.602321000	-0.858319000	0.222660000
6	-3.816822000	0.456885000	-0.145449000
6	-1.659056000	0.840486000	-0.599872000
7	-2.883922000	1.345272000	-0.549106000
7	-1.359499000	-0.450514000	-0.262815000
6	2.504153000	-0.970965000	-0.434808000
6	2.962828000	0.343232000	-0.110585000
6	4.310376000	0.529645000	0.202675000
6	4.888788000	-1.602722000	-0.070367000
7	3.582138000	-1.899033000	-0.381389000
7	5.305301000	-0.390979000	0.223160000
7	2.275331000	1.537935000	-0.030741000
7	4.441364000	1.867287000	0.483257000
6	3.182201000	2.420263000	0.324533000
8	1.378940000	-1.369285000	-0.723475000
1	3.299825000	-2.861440000	-0.534048000
6	5.673295000	2.545016000	0.863887000
1	6.413646000	2.459961000	0.062523000
1	5.451076000	3.598647000	1.043346000
1	6.080868000	2.098752000	1.775801000
6	-5.834811000	1.976777000	-0.258442000
1	-5.338015000	2.489097000	-1.084885000
1	-6.875788000	1.768527000	-0.510631000
1	-5.802746000	2.616425000	0.631513000
7	-4.841872000	-1.393043000	0.581479000
7	-5.166828000	0.713370000	-0.016585000
6	-5.833921000	-0.440856000	0.444822000
8	-1.944514000	-2.563947000	0.486237000
1	-0.400904000	-0.791735000	-0.387567000
7	5.769412000	-2.651603000	-0.019823000
1	6.740672000	-2.375140000	0.051550000
1	5.601269000	-3.458529000	-0.606077000
7	-0.636340000	1.628426000	-1.052544000
1	-0.901157000	2.601302000	-1.136895000
1	0.309515000	1.483629000	-0.696446000
1	3.005651000	3.474854000	0.489230000
8	-7.034917000	-0.545378000	0.664616000
1	-5.017695000	-2.338107000	0.888652000

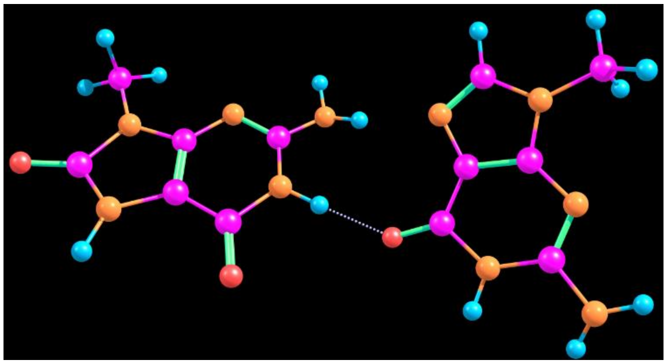

Anti-8oxoI:syn-G entry 49

6	-2.462105000	-1.640282000	0.008356000
6	-3.650669000	-0.854104000	-0.001023000
6	-3.599695000	0.527510000	0.003609000
6	-1.373040000	0.575963000	0.026111000
7	-2.477950000	1.288895000	0.016840000
7	-1.331977000	-0.783059000	0.022912000
6	2.484041000	-1.110943000	0.019515000
6	2.769767000	0.290454000	0.008836000
6	4.105557000	0.701877000	-0.006107000
6	4.966233000	-1.351434000	-0.017214000
7	3.691865000	-1.865184000	0.008153000
7	5.224913000	-0.063226000	-0.032022000
7	1.924812000	1.383722000	0.021501000
7	4.068677000	2.072668000	-0.003654000
6	2.727593000	2.421357000	0.013414000
8	1.411030000	-1.711028000	0.042693000
1	3.547259000	-2.867577000	0.078487000
6	5.220990000	2.960492000	-0.015733000
1	5.816872000	2.793146000	-0.917062000
1	4.865264000	3.992450000	-0.001897000
1	5.845601000	2.779139000	0.863041000
6	-5.315605000	2.384677000	-0.007618000
1	-4.928626000	2.899179000	-0.892327000
1	-6.406812000	2.394771000	-0.018949000
1	-4.947220000	2.892996000	0.888533000
7	-4.991399000	-1.216081000	-0.015885000
7	-4.898843000	0.996245000	-0.008109000
6	-5.798245000	-0.087070000	-0.020321000
8	-2.356722000	-2.868709000	0.004944000
1	-0.408355000	-1.230917000	0.031463000
7	6.000265000	-2.252880000	0.029680000
1	6.908562000	-1.843383000	-0.150670000
1	5.863863000	-3.166199000	-0.384392000
1	2.416913000	3.457836000	0.019256000
8	-7.021037000	-0.026896000	-0.032288000
1	-5.355043000	-2.157800000	-0.021370000
1	-0.405918000	1.070743000	0.036248000

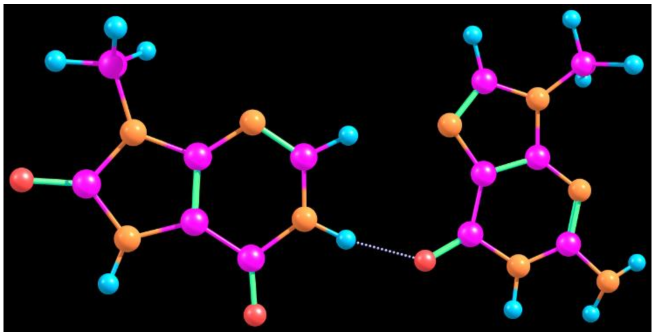

Anti-8BrG:syn-G   entry 50

8	5.025718000	2.474569000	0.113746000
6	4.981043000	1.253588000	0.071816000
7	6.199414000	0.497719000	0.115307000
1	7.028284000	1.069202000	0.242086000
6	6.309197000	-0.870305000	0.084141000
7	5.276067000	-1.677695000	0.006758000
6	4.090555000	-1.016711000	-0.023479000
7	2.847639000	-1.599573000	-0.090173000
7	7.575619000	-1.395846000	0.195187000
1	7.617858000	-2.389523000	0.004828000
1	8.345428000	-0.868344000	-0.196892000
6	1.932231000	-0.564789000	-0.112039000
1	0.862321000	-0.716279000	-0.161670000
7	2.506120000	0.616556000	-0.064969000
6	3.861243000	0.359573000	-0.009753000
6	2.572703000	-3.026760000	-0.126767000
1	2.966545000	-3.511123000	0.771333000
1	3.037408000	-3.479533000	-1.007270000
1	1.491232000	-3.170318000	-0.172453000
1	0.693558000	3.698080000	-0.009572000
7	0.497966000	2.733589000	-0.235915000
1	1.281499000	2.069298000	-0.166975000
6	-0.760952000	2.252774000	-0.120993000
7	-0.977993000	0.943752000	-0.129102000
6	-2.291030000	0.628268000	-0.054628000
7	-1.787809000	3.165587000	-0.033430000
1	-1.579021000	4.158058000	-0.060964000
7	-2.791500000	-0.656616000	-0.053353000
6	-4.171108000	-0.509210000	0.037407000
7	-4.571847000	0.725065000	0.090693000
6	-3.402944000	1.466981000	0.035470000
6	-3.192111000	2.887441000	0.049288000
8	-3.982475000	3.817976000	0.113281000
6	-1.989484000	-1.862895000	-0.121519000
1	-1.364037000	-1.839177000	-1.019104000
1	-2.652990000	-2.726269000	-0.165394000
1	-1.350848000	-1.940750000	0.764484000
35	-5.307856000	-2.004262000	0.071447000

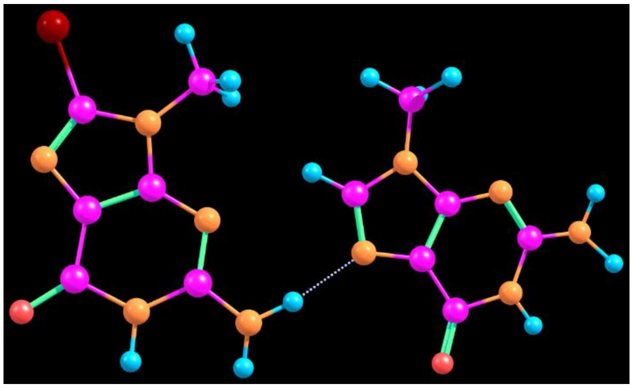

Syn-8oxoG:syn-G entry 51

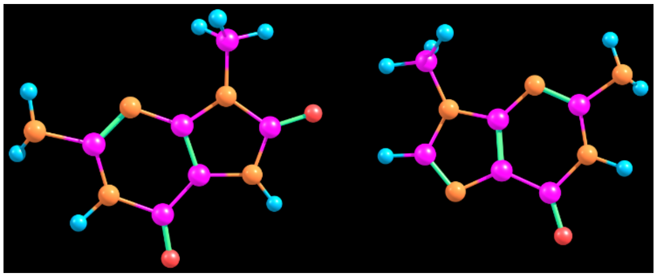

8	-4.843071000	2.988050000	0.165222000
6	-4.942103000	1.766737000	0.078924000
7	-6.227590000	1.156067000	-0.091206000
1	-6.986441000	1.821158000	-0.194415000
6	-6.465228000	-0.188174000	-0.201103000
7	-5.511628000	-1.095381000	-0.160591000
6	-4.270471000	-0.574160000	-0.015673000
7	-3.102566000	-1.301662000	0.051606000
7	-7.763565000	-0.583504000	-0.418773000
1	-7.902863000	-1.583266000	-0.334054000
1	-8.506959000	-0.026349000	-0.015995000
6	-2.007699000	-0.431975000	0.210470000
7	-2.555228000	0.834414000	0.250774000
6	-3.940238000	0.763203000	0.111722000
6	-2.969507000	-2.742389000	-0.065687000
1	-2.950028000	-3.050575000	-1.117440000
1	-3.814272000	-3.220626000	0.433976000
1	-2.031966000	-3.033107000	0.411402000
1	8.390794000	-1.092855000	0.233445000
7	7.550995000	-1.459412000	-0.198851000
1	7.383522000	-2.434041000	0.022345000
6	6.406665000	-0.690151000	-0.092041000
7	5.238451000	-1.274333000	0.014501000
6	4.204407000	-0.388638000	0.032749000
7	6.568978000	0.669629000	-0.160370000
1	7.491419000	1.062503000	-0.314926000
7	2.877665000	-0.715310000	0.124787000
6	2.183267000	0.485114000	0.123690000
7	2.969716000	1.534064000	0.039040000
6	4.246853000	1.009093000	-0.017819000
6	5.523712000	1.658393000	-0.128878000
8	5.826726000	2.841183000	-0.204471000
6	2.319062000	-2.056073000	0.204894000
1	2.592768000	-2.630094000	-0.685516000
1	1.233592000	-1.970138000	0.271444000
1	2.707620000	-2.571467000	1.088657000
1	1.103691000	0.487426000	0.188206000
8	-0.827711000	-0.760905000	0.296251000
1	-2.007348000	1.676941000	0.352943000

Syn-I:anti-G entry 52

6	2.194859000	1.429965000	0.251835000
6	3.519124000	0.887417000	0.412575000
6	3.764441000	-0.415987000	-0.027790000
6	1.691061000	-0.801529000	-0.742425000
7	2.902390000	-1.303111000	-0.584159000
7	1.341796000	0.469553000	-0.372590000
6	-2.347192000	1.214276000	-0.678752000
6	-2.832443000	-0.070748000	-0.258369000
6	-4.122170000	-0.175215000	0.268028000
6	-4.582832000	1.985370000	0.082566000
7	-3.339921000	2.203904000	-0.445866000
7	-5.033396000	0.817930000	0.456267000
7	-2.219292000	-1.303785000	-0.289406000
7	-4.294630000	-1.505128000	0.566033000
6	-3.117509000	-2.130760000	0.211075000
8	-1.265575000	1.522320000	-1.175831000
1	-3.059328000	3.148717000	-0.694480000
6	-5.497289000	-2.110171000	1.127396000
1	-6.340813000	-1.977850000	0.441205000
1	-5.311776000	-3.175141000	1.277109000
1	-5.738748000	-1.642456000	2.087163000
6	5.794702000	-1.904079000	0.032190000
1	5.322352000	-2.434681000	-0.798928000
1	6.841004000	-1.703149000	-0.211881000
1	5.744297000	-2.538420000	0.927565000
7	4.668493000	1.446254000	0.948046000
7	5.092881000	-0.651611000	0.239822000
6	5.576723000	0.507727000	0.830850000
8	1.746849000	2.527465000	0.565706000
1	0.414964000	0.823025000	-0.620709000
1	6.611299000	0.589433000	1.136982000
7	0.720172000	-1.559621000	-1.338584000
1	0.996284000	-2.524399000	-1.458248000
1	-0.250221000	-1.427918000	-1.051285000
1	-2.983661000	-3.196213000	0.344401000
1	-5.214142000	2.862479000	0.190110000

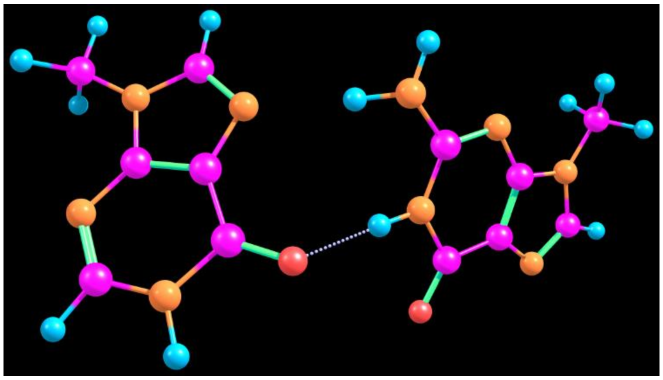

Syn-8BrI:anti-G entry 53

6	-3.057090000	1.563020000	-0.239450000
6	-4.272440000	0.814760000	-0.387860000
6	-4.284990000	-0.522380000	0.020660000
6	-2.155860000	-0.576150000	0.674690000
7	-3.271160000	-1.266410000	0.530030000
7	-2.031450000	0.746080000	0.329400000
6	1.467990000	2.232930000	0.575980000
6	2.194830000	1.056770000	0.246670000
6	3.510940000	1.171060000	-0.200640000
6	3.537710000	3.384660000	-0.124330000
7	2.246640000	3.390640000	0.335590000
7	4.222430000	2.312050000	-0.402870000
7	1.827140000	-0.269510000	0.331340000
7	3.961000000	-0.120070000	-0.388790000
6	2.892890000	-0.928170000	-0.050470000
8	0.305930000	2.345870000	1.032710000
1	1.783680000	4.270140000	0.527120000
6	5.297680000	-0.489220000	-0.839500000
1	6.033930000	0.065200000	-0.254060000
1	5.436720000	-1.559950000	-0.692030000
1	5.419940000	-0.244580000	-1.897970000
7	-5.514880000	1.186480000	-0.871140000
7	-5.563600000	-0.968940000	-0.215170000
6	-6.252200000	0.108890000	-0.755180000
1	-1.174550000	1.250740000	0.559620000
7	-1.058470000	-1.176070000	1.235500000
6	-6.057030000	-2.310280000	0.035530000
1	-7.131060000	-2.333610000	-0.163810000
1	-5.552340000	-3.031760000	-0.614840000
1	-5.877020000	-2.589770000	1.077590000
8	-2.806550000	2.737090000	-0.516040000
1	-0.128700000	-0.851370000	0.975000000
1	-1.152500000	-2.180510000	1.319430000
1	-7.294870000	0.026430000	-1.033960000
35	2.999660000	-2.792060000	-0.102900000
1	3.995290000	4.362500000	-0.257630000

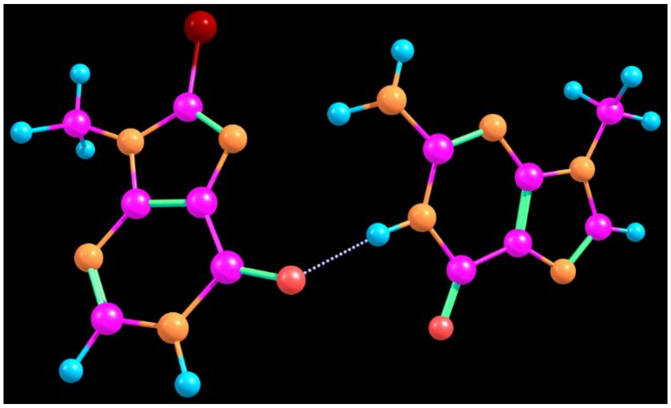

Reference

1. Zhao, Y.; Truhlar, D. G. Theoretical Chemistry Accounts 2008, 120, 215-241.
2. Grimme, S.; Antony, J.; Ehrlich, S.; Krieg, H. The Journal of Chemical Physics 2010, 132, 154104.
